# Stereocontrolled Total Synthesis of (−)‐Stemaphylline

**DOI:** 10.1002/anie.201611273

**Published:** 2017-01-18

**Authors:** Ana Varela, Lennart K. B. Garve, Daniele Leonori, Varinder K. Aggarwal

**Affiliations:** ^1^ School of Chemistry University of Bristol Cantock's Close Bristol BS8 1TS UK; ^2^ School of Chemistry University of Manchester Oxford Road Manchester M13 9PL UK

**Keywords:** boronic esters, lithiation–borylation, *Stemona* alkaloids, stereocontrol, total synthesis

## Abstract

Homologation of readily available α‐boryl pyrrolidines with metal carbenoids is especially challenging even when good leaving groups (Cl^−^) are employed. By performing a solvent switch from Et_2_O to CHCl_3_, efficient 1,2‐metalate rearrangement of the intermediate boronate occurs with both halide and ester leaving groups. The methodology was used in the total synthesis of the *Stemona* alkaloid (−)‐stemaphylline in just 11 steps (longest linear sequence), with high stereocontrol (>20:1 d.r.) and 11 % overall yield. The synthesis also features a late‐stage lithiation–borylation reaction with a tertiary amine containing carbenoid.

Homologation through lithiation–borylation has emerged as a powerful tool for the assembly of multiple stereogenic centers with high stereocontrol (Scheme 1 A),^1^ and the method has been applied in total synthesis, particularly for acyclic polyketide‐type natural products.^2^ Part of the power of the method stems from the ready availability of the coupling partners, namely alcohols and boronic esters. To significantly extend the reach of this chemistry to other classes of natural products, we considered its application to the synthesis of alkaloids, for example the *Stemona* family of alkaloids, a large class of natural products featuring a pyrrolo[1,2‐*a*]azepine moiety.^3^ This would require the use of lithiation–borylation on nitrogen‐containing boronic esters, a process which ultimately required considerable development.

**Scheme 1:**
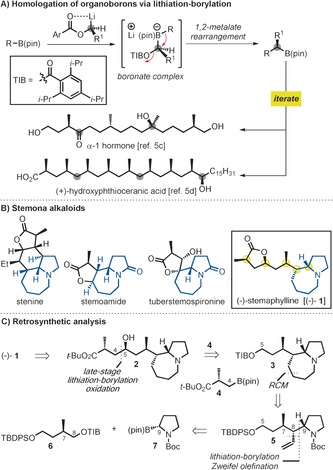
A) Homologation of organoboron compounds. B) Structures of *Stemona* alkaloids. C) Retrosynthetic analysis for (−)‐stemaphylline. pin=pinacolato; TIB=2,4,6‐triisopropyl benzoyl; TBDPS=*tert*‐butyldiphenylsilyl. Boc=*tert*‐butyloxycarbonyl; RCM=ring‐closing metathesis.

The *Stemonaceae* family of plants, from which the *Stemona* alkaloids have been isolated, have a long history of use in China and East Asia as traditional remedies for a range of ailments including respiratory diseases and infection with parasitic worms. For example, (−)‐stemaphylline ((−)‐**1**; Scheme 1 B), exhibits significant insecticidal and antiparasitic properties,^4^ and features the characteristic pyrrolo[*1,2a*]azepine core as well as a γ‐lactone and five stereogenic centers, three of which are contiguous. (−)‐Stemaphylline (−)‐**1** was recently synthesized (19 steps) but several steps showed poor stereocontrol and furthermore it was obtained as an inseparable mixture of diastereoisomers, although the corresponding *N*‐oxide was obtained in pure form.^5^ Herein, we report the development of lithiation–borylation on nitrogen‐containing boronic esters and its application to a concise, stereocontrolled, and scalable total synthesis of (−)‐**1** from readily available building blocks.

Our retrosynthetic analysis (Scheme 1 C) involved disconnection of the C4−C5 bond leading to the advanced intermediates, triisopropyl benzoate (TIB) ester **3** and boronic ester **4**. The coupling of **3** and **4** was anticipated to test the utility of lithiation–borylation in late‐stage assembly. Ester **3** could be constructed by ring‐closing metathesis (RCM) from the allyl pyrrolidine **5**. Pyrrolidine **5** could then be traced back to the simple building blocks **6** and **7**,^6^ where a tandem lithiation–borylation–Zweifel olefination^7^ sequence would be used to assemble the three contiguous stereogenic centers C7−C8−C9 in a single operation.

A key step is the lithiation–borylation reaction between building blocks **6** and **7**. Whilst potentially feasible, the use of α‐amino boronic esters in homologation reactions is notoriously troublesome, as documented by Matteson^8^ and Whiting^9^ (Scheme 2 A).^10^ The *N*‐Boc/*N*‐Ac pyrrolidine ring is a reluctant migrating group, even with exceptionally good leaving groups (Cl^−^), leading to unwanted side‐reactions and low conversions. Evidently, the adjacent electron‐withdrawing *N*‐Boc group makes the migrating carbon much less nucleophilic.^11^ Over the course of our investigations we have identified many reaction parameters that can be used to facilitate recalcitrant 1,2‐metalate rearrangements and we were keen to evaluate if they could be applied to the *N*‐Boc pyrrolidine system. As shown in Scheme 2 B, lithiation of TIB ester **8** and electrophilic trapping with *rac*‐**7** gave the boronate complex **9**, as determined by ^11^B NMR spectroscopy. Unsurprisingly, this intermediate proved to be stable under a variety of conditions including refluxing in Et_2_O or addition of the Lewis acid, MgBr_2_⋅Et_2_O (entries 1 and 2); no migration occurred in either case. However, under more forcing conditions, we found that treatment of **9** with MgBr_2_⋅Et_2_O under reflux gave boronic ester **10**, albeit in low yield (entry 3, 19 %). We have found that the solvent can sometimes affect the outcome of 1,2‐metalate rearrangements and decided to perform a solvent screen.^12^ This revealed that, in refluxing toluene, the efficiency of the 1,2‐shift **9**→**10** improved dramatically and **10** was obtained in 67 % yield (entry 4). The yield was further improved by using CHCl_3_ as the solvent (entry 5) which gave **10** in 85 % yield. By analyzing the rate of migration in TBME and CHCl_3_ at various temperatures, we constructed an Eyring plot which showed that the 1,2‐migration had a considerably lower enthalpy of activation in CHCl_3_ compared to TBME (28.7 vs. 34.2 kcal mol^−1^ respectively; see the Supporting Information for details) indicating a specific solvent effect. This is most likely to result from the enhanced Lewis acidity of Li^+^ in the less coordinating solvent,^13^ thereby promoting 1,2‐migration.

**Scheme 2:**
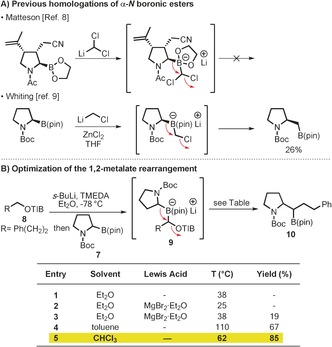
A) Previous homologations of α‐N boronic esters. B) Optimization of the α‐N 1,2‐metalate rearrangement. TMEDA=*N*,*N*,*N*′,*N*′‐tetramethylethylenediamine.

With conditions established using racemic **7**, enantioenriched **7** (98:2 e.r.)^6^, ^14^ was homologated with lithiated TIB esters (+)/(−)‐**8 a** giving the *syn* and *anti* diastereoisomers of **11** in high yield and with very high levels of enantio‐ and diastereocontrol (Scheme 3). Matteson homologation of **7** (ClCH_2_Li) also benefited from solvent exchange, enabling **12** to be obtained in a much improved 79 % yield without the use of further additives (compare Scheme 2 A). The C−B bond of **7** could also be functionalized through Zweifel olefination^7^ (**13**) in good yield and complete stereospecificity. Finally, this strategy was extended to the 2‐B(pin)‐*N*‐Boc‐piperidine building block **15** which was again homologated with lithiated TIB‐ester *rac*‐**8 a** to give **16** in excellent yield. Without solvent exchange to CHCl_3_, this substrate also performed poorly (no 1,2‐migration was observed after 24 h under reflux in Et_2_O) showing the general applicability of the new conditions.

**Scheme 3:**
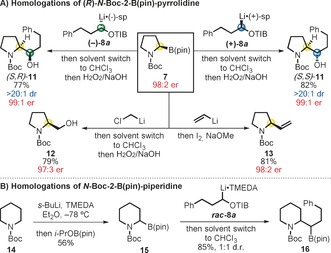
Stereospecific transformations of boronic ester **7**.

With suitable conditions established for the key step, the total synthesis of (−)‐**1** was pursued (Scheme 4). TIB ester **6** was prepared in two steps from commercially available diol **17**, by selective TBDPS protection^15^ and Mitsunobu esterification (70 % yield over 2 steps). Building blocks **6** and **7** were then coupled in the first key lithiation–borylation reaction which gave **18** in 58 % yield and 96:4 d.r. Following Zweifel olefination, pyrrolidine **5** was obtained in 71 % yield. Pleasingly, these two transformations could be carried out on a gram scale and in a one‐pot fashion, by adding the reagents sequentially, giving **5** in an improved 70 % yield (Scheme 3). With a short and scalable method for the assembly of the three contiguous stereocenters of (−)‐**1**, we moved to the formation of the pyrrolo[*1,2a*]azepine core. Upon removal of the TBDPS group of **5**, TIB ester **19** was formed under Mitsunobu conditions. *N*‐Boc deprotection and alkylation with 4‐bromo‐1‐butene gave diene **20** in 87 % yield over two steps. Treatment of **20** with the Hoveyda–Grubbs second‐generation catalyst in the presence of camphor sulfonic acid (CSA)^5^ promoted the desired RCM. Then, by simply adding PtO_2_ and a balloon of H_2_ to the crude RCM mixture, the desired saturated bicycle **3** was obtained as a single diastereoisomer in a single operation and 80 % overall yield.^16^

**Scheme 4:**
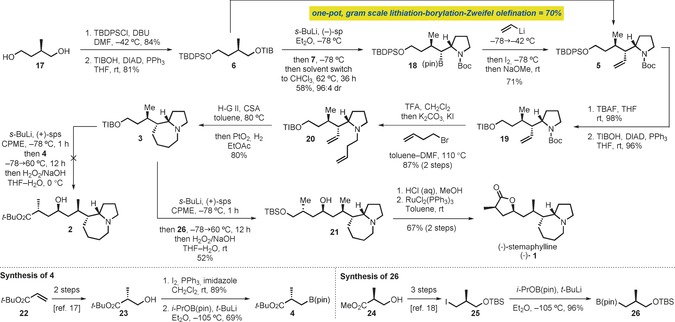
Total synthesis of (−)‐stemaphylline [(−)‐**1**]. DBU=1,8‐diazabycyclo[5.4.0]undecane; DMF=*N*,*N*‐dimethylformamide; DIAD=diisopropyl azadicarboxylate; (−)‐sp=(−)‐sparteine; TBAF=tetrabutylammonium fluoride; TFA=trifluoroacetic acid; H‐G II=Hoveyda‐Grubbs second‐generation catalyst; CSA=camphor sulfonic acid; (+)‐sps=(+)‐sparteine surrogate; CPME=cyclopentyl methyl ether.

We were now in a position to examine the key and final late‐stage lithiation–borylation reaction. However, the lithiation of TIB ester **3** proved difficult; standard conditions [*s*‐BuLi⋅(+)‐sp (1.0 equiv), Et_2_O, 5 h] with a MeOD quench returned starting material with no incorporation of deuterium. Unfortunately, increasing the amount of base up to 3 equivalents as well as changing the reaction solvent and temperature did not improve this step. Speculating that steric hindrance could be responsible for the poor result, we tested the less hindered chiral diamine ligand, (+)‐sparteine surrogate [(+)‐sps].^17^ With the aid of in situ IR spectroscopic monitoring (see the Supporting Information for details), we examined the lithiation of **3** with the *s*‐BuLi⋅(+)‐sps complex in various solvents, followed by trapping with Me_3_SnCl (Scheme 5). Pleasingly, using CPME as the solvent and 3.0 equiv of *s*‐BuLi⋅(+)‐sps at −78 °C gave full lithiation in less than 1 h, furnishing stannane **27** in 92 % isolated yield (entry 5). Under the same conditions, but using (+)‐sparteine instead of (+)‐sparteine surrogate gave about 35 % lithiation (entry 6).

**Scheme 5:**
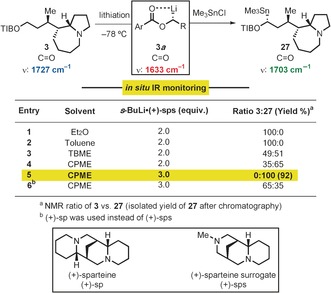
Optimization of lithiation of TIB ester **3**.

Having identified conditions for the asymmetric lithiation of **3**, we then turned to the building block **4**, which was prepared in two steps from alcohol **23** (2 steps from *t*‐butyl acrylate)^18^ through iodination (Appel, 89 %) and borylation (*t*‐BuLi, *i*‐PrOB(pin); 69 %). For the latter reaction, the use of in situ conditions (iodine–lithium exchange in the presence of *i*‐PrOB(pin)) gave superior yields of **4** than the ex situ conditions.

With the final substrates **3** and **4** in hand, we turned our attention to the pivotal coupling. Thus, lithiation of **3** and addition of boronic ester **4** led to immediate formation of a boronate complex as detected by ^11^B NMR. Unfortunately, despite all our efforts (addition of Lewis acids and solvent exchange), we were not able to promote the 1,2‐metalate rearrangement and the desired homologated product **2** could not be obtained. To determine whether there was an inherent problem with the use of boronic ester **4**, it was tested with the simple TIB ester **8** and smooth 1,2‐metalate rearrangement occurred leading to the product in good yield. This demonstrated that in a less challenging situation boronic ester **4** can be employed in lithiation–borylation reactions (see the Supporting Information for details).

As boronic esters bearing electron‐withdrawing functionalities (for example, **4** and **7**) are known to be poorer migrating groups,^19^ we reasoned that the less oxygenated coupling partner **26** might perform better in our late‐stage lithiation–borylation reaction. We then prepared building block **26** in one step from known iodide **25** 20 (3 steps from (*S*)‐Roche ester) using the same procedure (96 % yield) as for preparing **4**.^21^ Gratifyingly, the lithiation–borylation coupling of **4** and **26** was successful and, after oxidation, the desired secondary alcohol **21** was obtained in 52 % yield as a single diastereoisomer (Scheme 4). Subsequent silyl deprotection and selective oxidation of the primary alcohol^22^ furnished the γ‐lactone functionality and completed the total synthesis of (−)‐stemaphylline in 11 steps (longest linear sequence from commercially available diol **17**) with full stereocontrol.

In conclusion, we have found conditions under which *N*‐Boc α‐boryl pyrrolidine **7** can be used in lithiation–borylation reactions and have employed this reaction in a short stereocontrolled synthesis of (−)‐stemaphylline. By extending lithiation–borylation to including this readily available building block we have now opened up the method to not just polyketide‐like natural products, but also to a range of alkaloid natural products as well.

## Conflict of interest

The authors declare no conflict of interest.

## Supporting information

As a service to our authors and readers, this journal provides supporting information supplied by the authors. Such materials are peer reviewed and may be re‐organized for online delivery, but are not copy‐edited or typeset. Technical support issues arising from supporting information (other than missing files) should be addressed to the authors.

Supplementary

## References

[1a] D. Leonori , V. K. Aggarwal , Acc. Chem. Res. 2014, 47, 3174–3183; 25262745 10.1021/ar5002473

[1b] J. L. Stymiest , V. Baguski , R. M. French , V. K. Aggarwal , Nature 2008, 456, 778–782. 19079057 10.1038/nature07592

[2a] C. A. Brown , V. K. Aggarwal , Chem. Eur. J. 2015, 21, 13900–13903; 26332797 10.1002/chem.201503122 PMC6519258

[2b] A. Millán , J. R. Smith , V. K. Aggarwal , Angew. Chem. Int. Ed. 2016, 55, 2498–2502; 10.1002/anie.201511140 PMC4755224 26766494

[2c] A. P. Pulis , P. Fackler , V. K. Aggarwal , Angew. Chem. Int. Ed. 2014, 53, 4382–4385; 10.1002/anie.201400714 24634275

[2d] R. Rasappan , V. K. Aggarwal , Nat. Chem. 2014, 6, 810–814; 25143217 10.1038/nchem.2010

[2e] S. Balieu , G. E. Hallett , M. Burns , T. Bootwicha , J. Studley , V. K. Aggarwal , J. Am. Chem. Soc. 2015, 137, 4398–4403. 25625684 10.1021/ja512875g

[3a] R. A. Pilli , G. B. Rosso , M. d. C. Ferreira de Oliveira , Nat. Prod. Rep. 2010, 27, 1908–1937; 21042634 10.1039/c005018k

[3b] J. Chen , J. Chen , Y. Xie , N. Liu , D. Liu , H. Zhang , Chin. J. Org. Chem. 2013, 33, 1186–1194;

[3c] F. Wang , Q. Chen , Nat. Prod. Commun. 2014, 9, 1809–1822; 25632491

[3d] X. Liu , F. Wang , Nat. Prod. Commun. 2015, 10, 1093–1102; 26197559

[3e] S. Huang , F. Kong , Q. Ma , Z. Guo , L. Zhou , Q. Wang , H. Dai , Y. Zhao , J. Nat. Prod. 2016, 79, 2599–2605; 27684288 10.1021/acs.jnatprod.6b00528

[3f] A. Hager , N. Vrielink , D. Hager , J. Lefranca , D. Trauner , Nat. Prod. Rep. 2016, 33, 491–522. 26621771 10.1039/c5np00096c

[4] P. Mungkornasawakul , S. Chaiyong , T. Sastraruji , A. Jatisatienr , C. Jatisatienr , S. G. Pyne , A. T. Ung , J. Korth , W. Lie , J. Nat. Prod. 2009, 72, 848–851. 19374387 10.1021/np900030y

[5] M. L. Schulte , M. L. Turlington , S. S. Phatak , J. M. Harp , S. R. Stauffer , C. W. Lindsley , Chem. Eur. J. 2013, 19, 11847–11852. 23956045 10.1002/chem.201302669 PMC3925759

[6] Building block **7** is commercially available but can be prepared in one step using the method developed by Whiting: A. S. Batsanov , C. Grosjean , T. Schuetz , A. Whiting , J. Org. Chem. 2007, 72, 6276–6279. 17628110 10.1021/jo0708792

[7] G. Zweifel , H. Arzoumanian , C. C. Whitney , J. Am. Chem. Soc. 1967, 89, 3652–3653.

[8] D. S. Matteson , J. Lu , Tetrahedron: Asymmetry 1998, 9, 2423–2436.

[9] K. Arnold , A. S. Batsanov , B. Davies , C. Grosjean , T. Schuetz , A. Whiting , K. Zawatzky , Chem. Commun. 2008, 3879–3881. 10.1039/b806779a 18726021

[10] Curiously, Whiting found that reaction of 2-Li-*N*-Boc-pyrrolidine with ClCH_2_B(pin) in the presence of ZnCl_2_ gave the product in 69 % yield (Ref. [8]). In both cases, the same boronate complex is formed but reaction shown in Scheme 2 A is much less efficient.

[11] The natural charge (NBO) on the migrating carbon is −0.363 for the *N*-Boc pyrrolidine but substantially higher (−0.594) when the *N*-Boc pyrrolidine is replaced by *i*-Pr. See the Supporting Information for full details of computational analysis.

[12] S. Roesner , J. M. Casatejada , T. G. Elford , R. P. Sonawane , V. K. Aggarwal , Org. Lett. 2011, 13, 5740–5743. 21995597 10.1021/ol202251p

[13a] M. Samet , J. Buhle , Y. Zhou , S. R. Kass , J. Am. Chem. Soc. 2015, 137, 4678–4680; 25821991 10.1021/jacs.5b01805

[13b] C. Reichardt in Solvents and Solvent Effects in Organic Chemistry, Wiley-VCH, Weinheim, 2004, pp. 93–145;

[13c] C. Reichardt , in Solvents and Solvent Effects in Organic Chemistry, Wiley-VCH, Weinheim, 2004, pp. 147–328;

[13d] R. Díaz-Torres , S. Alvarez , Dalton Trans. 2011, 40, 10742–10750. 21927754 10.1039/c1dt11000d

[14] P. Beak , S. T. Kerrick , S. Wu , J. Chu , J. Am. Chem. Soc. 1994, 116, 3231–3239.

[15] R. Lagoutte , P. Sebesta , P. Jiros , B. Kalinova , A. Jirosova , J. Straka , K. Cerna , J. Sobotnik , J. Cvacka , U. Jahn , Chem. Eur. J. 2013, 19, 8515–8524. 23630024 10.1002/chem.201204196

[16] Using Pd/C in the hydrogenation reaction resulted in the formation of a mixture of diastereoisomers of **3**, presumably as a result of alkene isomerization prior to reduction. See Ref. [2c] and references therein.

[17a] M. J. Dearden , C. R. Firkin , J.-P. R. Hermet , P. O'Brien , J. Am. Chem. Soc. 2002, 124, 11870–11871; 12358529 10.1021/ja027774v

[17b] M. J. Dearden , M. J. McGrath , P. O'Brien , J. Org. Chem. 2004, 69, 5789–5792; 15307761 10.1021/jo049182w

[17c] M. J. McGrath , J. L. Bilke , P. O'Brien , Chem. Commun. 2006, 2607–2609. 10.1039/b603804m 16779493

[18] C. Pautigny , S. Jeulin , T. Ayad , Z. Zhang , J.-P. Genêt , V. Ratovelomanana-Vidal , Adv. Synth. Catal. 2008, 350, 2525–2532.

[19a] A. Bottoni , M. Lombardo , A. Neri , C. Trombini , J. Org. Chem. 2003, 68, 3397–3405; 12713337 10.1021/jo026733e

[19b] R. Larouche-Gauthier , C. J. Fletcher , I. Couto , V. K. Aggarwal , Chem. Commun. 2011, 47, 12592–12594; 10.1039/c1cc14469c 21892499

[19c] V. K. Aggarwal , G. Y. Fang , X. Ginesta , D. M. Howells , M. Zaja , Pure Appl. Chem. 2006, 78, 215–229.

[20] D. A. Hansen , C. M. Rath , E. B. Eisman , A. R. H. Narayan , J. D. Kittendorf , J. D. Mortison , Y. J. Yoon , D. H. Sherman , J. Am. Chem. Soc. 2013, 135, 11232–11238. 23866020 10.1021/ja404134f PMC3771335

[21] Attempts to convert iodide **25** into boronic ester **26** using copper catalysis as described by Ito and Marder were unsuccessful. H. Ito , K. Kubota , Org. Lett. 2012, 14, 890–893;22260229

[22a] K. B. Sharpless , K. Akashi , K. Oshima , Tetrahedron Lett. 1976, 17, 2503;

[22b] H. Tomioka , K. Takai , K. Oshima , H. Nozaki , Tetrahedron Lett. 1981, 22, 1605–1608.

