# Supplementary material for: Stereocontrolled Total Synthesis of (−)‐Stemaphylline
Source: Angew Chem Int Ed Engl. 2017 Jan 18;56(8):2127–31. doi: 10.1002/anie.201611273 (PMC5484348; doi:10.1002/anie.201611273)
Supplement: Supplementary file 1 — Supplementary [file ANIE-56-2127-s001.pdf]

Angewandte Chemie
Eine Zeitschrift der Gesellschaft Deutscher Chemiker

Supporting Information

Stereocontrolled Total Synthesis of (−)-Stemaphylline

Ana Varela, Lennart K. B. Garve, Daniele Leonori, and Varinder K. Aggarwal**

anie_201611273_sm_miscellaneous_information.pdf

Supporting Information

Table of Contents

1. General Experimental Details 3
2. Model Studies for the Lithiation-Borylation with Boronic Ester **7** 5
   2.1. Starting Material Synthesis 5
   2.2. Optimisation of the Reaction Conditions 7
   2.3. Kinetic studies for 1,2-metallate rearrangement 10
   2.4. Stereocontrolled Functionalizations of **7** 13
   2.5. Natural charge (NBO) calculations 25
3. Lithiation-Borylation with Boronic Ester **15** 28
4. Synthesis of Building Block **6** 30
5. Synthesis of **20** 38
6. Synthesis of **3** 42
7. Optimisation of the Asymmetric Lithiation of **3** 45
   7.1. Initial *in situ* React-IR Monitoring 45
   7.2. Optimization of the Asymmetric Lithiation of **3** Using (+)-Sparteine Surrogate 47
   7.3. *in situ* React-IR monitoring 48
8. Synthesis of Building Block **4** 51
   8.1. Model Lithiation-Borylation with Boronic Ester **4** 57
9. Synthesis of Building Block **26** 58
10. Endgame 61
11. ^{1}H-NMR and ^{13}C-NMR Spectra 66
12. References 89

1. General Experimental Details

All required fine chemicals were used directly without purification unless mentioned. All air- and water-sensitive reactions were carried out in flame-dried glassware under nitrogen atmosphere using standard Schlenk manifold technique. ^{1}H and ^{13}C Nuclear Magnetic Resonance (NMR) spectra were acquired at various field strengths as indicated, and were referenced to $CHCl_3$ (7.27 and 77.0 ppm for ^{1}H and ^{13}C respectively) or DMSO (2.50 and 39.5 ppm for ^{1}H and ^{13}C respectively). Chemical shifts (δ) are quoted in parts per million (ppm) and coupling constants (J) are in Hertz (Hz). Letters are assigned to protons in chemical structures for ^{1}H NMR assignment purposes only (assigning in alphabetical order from downfield to upfield). Carbons attached to boron atoms are often not observed due to quadrupolar relaxation. ^{11}B NMR spectra were measured using boron-free quartz NMR tubes, and recorded with complete proton decoupling using $BF_3 \cdot Et_2O$ (0.0 ppm) as an external standard. High-resolution mass spectra (HRMS) were recorded using Electron Spray Ionization (ESI). All IR data was obtained on a Perkin-Elmer Spectrum One FT-IR spectrometer. Optical rotations were obtained on a Perkin-Elmer 241MC polarimeter. Analytical TLC: Aluminium-backed plates pre-coated (0.25 mm) with Merck Silica Gel 60 F254. Compounds were visualized by exposure to UV-light or and/or developed with potassium permanganate or anisaldehyde. Flash column chromatography was performed using Aldrich Silica Gel 60 (40-63 μm). Chiral HPLC was performed using a Diacel Chiralpak IA or IB column (4.6 × 250 mm × 5 μm) fitted with the respective guard (4 × 10 mm) and monitored by DAD (Diode Array Detector). Chiral SFC was performed using a Chiralpak IA, IB, IC or Whelk-01 column (4.6 × 250 mm × 5 μm) on a Waters TharSFC system and monitored by DAD (Diode Array Detector). Chiral GC was performed using a Chiraldex β-DM column and monitored by FID (Flame Ionisation Detector). Solvents were purified by standard methods. TMEDA was distilled over CaH_2. (–)-Sparteine was obtained from the commercially available sulfate pentahydrate salt (99%, Acros) and isolated according to literature procedure[1]. (+)-Sparteine was purchased from BOC sciences and was distilled over CaH_2 prior to use. The sparteine free base readily absorbs atmospheric carbon dioxide (CO_2) and should be stored under argon/nitrogen at −20 °C in a Schlenk tube. (+)-Sparteine surrogate was synthesised from (–)-cytisine (purchased from Carbosynth) according to literature procedure[2] and was purified by kugelrohr distillation no more than 24 h prior to its use. *s*-BuLi was purchased from

Acros. *n*-BuLi and *t*-BuLi were purchased from Sigma-Aldrich. The molarity of organolithium solutions was determined by titration using benzylbenzamide[3]. Anhydrous DMF was purchased from Acros and stored over activated 4Å molecular sieves. 2-Isopropoxy-4,4,5,5-tetramethyl-1,3,2-dioxaborolane (*i*-PrOB(pin)) and 1,8-diazabicyclo[5.4.0]undec-7-ene (DBU) were purchased from Sigma-Aldrich and distilled under reduced pressure prior to their use. *(R)*-2-methylbutane-1,4-diol (**14**) was purchased from TCI. *In situ* IR monitoring was performed on a ReactIR15 instrument, equipped with a DST 9.5mm x 1.5m x 305mm probe with a DiComp (Diamond) sensor and a gold seal. For reverse phase purification of **37**, flash chromatography was performed on a Grace Discovery Sciences Reveleris Prep System with a Phenomenex Luna 12 g C_{18}(2) 100 Å AXIA packed column. The instrument was set to monitor the ELSD signal. Flow rates were 14 mL / min. The mobile phases used were 0.05 % formic acid in water for the aqueous phase and 0.05 % formic acid in MeCN for the organic phase. The gradient was from 5 % organic phase for 5 minutes at the start to 40 % organic phase over 10 minutes, then holding at 40 % organic phase for 10 minutes before rising to 80 % organic phase over 10 minutes, with 10 minutes at 100 % organic phase followed by 5 minutes at 5 % organic phase.

2. Model Studies for the Lithiation-Borylation with Boronic Ester 7

2.1. Starting Material Synthesis

3-Phenylpropyl 2,4,6-triisopropylbenzoate (8)

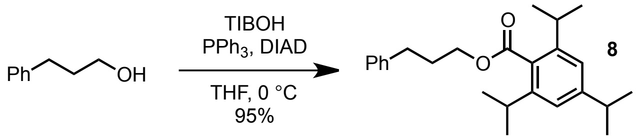

To a solution of 3-phenyl-1-propanol (0.90 g, 6.64 mmol, 1.10 equiv.) in THF (25 mL, 0.25 M) were added Ph_3P (1.74 g, 6.64 mmol, 1.10 equiv.) and 2,4,6-triisopropylbenzoic acid (1.50 g, 6.04 mmol, 1.00 equiv.). The mixture was cooled to 0 °C, then diisopropylazadicarboxylate (1.31 mL, 6.64 mmol, 1.10 equiv.) was added dropwise. The solution was stirred for 30 min at 0 °C, then it was warmed to room temperature and stirred for 4 h. Saturated NH_4Cl (10 mL) was added and the layers were separated. The organic layer was washed with Brine (2 x 10 mL) and the combined aqueous layers were extracted with Et_2O (3 x 10 mL). The organic layers were combined, dried over $MgSO_4$ and concentrated under vacuum. The crude product was triturated with petrol to remove Ph_3P=O, then it was purified by column chromatography on silica gel, eluting with petrol:EtOAc 95:5 to 90:10, to give **8** as a colourless oil (2.10 g, 95%). **^{1}H NMR** (400 MHz, $CDCl_3$): δ = 7.34-7.18 (5H, m), 7.00 (2H, s), 4.34 (2H, t, *J* 6.5 Hz), 2.88 (3H, sept, *J* 6.9 Hz), 2.75 (2H, t, *J* 7.8 Hz), 2.06 (2H, dt, *J* 7.8, 6.5 Hz), 1.27 (12H, d, *J* 6.9 Hz), 1.24 (6H, d, *J* 6.9 Hz). **^{13}C NMR** (100 MHz, $CDCl_3$): δ = 170.9 (C=O), 150.1 (C), 144.7 (2 x C), 141.1 (C), 130.6 (C), 128.5 (2 x CH), 128.4 (2 x CH), 126.1 (CH), 120.9 (2 x CH), 64.2 (CH_2), 34.4 (CH_2), 32.3 (CH), 31.5 (2 x CH), 30.4 (CH_2), 24.2 (4 x CH_3), 23.9 (2 x CH_3). Data in accordance with the literature.[4]

tert-Butyl 2-(4,4,5,5-tetramethyl-1,3,2-dioxaborolan-2-yl)pyrrolidine-1-carboxylate (7)

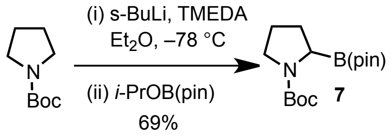

N-Boc-pyrrolidine (1.00 g, 5.84 mmol, 1.00 equiv.) and TMEDA (1.1 mL, 7.00 mmol, 1.20 equiv.) were dissolved in Et_2O (50 mL). The solution was cooled to –78 °C and *s*-BuLi (1.3 M in hexanes, 6.00 mL, 7.00 mmol, 1.20 equiv.) was added dropwise. The solution was stirred for 3 h at –78 °C, then *i*-PrOB(pin) (1.63 g, 8.80 mmol, 1.30 equiv.) was added dropwise. The solution was stirred for 1 h at –78 °C, then allowed to warm up to room temperature slowly. Aqueous 1 M HCl (50 mL) was added and the layers were separated. The organic layers were washed with Brine (3 x 20 mL) and the combined aqueous layers were extracted with Et_2O (3 x 50 mL). The organic layers were combined, dried over $MgSO_4$ and concentrated under vacuum. The crude product was purified by fast column chromatography on silica gel, eluting with petrol:EtOAc 85:15, to give **7** as a white solid (1.20 g, 69%). **^{1}H NMR** (500 MHz, DMSO-d_6, 90°C): δ = 3.23 (1H, m), 3.14 (1H, m), 2.80 (1H, m), 1.87 (2H, m), 1.74 (2H, m), 1.37 (9H, s), 1.19 (12H, m). **^{13}C NMR** (125 MHz, DMSO-d_6, 90°C): δ = 83.48 (2 x C), 78.54 (C), 46.42 (CH_2), 28.72 (3 x CH_3), 25.37 (CH_2), 25.18 (4 x CH_3), 24.83 (CH_2). C=O peak not observed. **^{11}B NMR** (96 MHz, $CDCl_3$): δ = 33. Data in accordance with the literature.[5]

Resolution between the enantiomers of **7** was achieved using chiral SFC analysis (Whelk 01 column, 10% IPA/hexane – iso 20%, 4 mL/min, 125 bar). t_R = 3.53 (*R*), 4.47 (*S*).

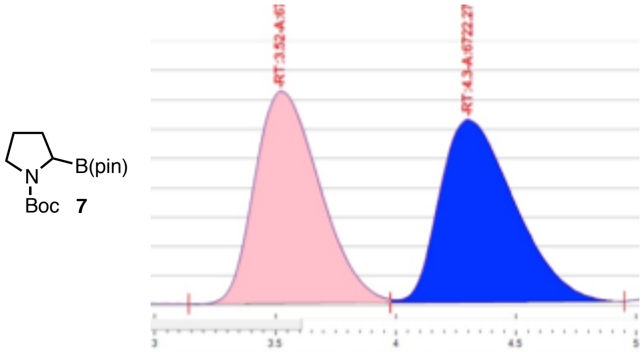

2.2. Optimisation of the Reaction Conditions

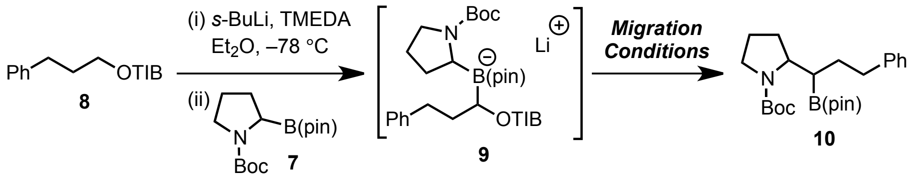

General Procedure for the Lithiation-Borylation Between TIB ester 8 and boronic ester 7 – GP1

8 (130 mg, 0.36 mmol, 1.10 equiv.) and TMEDA (0.05 mL, 0.32 mmol, 1.00 equiv.) were dissolved in Et_2O (3.5 mL). The solution was cooled to –78 °C and *s*-BuLi (1.3 M in hexanes, 0.28 mL, 0.32 mmol, 1.00 equiv.) was added dropwise. The solution was stirred for 3 h at –78 °C, then **7** (100 mg, 0.33 mmol, 1.05 equiv.) was added, dissolved in Et_2O (0.5 mL). The solution was stirred for 45 min at –78 °C, after which ^{11}B NMR analysis of the crude mixture showed complete formation of boronate complex **9** [^{11}B NMR (92.6 MHz, no solvent) δ = 7]. For conditions for the 1,2-metallate rearrangement from **9** to **10**, see Table 1 below.

Table 1. Optimisation of migration conditions from ate complex **9** to boronic ester **10**

Entry	Conditions	Yield (%)
**1**	Et_2O, rt, 12 h	–
**2**	Et_2O, reflux, 12 h	traces
**3**	$MgBr_2{\cdot}Et_2O$ (2.0 equiv.), Et_2O, rt, 12 h	traces
**4**	$MgBr_2{\cdot}Et_2O$ (2.0 equiv.), Et_2O, reflux, 12 h	19%
**5**	μW, Et_2O, 50 °C, 1 h	traces
**6**	μW, Et_2O, 100 °C, 1 h	56%
**7**	12-crown-4 (1.2 equiv.), Et_2O, rt, 1 h<br>then TMSCl (1.2 equiv.), Et_2O, rt, 12 h	–
**8**	H_2O (0.1 equiv.), Et_2O, rt, 1h<br>then TMSCl (1.2 equiv.), Et_2O, rt, 12 h	–
**9**	12-crown-4 (1.2 equiv.), H_2O (0.1 equiv.), Et_2O, rt, 1 h<br>then TMSCl (1.2 equiv.), Et_2O, rt, 12 h	–
**10**	Solvent exchange to $CHCl_3$, rt, 12 h	traces
**11**	Solvent exchange to $CHCl_3$, reflux, 12 h	85%
**12**	Solvent exchange to $CHCl_3$, $MgBr_2{\cdot}Et_2O$ (2.0 equiv.), reflux, 12 h	73%
**13**	Solvent exchange to toluene, rt, 12 h	traces
**14**	Solvent exchange to toluene, reflux, 12 h	67%

***tert*-Butyl 2-(3-phenyl-1-(4,4,5,5-tetramethyl-1,3,2-dioxaborolan-2-yl)propyl) pyrrolidine-1-carboxylate (10)**

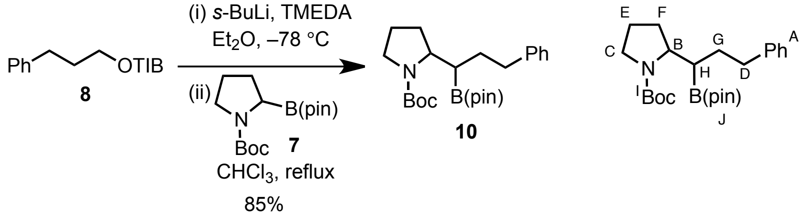

8 (200 mg, 0.55 mmol, 1.30 equiv.) and TMEDA (59 mg, 0.076 mL, 0.51 mmol, 1.20 equiv.) were dissolved in Et_2O (5.5 mL). The solution was cooled to –78 °C and *s*-BuLi (1.3 M in hexanes, 0.39 mL, 0.51 mmol, 1.20 equiv.) was added dropwise. The solution was stirred for 3 h at –78 °C, then **7** (125 mg, 0.42 mmol, 1.00 equiv.) was

added, dissolved in Et_2O (1 mL). The solution was stirred for 45 min at –78 °C, after which ^{11}B NMR analysis of the crude mixture showed complete boronate complex formation [^{11}B NMR (92.6 MHz, no solvent) δ = 7]. The solution was warmed to room temperature, Et_2O was removed under high vacuum and $CHCl_3$ was added. The solution was heated under reflux (70 °C) for 12 h. ^{11}B NMR analysis of the crude mixture showed full product formation [^{11}B NMR (92.6 MHz, no solvent) δ = 33]. Water (3 mL) was added and the layers were separated. The organic layer was washed with Brine (2 x 2 mL) and the combined aqueous layers were extracted with CH_2Cl_2 (3 x 5 mL). The organic layers were combined, dried over $MgSO_4$ and concentrated under vacuum. The crude product was purified by column chromatography on silica gel, eluting with petrol:EtOAc 95:5 to 90:10, to give **10** as a colourless oil (153 mg, 85%). **FT-IR** ν_{max} (film)/cm^{-1}: 2974, 1688, 1388, 1141, 698. **^{1}H NMR** (500 MHz, DMSO-d_6, 90°C): δ = 7.28-7.12 (5H, m, H^A), 3.84 (1H, m, H^B), 3.40 (1H, m, H^C), 3.09 (1H, m, H^C), 2.62 (1H, m, H^D), 2.50 (1H, m, H^D), 1.94-1.47 (7H, m, H^E & H^F & H^G & H^H), 1.36 (9H, s, H^I), 1.22 (12H, s, H^J). **^{13}C NMR** (125 MHz, DMSO-d_6, 90°C): δ = 154.4 (C=O), 143.0 (C), 128.6 (2 x CH), 128.5 (2 x CH), 125.9 (CH), 83.2 (2 x C), 78.4 (C), 58.9 & 58.6 (CH), 47.6 & 46.7 (CH_2), 35.8 & 35.4 (CH_2), 30.7 & 30.1 (CH_2), 27.7 (CH_2), 24.0 & 23.6 (CH_2). **^{11}B NMR** (96 MHz, $CDCl_3$): δ = 33. **HRMS** (ESI) Found: $[M+Na]^+$, 438.2791. $C_{24}H_{38}BNNaO_4$ requires $[M+Na]^+$, 438.2790.

2.3. Kinetic studies for 1,2-metallate rearrangement

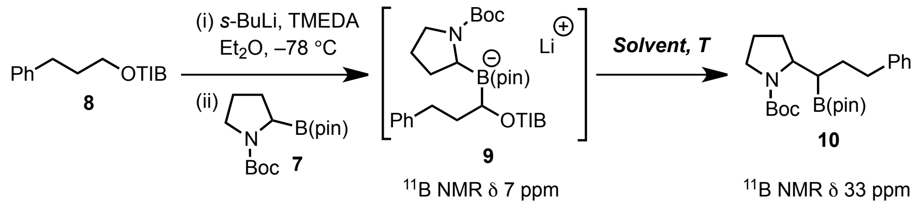

Preparation of 9: 8 (75 mg, 0.20 mmol, 1.20 equiv.) and TMEDA (0.03 mL, 0.19 mmol, 1.15 equiv.) we dissolved in Et_2O (3 mL). The solution was cooled to –78 °C and *s*-BuLi (1.3 M in hexanes, 0.15 mL, 0.19 mmol, 1.15 equiv.) was added dropwise. The solution was stirred for 3 h at –78 °C, then **7** (50 mg, 0.17 mmol, 1.00 equiv.) was added, dissolved in Et_2O (0.5 mL). The solution was stirred for 45 min at –78 °C, after which ^{11}B NMR analysis of the crude mixture showed complete boronate complex formation [^{11}B NMR (92.6 MHz, no solvent) $\delta = 7$]. All the volatiles were removed under high vacuum to give crude **9** that was used without further purification.

General Procedure for the ^{11}B NMR Kinetic studies – GP 2.

9 (0.17 mmol) was dissolved in the appropriate solvent (TBME or $CDCl_3$, 0.5 mL) and the resulting mixture was stirred for 5 min. The reaction mixture was transferred into a quartz NMR tube, immediately placed in a 300 MHz spectrometer pre-equilibrated at the desired temperature (24, 29, 35 or 45 °C) and the ^{11}B NMR acquisitions were started. The reaction progression was monitored every 30 min following the disappearance of **9** [^{11}B NMR (92.6 MHz, no solvent) $\delta = 7$] and the appearance of **10** [^{11}B NMR (92.6 MHz, no solvent) $\delta = 33$].

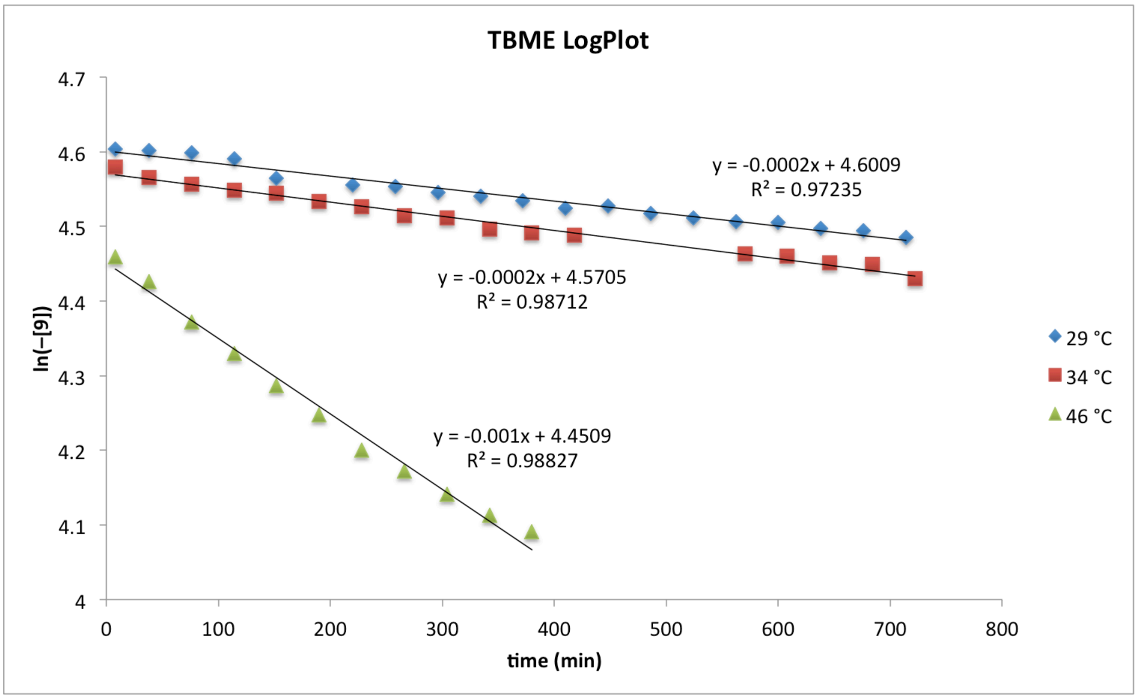

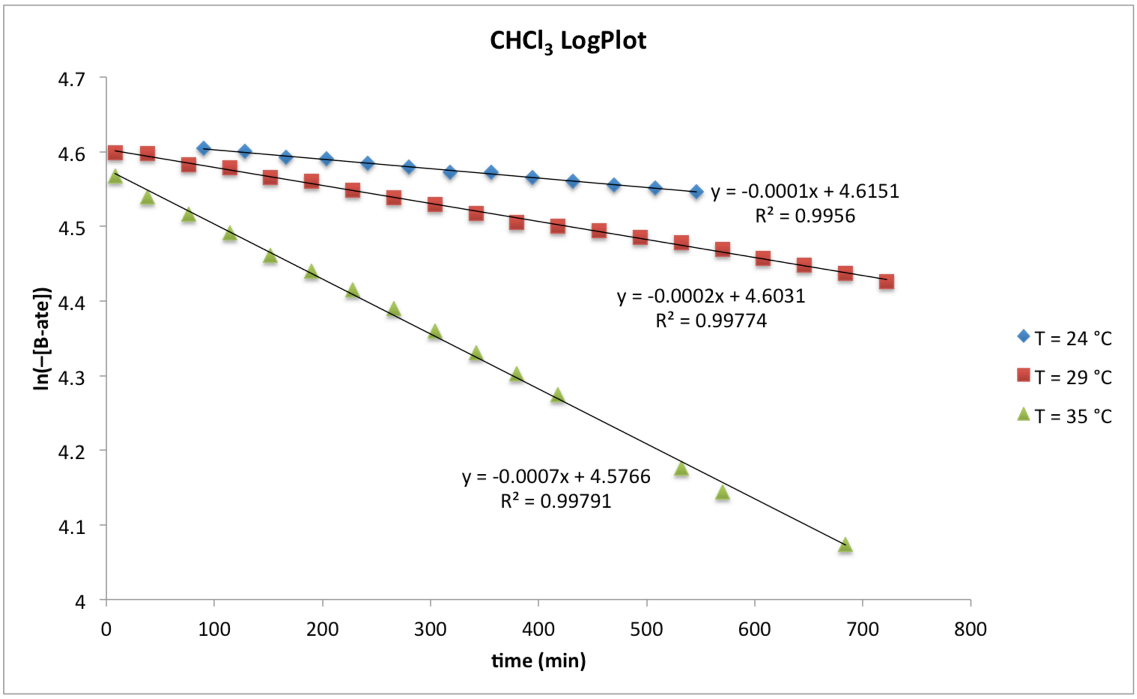

The observed rate constants at each temperature were introduced in the Eyring equation to obtain the $\Delta H^{\ddagger}$ and $\Delta S^{\ddagger}$ and entropy of the migration in each solvent:

$$ln\frac{k_{obs}}{T} = \frac{-\Delta H^{\neq}}{R} \cdot \frac{1}{T} + ln\frac{k_B}{h} \cdot \frac{\Delta S^{\neq}}{R}$$

Hence, plotting the logarithm of the quotient between the observed rate constant and the corresponding temperature versus the inverse of that temperature, the activation enthalpy can be obtained from the slope and the activation entropy from the intercept. The following Eyring plots were obtained:

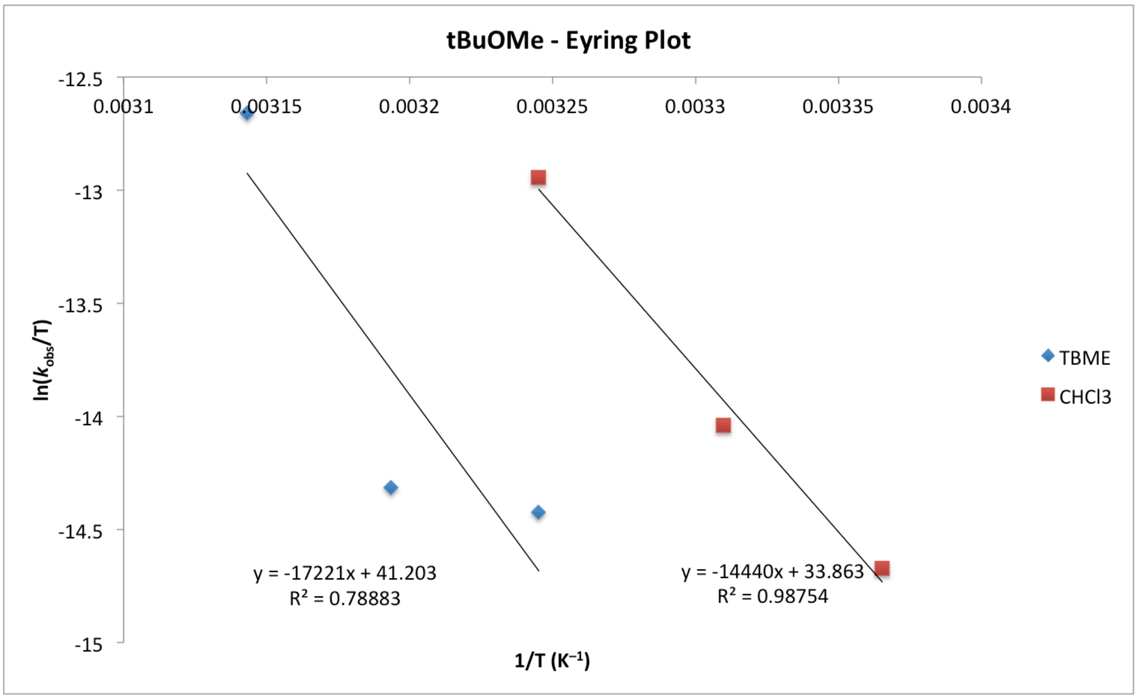

- **TBME**: $\Delta H^{\neq} = 34 \pm 17\ kcal, \Delta S^{\neq} = 0.04 \pm 0.08\ {}^{kcal}/_{K}$
- **CHCl₃**: $\Delta H^{\neq} = 27 \pm 3\ kcal, \Delta S^{\neq} = 0.02 \pm 0.01\ {}^{kcal}/_{K}$

Showing a negligible variation in activation entropy between the two solvents, but a change of approximately 7 kcal between the activation entropy in TBME and $CHCl_3$, indicating a significant solvent effect for the 1,2-metallate rearrangement.

2.4. Stereocontrolled Functionalizations of 7

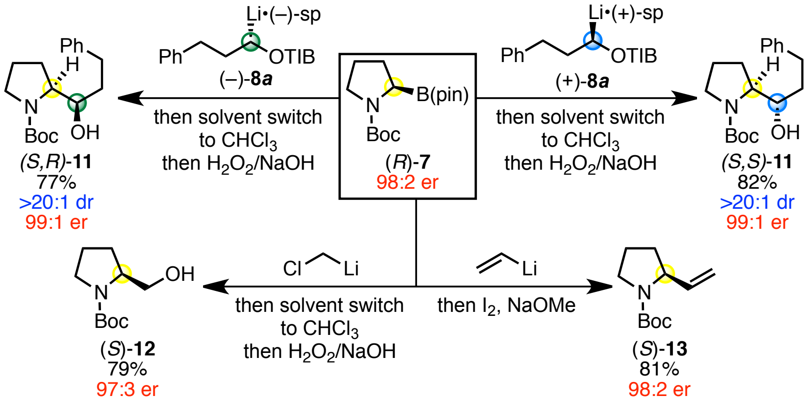

(*R*)-*tert*-Butyl 2-(4,4,5,5-tetramethyl-1,3,2-dioxaborolan-2-yl)pyrrolidine-1-carboxylate [(*R*)-7]

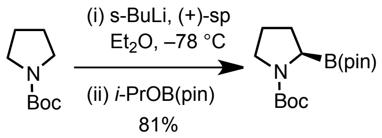

N-Boc-pyrrolidine (3.00 g, 17.5 mmol, 1.00 equiv.) and (+)-sparteine (4.93 g, 4.80 mL, 21.0 mmol, 1.20 equiv.) were dissolved in Et_2O (180 mL). The solution was cooled to –78 °C and *s*-BuLi (1.3 M in hexanes, 16.2 mL, 21.0 mmol, 1.20 equiv.) was added dropwise. The solution was stirred for 3 h at –78 °C, then *i*-PrOB(pin) (4.24 g, 22.8 mmol, 1.30 equiv.) was added dropwise. The solution was stirred for 1 h at –78 °C, then allowed to warm up to room temperature slowly. Aqueous 1 M HCl (150 mL) was added and the layers were separated. The organic layers were washed with Brine (3 x 50 mL) and the combined aqueous layers were extracted with Et_2O (3 x 100 mL). The organic layers were combined, dried over $MgSO_4$ and concentrated under vacuum. The crude product was purified by fast column chromatography on silica gel, eluting with petrol:EtOAc 85:15, to give (*R*)-**7** as a white solid (4.22 g, 81%, 98:2 e.r.). **[α]$_D$** (20 °C, $CHCl_3$, c 1.0) = –60 [lit.[5] for (*S*)-**7**, er 95:5, **[α]$_D$** (20 °C, $CHCl_3$, *c* 1.0) = +50.6]. Other data as above (section 2.1).

Chiral SFC analysis (Whelk 01 column, 10% IPA/hexane – iso 20%, 4 mL/min, 125 bar): t_R = 3.53 [major, (*R*)], 4.47 [minor, (*S*)].

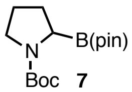

7

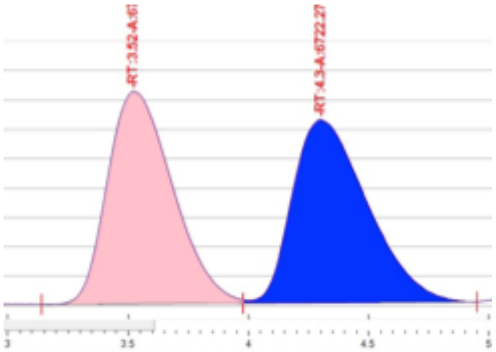

RT:3.52-A:6
RT:4.3-A:6722.27
3
3.5
4
4.5
5

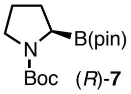

(*R*)-**7**

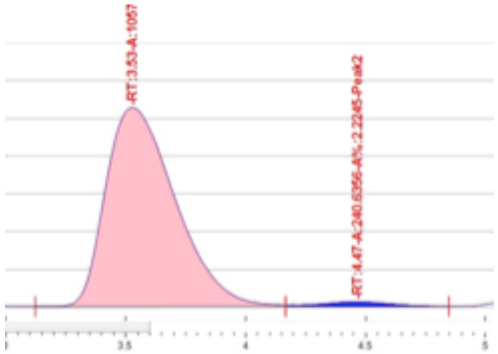

RT:3.53-A:1057
RT:4.47-A:240.6356-A%:2.2245-Peak2
3.5
4
4.5
5

tert-Butyl 2-(1-hydroxy-3-phenylpropyl)pyrrolidine-1-carboxylate (11)

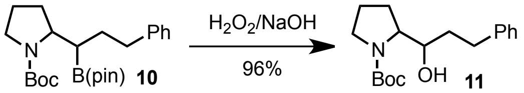

10 (75 mg, 0.18 mmol, 1.0 equiv.) was dissolved in THF (2 mL). The solution was cooled to 0 °C and an aqueous solution of 2 M NaOH and 30% H_2O_2 (2:1 v/v, 6 mL) was added dropwise. The solution was stirred vigorously for 2 h at 0 °C, then it was warmed to room temperature and diluted with water (10 mL) and EtOAc (10 mL). The layers were separated, the organic layer was washed with Brine (2 x 10 mL) and the combined aqueous layers were extracted with EtOAc (3 x 10 mL). The organic layers were combined, dried over $MgSO_4$ and concentrated under vacuum. The crude product was purified by column chromatography on silica gel, eluting with petrol:EtOAc 80:20, to give **11** as an oil (53 mg, 96%). See below for spectroscopic data of the pure diastereomers.

Resolution between the enantiomers and diastereomers of **11** was achieved using chiral SFC analysis (IC column, 50% IPA/hexane – iso 10%, 3 mL/min, 125 bar). t_R = 10.39, 12.03 (*(S,S)*-**11**), 14.47, 17.09 (*(S,R)*-**11**).

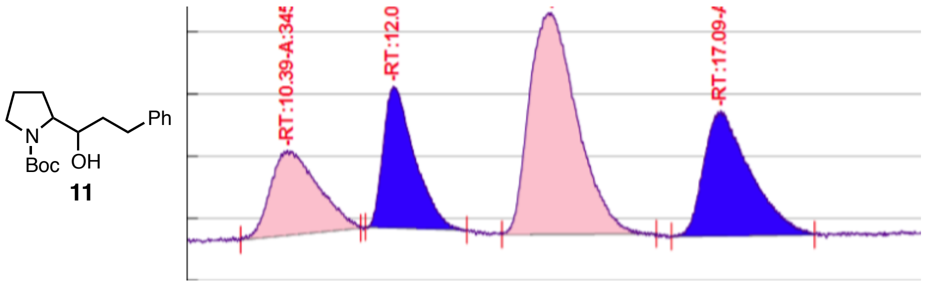

(*S*)-*tert*-Butyl 2-((*R*)-1-hydroxy-3-phenylpropyl)pyrrolidine-1-carboxylate [(*S, R*)-11]

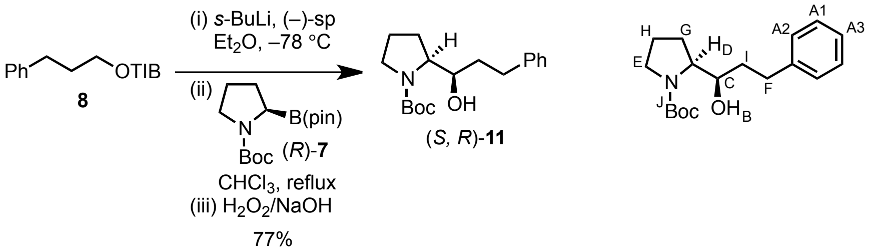

8 (200 mg, 0.55 mmol, 1.30 equiv.) and (–)-sparteine (119 mg, 0.12 mL, 0.51 mmol, 1.20 equiv.) we dissolved in Et_2O (5.5 mL). The solution was cooled to –78 °C and *s*-BuLi (1.3 M in hexanes, 0.39 mL, 0.51 mmol, 1.20 equiv.) was added dropwise. The solution was stirred for 3 h at –78 °C, then **7** (125 mg, 0.42 mmol, 1.00 equiv.) was

added, dissolved in Et_2O (1 mL). The solution was stirred for 45 min at –78 °C, after which ^{11}B NMR analysis of the crude mixture showed complete boronate complex formation [^{11}B NMR (92.6 MHz, no solvent) δ = 7]. The solution was warmed to room temperature, Et_2O was removed under high vacuum and $CHCl_3$ was added. The solution was heated under reflux (70 °C) for 12 h. ^{11}B NMR analysis of the crude mixture showed full product formation [^{11}B NMR (92.6 MHz, no solvent) δ = 33]. The solution was cooled to room temperature, $CHCl_3$ was removed under high vacuum and THF (6 mL) was added. The solution was cooled to 0 °C and an aqueous solution of 2 M NaOH and 30% H_2O_2 (2:1 v/v, 6 mL) was added dropwise. The solution was stirred vigorously for 2 h at 0 °C, then it was warmed to room temperature and diluted with water (10 mL) and EtOAc (10 mL). The layers were separated, the organic layer was washed with Brine (2 x 10 mL) and the combined aqueous layers were extracted with EtOAc (3 x 10 mL). The organic layers were combined, dried over $MgSO_4$ and concentrated under vacuum. The crude product was purified by column chromatography on silica gel, eluting with petrol:EtOAc 80:20, to give (*S,R*)-**11** as an oil (99 mg, 77%, 99:1 d.r., 99:1 e.r.). **[α]$_D$** (20 °C, $CHCl_3$, *c* 1.0) = –36. **FT-IR** ν_{max} (film)/cm^{-1}: 3417 (broad), 2971, 1666, 1396, 1162, 698. **^{1}H NMR** (500 MHz, DMSO-d_6, 90°C): δ = 7.25 (2H, t, *J* 7.4 Hz, H^{A1}), 7.19 (2H, d, *J* 7.4 Hz, H^{A2}), 7.14 (1H, t, *J* 7.4 Hz, H^{A3}), 4.38 (1H, d, *J* 5.5 Hz, H^B), 3.74 (1H, m, H^C), 3.62 (1H, m, H^D), 3.36 (1H, dt, *J* 10.5, 7.4 Hz, H^E), 3.17 (1H, ddd, *J* 10.5, 7.4, 6.2 Hz, H^E), 2.76 (1H, ddd, *J* 14.0, 8.2, 5.8 Hz, H^F), 2.58 (1H, dt, *J* 14.0, 8.2 Hz, H^F), 1.95 (1H, m, H^G), 1.86 (1H, dqnt, *J* 11.2, 7.8 Hz, H^H), 1.73 (1H, dq, *J* 11.8, 7.8 Hz, H^G), 1.67 (1H, m, H^H), 1.60 (2H, m, H^I), 1.35 (9H, s, H^J). **^{13}C NMR** (125 MHz, DMSO-d_6, 90 °C): δ = 142.8 (C), 128.7 (2 x CH), 128.5 (2 x CH), 125.9 (CH), 78.5 (C), 70.6 (CH), 62.1 (CH), 47.1 (CH_2), 36.4 (CH_2), 32.3 (CH_2), 28.7 (3 x CH_3), 25.5 (CH_2), 23.4 (CH_2), C=O peak not observed. **HRMS** (ESI) Found: $[M+Na]^+$, 328.1885. $C_{18}H_{27}NNaO_3$ requires $[M+Na]^+$, 328.1883.

(*S*)-*tert*-Butyl 2-((*S*)-1-hydroxy-3-phenylpropyl)pyrrolidine-1-carboxylate [(*S*,*S*)- 11]

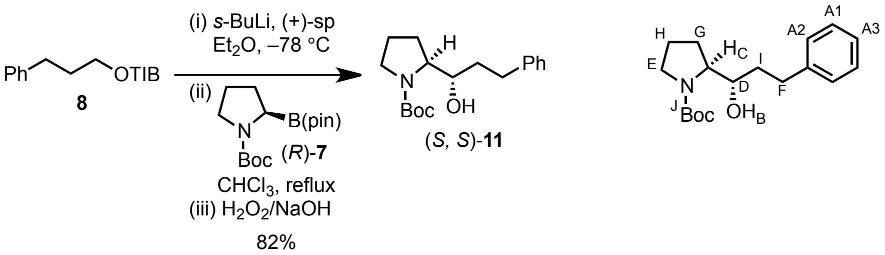

Following the same experimental procedure described for *(S,R)*-**11** using (+)-sparteine as the chiral diamine, *(S,S)*-**11** was obtained as an oil (105 mg, 82%, 99:1 d.r., 99:1 e.r.). **[α]D** (20 °C, $CHCl_3$, *c* 1.0) = –71. **FT-IR** ν_{max} (film)/cm^{-1}: 3377 (broad), 2973, 1663, 1394, 1161, 699. **^{1}H NMR** (500 MHz, DMSO-d_6, 90°C): δ = 7.24 (2H, t, *J* 7.5 Hz, H^{A1}), 7.18 (2H, d, *J* 7.5 Hz, H^{A2}), 7.44 (1H, t, *J* 7.5 Hz, H^{A3}), 4.43 (1H, br d, *J* 4.4 Hz, H^B), 3.81 (1H, q, *J* 5.7 Hz, H^C), 3.71 (1H, td, *J* 8.8, 5.7 Hz, H^D), 3.39 (1H, dt, *J* 10.7, 7.6 Hz, H^E), 3.17 (1H, dt, *J* 10.7, 6.6 Hz, H^E), 2.78 (1H, ddd, *J* 13.8, 9.4, 5.2 Hz, H^F), 2.58 (1H, dt, *J* 13.8, 8.0 Hz, H^F), 1.83 (2H, m, H^G), 1.78 (1H, m, H^H), 1.69 (1H, m, H^H), 1.61 (1H, m, H^I), 1.55 (1H, m, H^I), 1.36 (9H, s, H^J). **^{13}C NMR** (125 MHz, DMSO-d_6, 90°C): δ = 143.0 (C), 128.7 (2 x CH), 128.5 (2 x CH), 125.9 (CH), 78.8 (C), 71.5 (CH), 61.6 (CH), 47.5 (CH_2), 34.4 (CH_2), 32.3 (CH_2), 28.7 (3 x CH_3), 27.0 (CH_2), 24.0 (CH_2), C=O peak not observed. **HRMS** (ESI) Found: $[M+Na]^+$, 328.1887. $C_{18}H_{27}NNaO_3$ requires $[M+Na]^+$, 328.1883.

Chiral SFC analysis (IC column, 50% IPA/hexane – iso 10%, 3 mL/min, 125 bar): t_R = 10.39, 12.03 (*(S,S)*-**11**), 14.47, 17.09 (*(S,R)*-**11**).

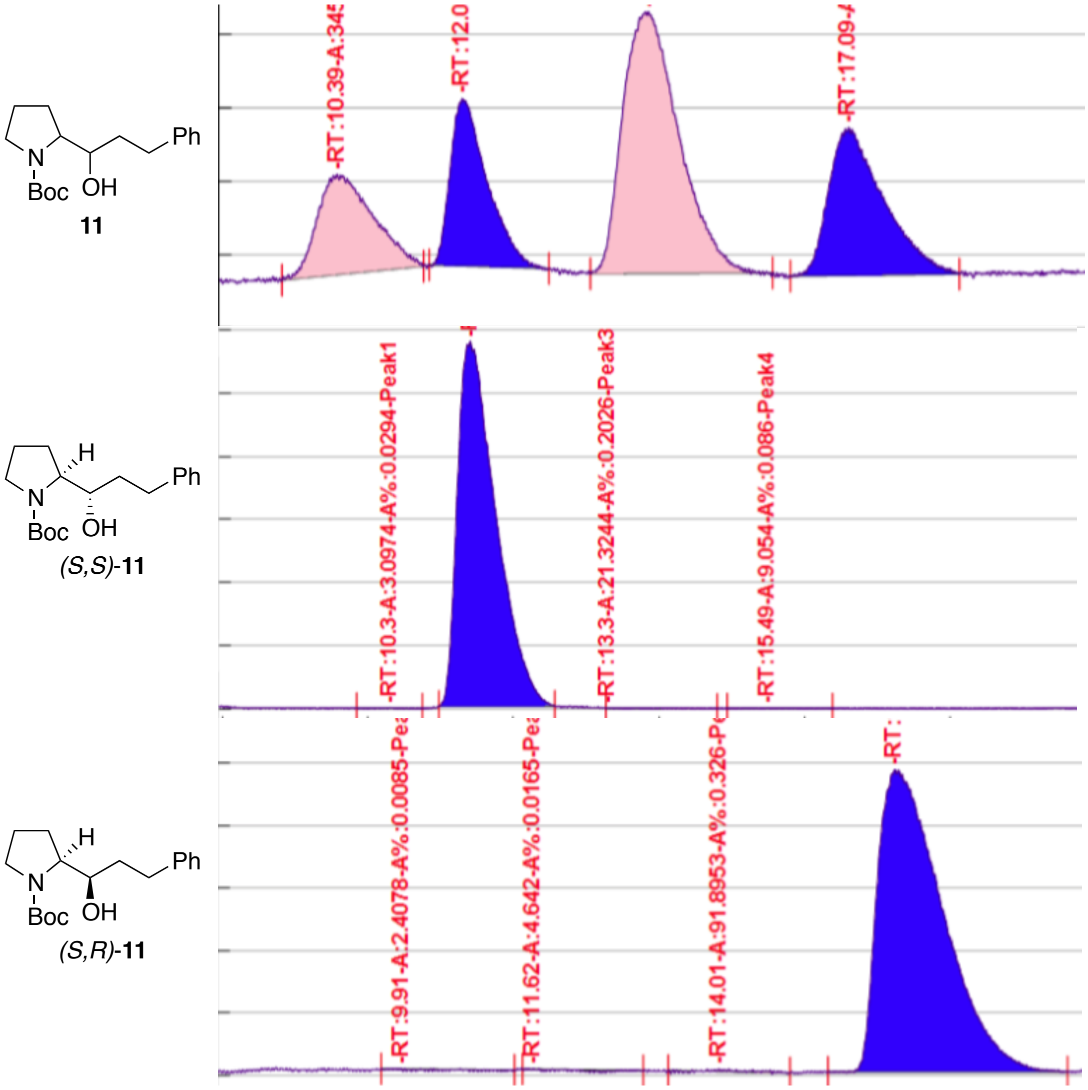

tert-Butyl 2-(hydroxymethyl)pyrrolidine-1-carboxylate (12)

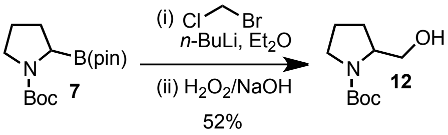

7 (200 mg, 0.67 mmol, 1.00 equiv.) and $BrCH_2Cl$ (261 mg, 0.14 mL, 2.00 mmol, 3.00 equiv.) were dissolved in Et_2O (6 mL). The solution was cooled to −78 °C and *n*-BuLi (1.6 M in hexanes, 1.0 mL, 1.68 mmol, 2.5 equiv.) was added dropwise (very slowly). The solution was stirred for 30 min at –78 °C, then it was warmed to room temperature. ^{11}B NMR analysis of the crude mixture showed complete boronate complex formation [^{11}B NMR (92.6 MHz, no solvent) δ = 7]. Et_2O was removed under high vacuum and $CHCl_3$ was added. The solution was heated under reflux (70 °C) for 20 h. ^{11}B NMR analysis of the crude mixture showed full product formation [^{11}B NMR (92.6 MHz, no solvent) δ = 33]. The solution was cooled to room temperature, $CHCl_3$ was removed under high vacuum and THF (6 mL) was added. The solution was cooled to 0 °C and an

aqueous solution of 2 M NaOH and 30% H_2O_2 (2:1 v/v, 6 mL) was added dropwise. The solution was stirred vigorously for 2 h at 0 °C, then it was warmed to room temperature and diluted with water (10 mL) and EtOAc (10 mL). The layers were separated, the organic layer was washed with Brine (2 x 10 mL) and the combined aqueous layers were extracted with EtOAc (3 x 10 mL). The organic layers were combined, dried over $MgSO_4$ and concentrated under vacuum. The crude product was purified by column chromatography on silica gel, eluting with petrol:EtOAc 80:20 to 70:30, to give **12** as an oil (70 mg, 52%). **^{1}H NMR** (500 MHz, DMSO-d_6, 90 °C): δ = 4.79 (1H, br s), 3.96 (1H, m), 3.60 (2H, m), 3.44 (1H, m), 3.32 (1H, m), 2.00 (1H, m), 1.88-1.71 (3H, m), 1.46 (9H, s). **^{13}C NMR** (125 MHz, DMSO-d_6, 90 °C): δ = 80.2 (C), 67.8 (CH_2), 60.2 (CH), 47.5 (CH_2), 28.7 (CH_2), 28.4 (3 x CH_3), 24.0 (CH_2), C=O peak not observed. Data in accordance with the literature[6].

Resolution of the enantiomers of **12** was achieved using chiral SFC analysis after derivatisation to **12*a*** (*vide infra*).

***tert*-Butyl 2-((benzoyloxy)methyl)pyrrolidine-1-carboxylate (12*a*)**

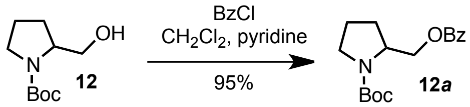

12 (30 mg, 0.15 mmol, 1.0 equiv.) was dissolved in CH_2Cl_2:pyridine (2:1 v/v, 1.5 mL). The solution was cooled to 0 °C and benzoyl chloride (31 mg, 0.026 mL, 0.22 mmol, 1.5 equiv.) was added dropwise. The mixture was warmed to room temperature and stirred for 6 h. Aqueous NH_4Cl (3 mL) was added and the layers were separated. The organic layer was washed with Brine (2 x 3 mL) and the combined aqueous layers were extracted with CH_2Cl_2 (3 x 5 mL). The organic layers were combined, dried over $MgSO_4$ and concentrated under vacuum. The crude product was purified by column chromatography on silica gel, eluting with petrol:EtOAc 97:3 to 95:5, to give **12*a*** as an oil (42 mg, 95%). **^{1}H NMR** (500 MHz, DMSO-d_6, 90 °C): δ = 7.96 (2H, d, *J* 7.5 Hz), 7.64 (1H, t, *J* 7.5 Hz), 7.52 (2H, t, *J* 7.5 Hz), 4.33 (2H, m), 4.07 (1H, m), 3.36 (1H, m), 3.27 (1H, m), 2.02 (1H, m), 1.95-1.77 (3H, m), 1.39 (9H, s). **^{13}C NMR** (125 MHz, DMSO-d_6, 90 °C): δ = 166.1 (C=O), 154.1 (C=O), 133.6 (CH), 130.4 (C), 129.6 (2 x CH), 129.1 (2 x CH), 79.1 (C), 65.5 (CH_2), 55.9 (CH), 46.8 (CH_2), 28.6 (3 x CH_3), 23.5 (CH_2), 19.3 (CH_3). Data in accordance with the literature.[7]

Resolution of the enantiomers of **12*a*** was achieved using chiral SFC analysis (Whelk-01 column, 10% IPA/hexane – iso 20%, 4 mL/min, 125 bar). t_R = 11.9 min, 15.6 min.

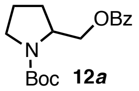

12*a*

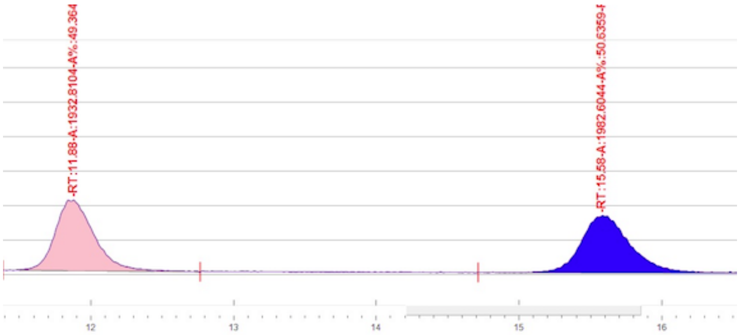

(*S*)-*tert*-Butyl 2-(hydroxymethyl)pyrrolidine-1-carboxylate [(*S*)-12]

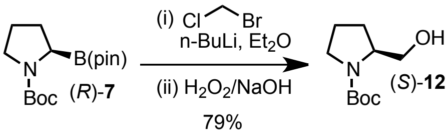

(*R*)-**7** (200 mg, 0.67 mmol, 1.00 equiv.) and $BrCH_2Cl$ (261 mg, 0.14 mL, 2.00 mmol, 3.00 equiv.) were dissolved in Et_2O (6 mL). The solution was cooled to −78 °C and *n*-BuLi (1.6 M in hexanes, 1.0 mL, 1.68 mmol, 2.5 equiv.) was added dropwise (very slowly). The solution was stirred for 30 min at –78 °C, then it was warmed to room temperature. ^{11}B NMR analysis of the crude mixture showed complete boronate complex formation [^{11}B NMR (92.6 MHz, no solvent) δ = 7]. Et_2O was removed under high vacuum and $CHCl_3$ was added. The solution was heated under reflux (70 °C) for 20 h. ^{11}B NMR analysis of the crude mixture showed full product formation [^{11}B NMR (92.6 MHz, no solvent) δ = 33]. The solution was cooled to room temperature, $CHCl_3$ was removed under high vacuum and THF (6 mL) was added. The solution was cooled to 0 °C and an aqueous solution of 2 M NaOH and 30% H_2O_2 (2:1 v/v, 6 mL) was added dropwise. The solution was stirred vigorously for 2 h at 0 °C, then it was warmed to room temperature and diluted with water (10 mL) and EtOAc (10 mL). The layers were separated, the organic layer was washed with Brine (2 x 10 mL) and the combined aqueous layers were extracted with EtOAc (3 x 10 mL). The organic layers were combined, dried over $MgSO_4$ and concentrated under vacuum. The crude product was purified by column chromatography on silica gel, eluting with petrol:EtOAc 80:20 to 70:30, to give (*S*)-**12** as an oil (105 mg, 79%, 97:3 e.r., 98% e.s. - determined after derivatisation to **12*a***, *vide infra*). **[α]$_D$** (20 °C, $CHCl_3$, *c* 1.0) = –35. Other data as above. Data in accordance with the literature[6].

When the reaction was carried out without solvent exchange (heating to reflux in Et_2O for 20 h), **12** was obtained in 40% yield (54 mg).

(*S*)-*tert*-Butyl 2-((benzoyloxy)methyl)pyrrolidine-1-carboxylate [(*S*)-12*a*]

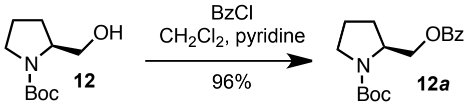

(*S*)-**12** (30 mg, 0.15 mmol, 1.0 equiv.) was dissolved in CH_2Cl_2:pyridine (2:1 v/v, 1.5 mL). The solution was cooled to 0 °C and benzoyl chloride (31 mg, 0.026 mL, 0.22 mmol, 1.5 equiv.) was added dropwise. The mixture was warmed to room temperature and stirred for 6 h. Aqueous NH_4Cl (3 mL) was added and the layers were separated. The organic layer was washed with Brine (2 x 3 mL) and the combined aqueous layers were extracted with CH_2Cl_2 (3 x 5 mL). The organic layers were combined, dried over $MgSO_4$ and concentrated under vacuum. The crude product was purified by column chromatography on silica gel, eluting with petrol:EtOAc 97:3 to 95:5, to give **12*a*** as an oil (44 mg, 96%, 97:3 e.r., 98% e.s.). Other data as above. Data in accordance with the literature.[7]

Chiral SFC analysis (Whelk-01 column, 10% IPA/hexane – iso 20%, 4 mL/min, 125 bar). t_R = 11.9 min [(*R*), minor], 15.6 min [(*S*), major].

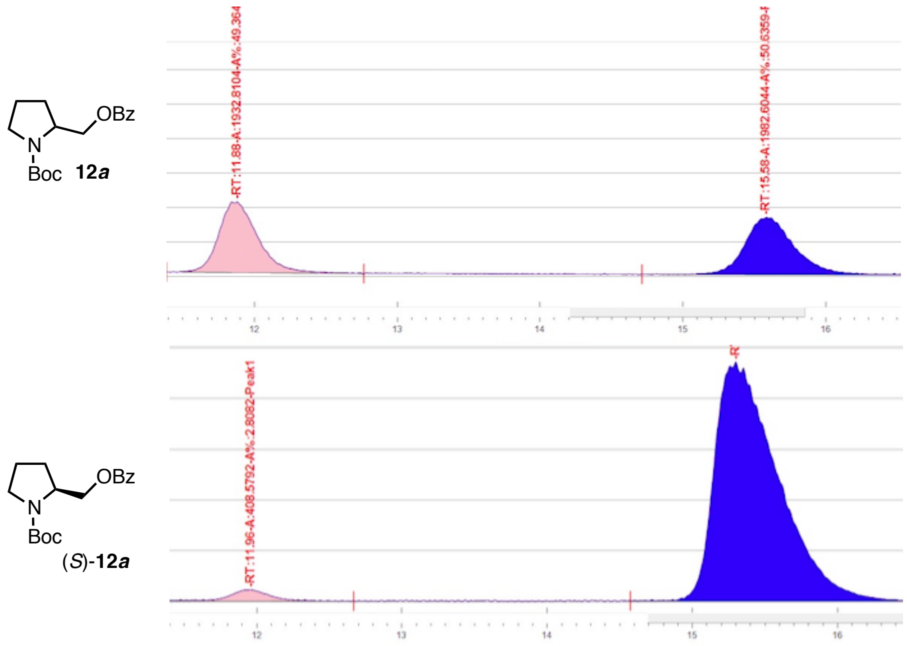

***tert*-Butyl 2-vinylpyrrolidine-1-carboxylate (13)**

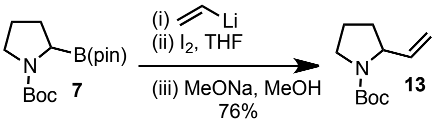

n-BuLi (1.6 M in hexanes, 1.26 mL, 2.01 mmol, 3.00 equiv.) was added dropwise to neat tetravinyltin (228 mg, 0.18 mL, 1.01 mmol, 1.50 equiv.) at room temperature under a N_2 atmosphere. The solution was stirred for 30 min, after which the stirring was

stopped, the white solid deposited and the colourless solution was decanted. The solid was washed with pentane (3 x 1 mL), stirring for 5 minutes and decanting the solution after each wash, then it was dissolved in THF (1.5 mL). **7** (200 mg, 0.67 mmol, 1.00 equiv.) was dissolved in Et_2O (5 mL) and cooled to –78 °C. The vinyl lithium solution was added dropwise. The solution was stirred for 30 min at –78 °C, then it was warmed to –42 °C and stirred for 20 min. ^{11}B crude NMR showed complete boronate complex formation [^{11}B NMR (92.6 MHz, no solvent) δ = 7]. The solution was cooled to –78 °C and a solution of iodine (533 mg, 2.01 mmol, 3.00 equiv.) in THF (2 mL) was added dropwise. The solution was stirred for 15 min at –78 °C, then a suspension of MeONa (216 mg, 4.02 mmol, 6.0 equiv.) in MeOH (2 mL) was added. The solution was warmed to room temperature and stirred for 1 h. Aqueous $Na_2S_2O_3$ (10 mL) was added and the layers were separated. The organic layer was washed with Brine (2 x 5 mL) and the combined aqueous layers were extracted with Et_2O (3 x 5 mL). The organic layers were combined, dried over $MgSO_4$ and concentrated under vacuum. The crude product was purified by column chromatography on silica gel, eluting with petrol:EtOAc 97:3 to 93:7, to give **13** as a colourless oil (100 mg, 76%). **^{1}H NMR** (500 MHz, DMSO-d_6, 90 °C): δ = 5.76 (1H, m), 4.99 (2H, m), 4.19 (1H, m), 3.27 (2H, m), 1.97 (1H, m), 1.76 (2H, m), 1.65 (1H, m), 1.38 (9H, s). **^{13}C NMR** (125 MHz, DMSO-d_6, 90 °C): δ = 154.0 (C=O), 139.8 (CH), 113.7 (CH_2), 78.6 (C), 59.0 (CH), 46.5 (CH_2), 31.7 (CH_2), 28.7 (3 x CH_3), 23.1 (CH_2). Data in accordance with the literature.[6]

Resolution of the enantiomers of 13 was achieved using chiral GC analysis (Chiraldex β-DM column, injector T = 250 °C, detector T = 300 °C, oven conditions: T = 70 °C for 3 min then ramp (0.5 °C/min) until 110 °C, hold for 5 min then ramp (15 °C/min) until 180 °C, He carrier gas at 1.0 mL/min, t_R (major) = 46.2 min, t_R (minor) = 47.9 min, total analysis time 93 min).

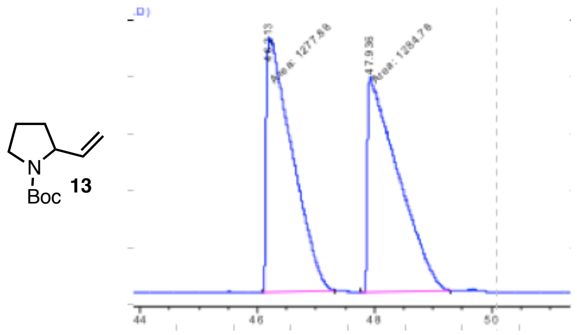

(*S*)-*tert*-Butyl 2-vinylpyrrolidine-1-carboxylate [(*S*)-13]

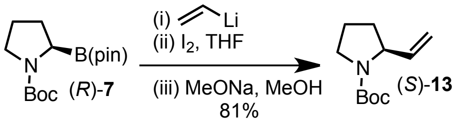

n-BuLi (1.6 M in hexanes, 1.26 mL, 2.01 mmol, 3.00 equiv.) was added dropwise to neat tetravinyltin (228 mg, 0.18 mL, 1.01 mmol, 1.50 equiv.) at room temperature under a N_2 atmosphere. The solution was stirred for 30 min, after which the stirring was stopped, the white solid deposited and the colourless solution was decanted. The solid was washed with pentane (3 x 1 mL), stirring for 5 minutes and decanting the solution after each wash, then it was dissolved in THF (1.5 mL). (*R*)-**7** (200 mg, 0.67 mmol, 1.00 equiv.) was dissolved in Et_2O (5 mL) and cooled to –78 °C. The vinyl lithium solution was added dropwise. The solution was stirred for 30 min at –78 °C, then it was warmed to –42 °C and stirred for 20 min. ^{11}B crude NMR showed complete boronate complex formation [^{11}B NMR (92.6 MHz, no solvent) δ = 7]. The solution was cooled to –78 °C and a solution of iodine (533 mg, 2.01 mmol, 3.00 equiv.) in THF (2 mL) was added dropwise. The solution was stirred for 15 min at –78 °C, then a suspension of MeONa (216 mg, 4.02 mmol, 6.0 equiv.) in MeOH (2 mL) was added. The solution was warmed to room temperature and stirred for 1 h. Aqueous $Na_2S_2O_3$ (10 mL) was added and the layers were separated. The organic layer was washed with Brine (2 x 5 mL) and the combined aqueous layers were extracted with Et_2O (3 x 5 mL). The organic layers were combined, dried over $MgSO_4$ and concentrated under vacuum. The crude product was purified by column chromatography on silica gel, eluting with petrol:EtOAc 97:3 to 93:7, to give (*S*)-**13** as a colourless oil (107 mg, 81%, 98:2 e.r., 100% e.s.). **[α]$_D$** (20 °C, $CHCl_3$, c 1.0) = –6. Other data as above. Data in accordance with the literature.[6]

Chiral GC analysis (Chiraldex β-DM column, injector T = 250 °C, detector T = 300 °C, oven conditions: T = 70 °C for 3 min then ramp (0.5 °C/min) until 110 °C, hold for 5 min then ramp (15 °C/min) until 180 °C, He carrier gas at 1.0 mL/min, t_R (major) = 46.2 min, t_R (minor) = 47.9 min, total analysis time 93 min):

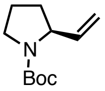

(*S*)-**13**

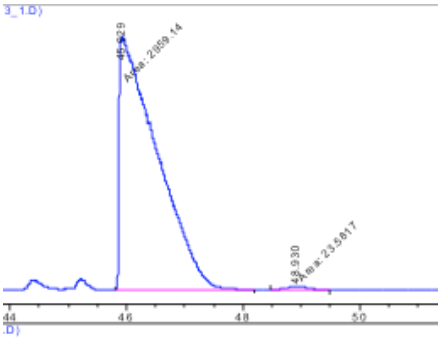
3_1.D)
48.930
Area: 23.5817
44
46
48
50

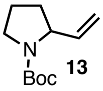

13

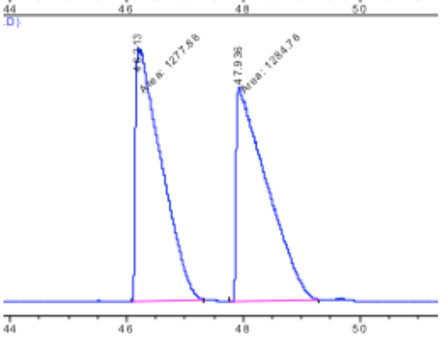
.D)
47.936
Area: 1284.76
44
46
48
50

2.5. Natural charge (NBO) calculations

Matteson homologation boron-ate complex

- Comparison between migrating groups – *N*-Boc pyrrolidine (**I**) vs. isopropyl (**II**)

I

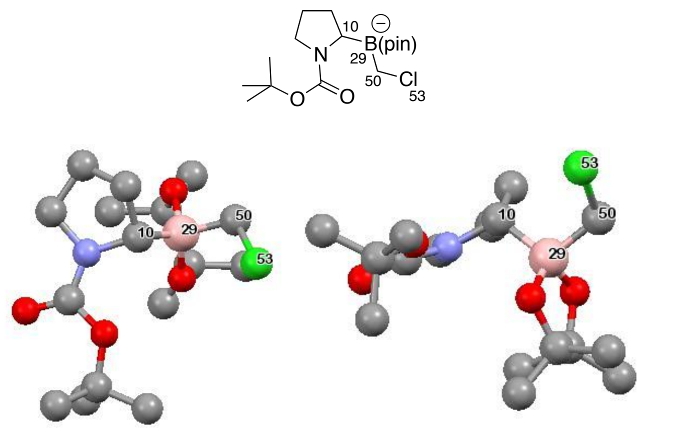

II

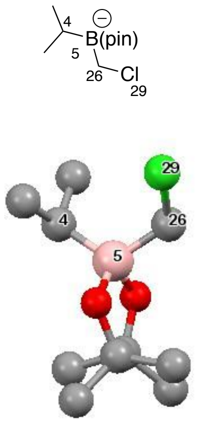

To further probe the substituent effect on the reactivity, theoretical calculations were performed. Molecular mechanics (MM) conformational searches were completed on **I** and **II** using the default MMFF force field in Spartan ‘14.[8] A number of conformers were selected for further geometry optimisation using the GAUSSIAN (version GAUSSIAN09)[9] software package and were carried out at the density functional level of theory, using the hybrid functional B3LYP.[10] For all elements, the split-valence double-zeta polarized basis set 6-31G* was employed. Calculations were performed on isolated molecules and NPA charge analysis was carried out by using NBO 3.1.[11] Vibrational frequencies were not computed. Results from NPA analysis are shown below. Note that only results from the lowest energy conformer are displayed, with conformers of higher energy showing negligible deviation in charges.

B3LYP/6-31+G* - NBO Charges

	**C_{10}**	**B_{29}**	**C_{50}**	**Cl_{53}**	**OBO fragm.**	**CH_2Cl fragm.**
**Pyrrolidine**	–0.363	0.949	–0.680	–0.196	–0.154	–0.421
	C_4	B_5	C_{26}	Cl_{29}	OBO fragm.	CH_2Cl fragm.
**Isopropyl**	–0.594	0.967	–0.680	–0.206	–0.162	–0.429

XYZ coordinates for calculated structures discussed

Pyrrolidine (**I**)

E = -1468.68539886 H

C	0.49287700	2.60220600	1.49087000
H	-0.26171400	3.09578200	2.12417700
H	1.42153900	2.54519400	2.06755400
C	0.67501700	3.39024000	0.18199600
H	1.61929100	3.09314200	-0.28345700
H	0.67919100	4.47887300	0.32401200
C	-0.50836100	2.93397300	-0.68508500
H	-0.26968500	2.89835900	-1.75438700
H	-1.38225600	3.59285600	-0.57297700
C	0.00103900	1.21185200	1.02176500
H	-0.68014100	0.78417600	1.76300100
N	-0.83113100	1.59860300	-0.16681100
C	-1.97080700	1.01148300	-0.58098900
O	-2.65730800	1.41589100	-1.53027600
O	-2.30103300	-0.05979300	0.19795200
C	-3.36177800	-0.98105500	-0.17587100
C	-3.10164700	-1.58015000	-1.56521400
H	-3.83529700	-2.37179400	-1.76808300
H	-2.09891700	-2.01852700	-1.59906900
H	-3.18016400	-0.81674900	-2.34259000
C	-3.25017100	-2.06950200	0.89916600
H	-3.41025000	-1.64258600	1.89559800
H	-2.25126400	-2.51622700	0.88133900
H	-4.00015900	-2.85223900	0.72745700
C	-4.73011700	-0.28615700	-0.10042800
H	-4.87192200	0.15875800	0.89221300
H	-5.53090600	-1.02002200	-0.26410600
H	-4.80617400	0.50222100	-0.85243300
B	1.22012700	0.11762700	0.75364300
O	2.37920800	0.64516600	-0.05507400
O	0.75699100	-1.05619600	-0.02593600
C	2.50491400	-0.08401900	-1.26658700
C	1.74546700	-1.44092000	-0.96370100
C	1.83855600	0.71534000	-2.40772600
H	2.27927100	1.71957600	-2.43536900
H	0.76420000	0.81903400	-2.23635300
H	1.99354300	0.24820300	-3.39000200
C	3.99807200	-0.24326300	-1.59730500
H	4.54622800	-0.69206300	-0.76393100
H	4.43561300	0.74471900	-1.78985700
H	4.15083100	-0.86201000	-2.49296500
C	1.04677900	-2.06476700	-2.18344200
H	0.55654400	-2.99945200	-1.88276700
H	1.76356300	-2.30065900	-2.98265800
H	0.27900000	-1.39933500	-2.58574700
C	2.66922800	-2.52269500	-0.35346600
H	3.27065200	-2.13128200	0.47044200
H	3.35130500	-2.95059800	-1.10148900
H	2.04117900	-3.32917700	0.04304800
C	1.91343600	-0.26924800	2.21925400
H	2.39699200	0.58850700	2.69863300
H	2.66136300	-1.06017600	2.13224100
Cl	0.75811500	-0.91678200	3.54030900

Isopropyl (**II**)

E = -1029.37521109 H

C	2.25128600	2.12200100	-0.79645200
H	2.69684400	3.08931300	-0.50402000
H	3.08442400	1.44547300	-1.03651700
C	1.36150000	1.53290300	0.31394300
B	0.52392300	0.19034100	-0.15995600
O	-0.56362400	0.52688600	-1.16518900
O	-0.24571200	-0.37132800	0.99363100
C	-1.83504300	0.40397300	-0.55909900
C	-1.58416400	-0.62351800	0.62202800
C	-2.29205200	1.78986600	-0.04344500
H	-2.24267000	2.50211100	-0.87628100
H	-1.63182700	2.15373900	0.74865500
H	-3.32276100	1.77437600	0.33902000
C	-2.84785400	-0.06937100	-1.61535800
H	-2.50802700	-0.98525900	-2.10739800
H	-2.95729800	0.70267100	-2.38805300
H	-3.83852300	-0.25264100	-1.17487800
C	-2.48059900	-0.41395700	1.85509500
H	-2.23387500	-1.16719400	2.61424700
H	-3.54687800	-0.51670700	1.60714200
H	-2.31594600	0.57145500	2.29998800
C	-1.74045900	-2.09637900	0.17116000
H	-1.18695900	-2.29703000	-0.74990700
H	-2.79231300	-2.37147700	0.00847400
H	-1.33034000	-2.74219000	0.95675400
C	1.41284500	-0.93884000	-0.99432900
H	1.82809300	-0.52670900	-1.92001600
H	0.82040500	-1.81769500	-1.25992100
Cl	2.90696500	-1.68782700	-0.14357300
H	1.67863300	2.28508700	-1.72008200
H	0.58427100	2.28792900	0.52644300
C	2.17214100	1.36231000	1.61290700
H	1.54213000	0.95779600	2.41495000
H	2.59402000	2.32253700	1.96169500
H	3.00966500	0.66737500	1.47398200

3. Lithiation-Borylation with Boronic Ester 15

***tert*-Butyl 2-(4,4,5,5-tetramethyl-1,3,2-dioxaborolan-2-yl)piperidine-1-carboxylate (15)**

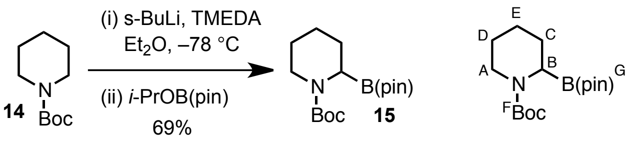

N-Boc-piperidine (**14**) (2.00 g, 10.8 mmol, 1.00 equiv.) and TMEDA (1.95 mL, 13.0 mmol, 1.20 equiv.) were dissolved in Et_2O (100 mL). The solution was cooled to –78 °C and *s*-BuLi (1.3 M in hexanes, 10.0 mL, 13.0 mmol, 1.20 equiv.) was added dropwise. The solution was stirred for 3 h at –78 °C, then *i*-PrOB(pin) (2.61 g, 2.86 mL, 14.0 mmol, 1.30 equiv.) was added dropwise. The solution was stirred for 1 h at –78 °C, then allowed to warm up to room temperature slowly. Aqueous 1 M HCl (150 mL) was added and the layers were separated. The organic layers were washed with Brine (3 x 50 mL) and the combined aqueous layers were extracted with Et_2O (3 x 100 mL). The organic layers were combined, dried over $MgSO_4$ and concentrated under vacuum. The crude product was purified by fast column chromatography on silica gel, eluting with petrol:EtOAc 85:15, to give **15** as an oil (1.89 g, 56%). **FT-IR** ν_{max} (film)/cm^{-1}: 2933, 1609, 1370, 1157. **^{1}H NMR** (400 MHz, $CDCl_3$): δ = 3.72 (1H, br d, *J* 12.7, H^A), 2.74 (1H, td, *J* 12.4, 2.93, H^A), 2.30 (1H, dd, *J* 12.6, 3.2, H^B), 1.81 (1H, m, H^C), 1.65-1.54 (2H, m, H^D & H^E), 1.49 (9H, s, H^F), 1.44 (1H, m, H^D), 1.40-1.29 (2H, m, H^C & H^E), 1.19 (12H, s, H^G). **^{13}C NMR** (100 MHz, $CHCl_3$): δ = 139.8 (C=O), 85.6 (2 x C), 79.9 (C), 42.4 (CH_2), 28.4 (3 x CH_3), 26.5 (CH_2), 25.1 (4 x CH_3), 24.9 (CH_2), 24.4 (CH_2). **^{11}B NMR** (96 MHz, $CDCl_3$): δ = 16. **HRMS** (ESI) Found: $[M+Na]^+$, 334.2170. $C_{20}H_{31}NNaO_3$ requires $[M+Na]^+$, 334.2160.

***tert*-Butyl 2-(3-phenyl-1-(4,4,5,5-tetramethyl-1,3,2-dioxaborolan-2-yl)propyl)piperidine-1-carboxylate (16)**

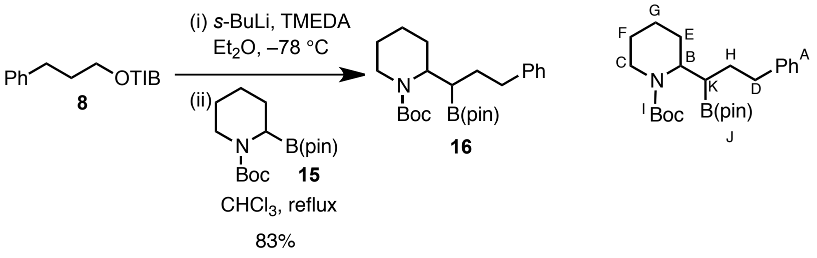

8 (200 mg, 0.55 mmol, 1.30 equiv.) and TMEDA (59 mg, 0.076 mL, 0.51 mmol, 1.20 equiv.) we dissolved in Et_2O (5.5 mL). The solution was cooled to –78 °C and *s*-BuLi (1.3 M in hexanes, 0.39 mL, 0.51 mmol, 1.20 equiv.) was added dropwise. The

solution was stirred for 3 h at –78 °C, then **15** (131 mg, 0.42 mmol, 1.00 equiv.) was added, dissolved in Et_2O (1 mL). The solution was stirred for 45 min at –78 °C, after which ^{11}B NMR analysis of the crude mixture showed complete boronate complex formation [^{11}B NMR (92.6 MHz, no solvent) δ = 7]. The solution was warmed to room temperature, Et_2O was removed under high vacuum and $CHCl_3$ was added. The solution was heated under reflux (70 °C) for 12 h. ^{11}B NMR analysis of the crude mixture showed full product formation [^{11}B NMR (92.6 MHz, no solvent) δ = 33]. Water (3 mL) was added and the layers were separated. The organic layer was washed with Brine (2 x 2 mL) and the combined aqueous layers were extracted with CH_2Cl_2 (3 x 5 mL). The organic layers were combined, dried over $MgSO_4$ and concentrated under vacuum. The crude product was purified by column chromatography on silica gel, eluting with petrol:EtOAc 95:5 to 90:10, to give **16** as a colourless oil (150 mg, 83%). **FT-IR** ν_{max} (film)/cm^{-1}: 2930, 1685, 1414, 1253, 1160, 1029, 698. **^{1}H NMR** (500 MHz, DMSO-d_6, 90°C): δ = 7.27-7.09 (5H, m, H^A), 4.22 (1H, app. d, *J* 11.6 Hz, H^B), 3.78 (1H, app. d, *J* 13.1 Hz, H^C), 2.64 (1H, m, H^D), 2.55 (1H, app. t, *J* 13.1 Hz, H^C), 2.44 (1H, m, H^D), 1.65-1.41 (8H, m, H^E & H^F & H^G & H^H), 1.35 (9H, s, H^I), 1.24 (12H, s, H^J), 1.18 (1H, m, H^K). **^{13}C NMR** (125 MHz, DMSO-d_6, 90°C): δ = 154.8 (C=O), 142.9 (C), 128.6 (2 x CH), 128.4 (2 x CH), 125.9 (CH), 83.5 (2 x C), 78.6 (C), 51.7 (CH), 38.5 (CH_2), 34.9 (CH_2), 30.2 (CH_2), 29.6 (CH_2), 28.7 (3 x CH_3), 25.6 (CH_2), 25.1 (4 x CH_3), 19.2 (CH_2). **^{11}B NMR** (96 MHz, $CDCl_3$): δ = 33. **HRMS** (ESI) Found: $[M+Na]^+$, 452.2941. $C_{25}H_{40}BNNaO_4$ requires $[M+Na]^+$, 452.2947.

4. Synthesis of Building Block 6

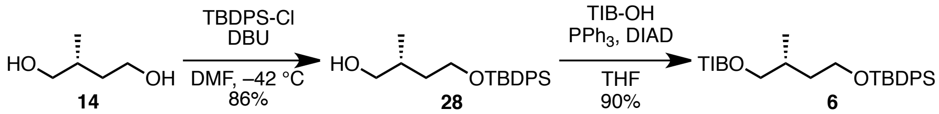

(R)-4-((*tert*-Butyldiphenylsilyl)oxy)-2-methylbutan-1-ol (28)

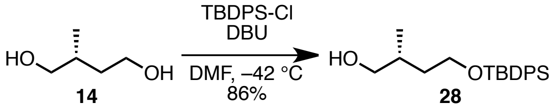

Following a literature procedure[12], a solution of *(R)*-2-methylbutane-1,4-diol (**14**) (500 mg, 0.51 mL, 4.80 mmol, 1.00 equiv.) in dry DMF (20 mL) was cooled to –42 °C. DBU (1.10 g, 1.08 mL, 7.20 mmol, 1.50 equiv.) was added, followed by a slow dropwise addition of *tert*-butyldiphenylsilyl chloride (1.39 g, 1.29 mL, 5.04 mmol, 1.05 equiv.). The solution was stirred for 8 h at –42 °C, then aqueous $NaHCO_3$ (20 mL) was added. The solution was diluted with Et_2O and the layers were separated. The organic layer was washed with Brine (10 mL) and the combined aqueous layers were extracted with Et_2O (3 x 10 mL). The organic layers were combined, dried over $MgSO_4$ and concentrated under vacuum. The crude product was purified by column chromatography on silica gel, eluting with petrol:EtOAc 90:10 to 80:20, to give **28** as a colourless oil (1.42 g, 86%). **^{1}H NMR** (400 MHz, $CDCl_3$): δ = 7.70-7.65 (4H, m), 7.47-7.36 (6H, m), 3.80-3.65 (2H, m), 3.54-3.44 (2H, m), 1.90-1.79 (1H, m), 1.69-1.58 (1H, m), 1.54-1.44 (1H, m), 1.05 (9H, s), 0.90 (3H, d, *J* = 6.5 Hz). **^{13}C NMR** (100 MHz, $CDCl_3$): δ = 135.6 (4 x CH), 133.4 (2 x C), 129.6 (2 x CH), 127.7 (4 x CH), 68.3 (CH_2), 63.8 (CH_2), 36.7 (CH_2), 33.9 (CH), 26.8 (3 x CH_3), 19.1 (C), 17.2 (CH_3). Data in accordance with the literature[12].

(R)-4-((*Tert*-butyldiphenylsilyl)oxy)-2-methylbutyl 2,4,6-triisopropylbenzoate (6)

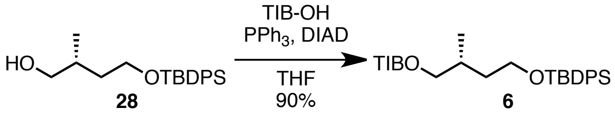

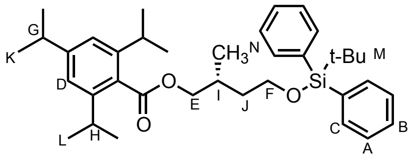

To a solution of **28** (1.24 g, 3.6 mmol, 1.1 equiv.) in THF (15 mL, 0.25 M) were added Ph_3P (944 mg, 3.6 mmol, 1.1 equiv.) and 2,4,6-triisopropylbenzoic acid (813 mg, 3.3 mmol, 1.0 equiv.). The mixture was cooled to 0 °C, then diisopropylacadicarboxylate (729 mg, 0.71 mL, 3.6 mmol, 1.1 equiv.) was added dropwise. The solution was stirred

for 30 min at 0 °C, hen it was warmed to room temperature and stirred for 4 h. Saturated NH_4Cl (10 mL) was added and the layers were separated. The organic layer was washed with Brine (2 x 10 mL) and the combined aqueous layers were extracted with Et_2O (3 x 10 mL). The organic layers were combined, dried over $MgSO_4$ and concentrated under vacuum. The crude product was triturated with petrol, then it was purified by column chromatography on silica gel, eluting with petrol:EtOAc 99:1 to 97:3, to give **6** as a colourless oil (1.70 g, 90%). **$[\alpha]_D$** (20 °C, $CHCl_3$, *c* 1.0) = –3. **FT-IR** ν_{max} (film)/cm^{-1}: 2960, 1724, 1461, 1249, 1104, 1074, 700. **^{1}H NMR** (400 MHz, $CDCl_3$): δ = 7.69-7.63 (4H, m, H^A), 7.45-7.33 (6H, m, H^B & H^C), 7.00 (2H, s, H^D), 4.22 (1H, dd, *J* = 10.9, 5.5 Hz, H^E), 4.11 (1H, dd, *J* = 10.9, 6.7 Hz, H^E), 3.73 (2H, m, H^F), 2.90 (1H, sept, *J* = 6.8 Hz, H^G), 2.85 (2H, sept, *J* = 6.8 Hz, H^H), 2.14 (1H, m, H^I), 1.76 (1H, m, H^J), 1.44 (1H, m, H^J), 1.26 (6H, d, *J* = 6.8 Hz, H^K), 1.24 (12H, d, *J* = 6.8 Hz, H^L), 1.05 (9H, s, H^M), 0.97 (3H, d, *J* = 6.4 Hz, H^N). **^{13}C NMR** (100 MHz, $CDCl_3$): δ = 171.2 (C=O), 150.0 (C), 144.7 (2 x C), 135.5 (4 x CH), 133.8 (2 x C), 130.7 (C), 129.6 (2 x CH), 127.6 (4 x CH), 120.8 (2 x CH), 70.0 (CH_2), 61.5 (CH_2), 36.0 (CH_2), 34.4 (CH), 31.5 (2 x CH), 29.3 (CH), 26.8 (3 x CH_3), 24.2 (2 x CH_3), 23.9 (4 x CH_3), 19.2 (C), 16.9 (CH_3). **HRMS** (ESI) Found: $[M+Na]^+$, 595.3596. $C_{37}H_{52}NaO_3Si$ requires $[M+Na]^+$, 595.3578.

4. Synthesis of 5 *via* Lithiation-Borylation-Zweifel Olefination

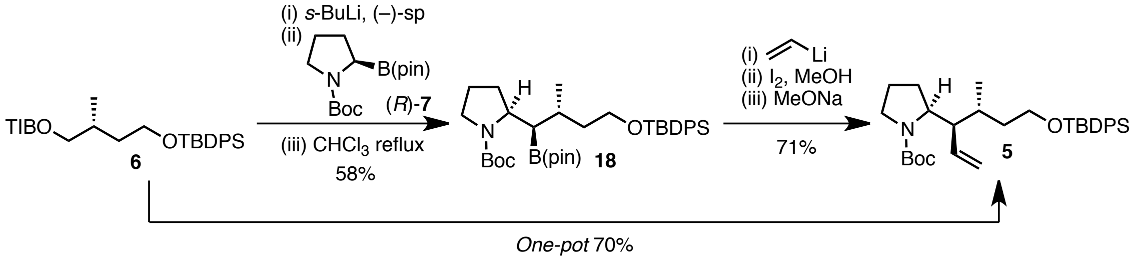

***tert*-Butyl (*S*)-2-((1R,2R)-4-((*tert*-butyldiphenylsilyl)oxy)-2-methyl-1-(4,4,5,5-tetramethyl-1,3,2-dioxaborolan-2-yl)butyl)pyrrolidine-1-carboxylate (18)**

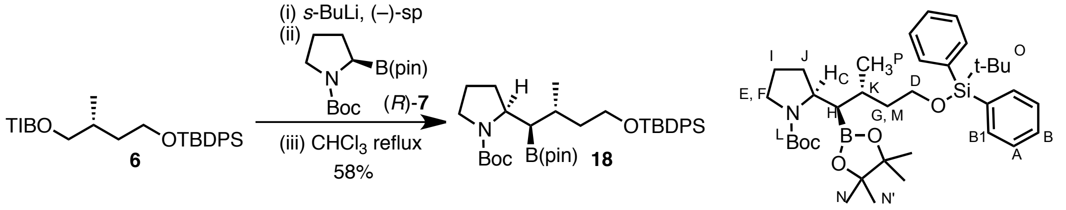

6 (100 mg, 0.17 mmol, 1.30 equiv.) and (–)-sparteine (37.5 mg, 0.037 mL, 0.16 mmol, 1.20 equiv.) were dissolved in Et_2O (2 mL, 0.1 M). The solution was cooled to –78 °C and *s*-BuLi (1.3 M in hexanes, 0.12 mL, 0.16 mmol, 1.20 equiv.) was added dropwise. The solution was stirred for 3 h 30 min at –78 °C, then (*R*)-**7** (40 mg, 0.13 mmol, 1.00 equiv.) was added, dissolved in Et_2O (0.5 mL). The solution was stirred for 45 min at –78 °C, after which ^{11}B NMR analysis of the crude mixture showed complete boronate complex formation [^{11}B NMR (92.6 MHz, no solvent) δ = 7]. The solution was warmed to room temperature, Et_2O was removed under high vacuum and $CHCl_3$ was added. The solution was heated under reflux (70 °C) for 36 h. ^{11}B NMR analysis of the crude mixture showed full product formation [^{11}B NMR (92.6 MHz, no solvent) δ = 33]. Water (3 mL) was added and the layers were separated. The organic layer was washed with Brine (2 x 2 mL) and the combined aqueous layers were extracted with CH_2Cl_2 (3 x 5 mL). The organic layers were combined, dried over $MgSO_4$ and concentrated under vacuum. The crude product was purified by column chromatography on silica gel, eluting with petrol:EtOAc 97:3 to 90:10, to give **18** as a colourless oil (47 mg, 58%, 96:4 d.r.). **[α]$_D$** (20 °C, $CHCl_3$, *c* 1.0) = –27. **FT-IR** ν_{max} (film)/cm^{-1}: 2930, 1690, 1388, 1106, 701. **^{1}H NMR** (500 MHz, DMSO-d_6, 90°C): δ = 7.62 (4H, m, H^A), 7.42 (6H, m, H^B & H^{B1}), 3.85 (1H, m, H^C), 3.71 (2H, m, H^D), 3.44 (1H, m, H^E), 3.08 (1H, m, H^F), 1.93-1.56 (7H, m, H^G & H^H & H^I & H^J & H^K), 1.39 (9H, s, H^L), 1.32 (1H, m, H^M), 1.19 (6H, s, H^N), 1.17 (6H, s, $H^{N'}$), 1.02 (9H, s, H^O), 0.84 (3H, d, *J* 6.5 Hz, H^P). **^{13}C NMR** (125 MHz, DMSO-d_6, 90 °C): δ = 155.4 (C=O), 135.4 (4 x CH), 134.3 (2 x

C), 130.2 (2 x CH), 128.1 (4 x CH), 83.0 (2 x C), 78.4 (C), 62.7 (CH_2), 57.5 (CH), 46.8 (CH_2), 39.4 (CH_2), 35.4 (CH), 29.6 (CH_2), 29.3 (CH), 28.7 (3 x CH_3), 27.3 (3 x CH_3), 25.2 (2 x CH_3), 25.1 (2 x CH_3), 23.9 (CH_2), 19.7 (CH_3), 19.2 (C). **^{11}B NMR** (96 MHz, $CDCl_3$): δ = 33. **HRMS** (ESI) Found: $[M+Na]^+$, 644.3893, $[M+H]^+$, 622.4076. $C_{36}H_{56}NaBNO_5Si$ requires $[M+Na]^+$, 644.3893, $C_{36}H_{56}BNO_5Si$ requires $[M+H]^+$, 622.4100.

(2*S*)-*tert*-Butyl 2-((2*R*)-4-((*tert*-butyldiphenylsilyl)oxy)-2-methyl-1-(4,4,5,5-tetramethyl-1,3,2-dioxaborolan-2-yl)butyl)pyrrolidine-1-carboxylate (18*b*)

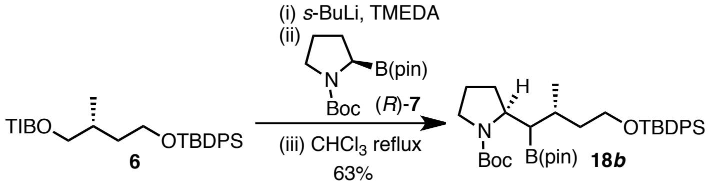

6 (150 mg, 0.26 mmol, 1.30 equiv.) and TMEDA (0.035 mL, 0.24 mmol, 1.20 equiv.) were dissolved in Et_2O (2 mL, 0.1 M). The solution was cooled to –78 °C and *s*-BuLi (1.3 M in hexanes, 0.18 mL, 0.24 mmol, 1.20 equiv.) was added dropwise. The solution was stirred for 3 h 30 min at –78 °C, then (*R*)-**7** (60 mg, 0.20 mmol, 1.00 equiv.) was added, dissolved in Et_2O (0.5 mL). The solution was stirred for 45 min at –78 °C, after which ^{11}B NMR analysis of the crude mixture showed complete boronate complex formation [^{11}B NMR (92.6 MHz, no solvent) δ = 7]. The solution was warmed to room temperature, Et_2O was removed under high vacuum and $CHCl_3$ was added. The solution was heated under reflux (70 °C) for 36 h. ^{11}B NMR analysis of the crude mixture showed full product formation [^{11}B NMR (92.6 MHz, no solvent) δ = 33]. Water (3 mL) was added and the layers were separated. The organic layer was washed with Brine (2 x 2 mL) and the combined aqueous layers were extracted with CH_2Cl_2 (3 x 5 mL). The organic layers were combined, dried over $MgSO_4$ and concentrated under vacuum. The crude product was purified by column chromatography on silica gel, eluting with petrol:EtOAc 97:3 to 90:10, to give **18*b*** as a colourless oil (78 mg, 63%). Characterisation data as above.

***tert*-Butyl 2-((2*R*)-4-((*tert*-butyldiphenylsilyl)oxy)-2-methyl-1-(4,4,5,5-tetramethyl-1,3,2-dioxaborolan-2-yl)butyl)pyrrolidine-1-carboxylate (18*a*)**

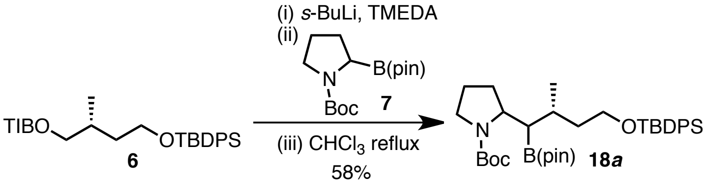

6 (100 mg, 0.26 mmol, 1.30 equiv.) and TMEDA (0.037 mL, 0.24 mmol, 1.20 equiv.) were dissolved in Et_2O (2 mL, 0.1 M). The solution was cooled to –78 °C and *s*-BuLi (1.3 M in hexanes, 0.18 mL, 0.24 mmol, 1.20 equiv.) was added dropwise. The solution was stirred for 3 h 30 min at –78 °C, then **7** (60 mg, 0.20 mmol, 1.00 equiv.) was added, dissolved in Et_2O (0.5 mL). The solution was stirred for 45 min at –78 °C, after which ^{11}B NMR analysis of the crude mixture showed complete boronate complex formation [^{11}B NMR (92.6 MHz, no solvent) δ = 7]. The solution was warmed to room temperature, Et_2O was removed under high vacuum and $CHCl_3$ was added. The solution was heated under reflux (70 °C) for 36 h. ^{11}B NMR analysis of the crude mixture showed full product formation [^{11}B NMR (92.6 MHz, no solvent) δ = 33]. Water (3 mL) was added and the layers were separated. The organic layer was washed with Brine (2 x 2 mL) and the combined aqueous layers were extracted with CH_2Cl_2 (3 x 5 mL). The organic layers were combined, dried over $MgSO_4$ and concentrated under vacuum. The crude product was purified by column chromatography on silica gel, eluting with petrol:EtOAc 97:3 to 90:10, to give **18** as a colourless oil (72 mg, 58%). Characterisation data as above.

Resolution between the diastereomers in **18*a*** and **18*b*** was achieved using chiral SFC analysis (Whelk-01 column, 10% IPA/Hexane – iso 20%, 4 mL/min, 125 bar). t_R 7.29, 8.28, 8.77, 10.96 (major, **18**).

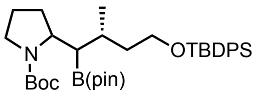

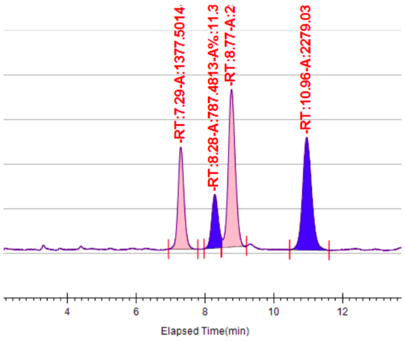

18*a*
4 diastereomers

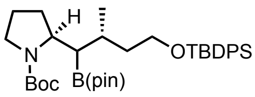

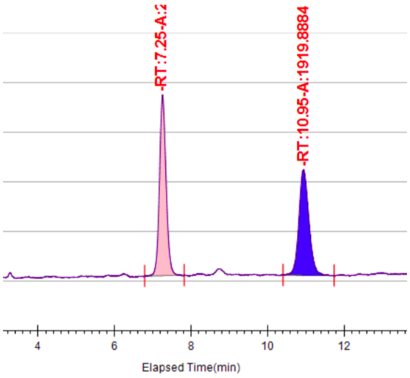

18*b*
2 diastereomers

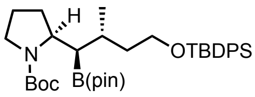

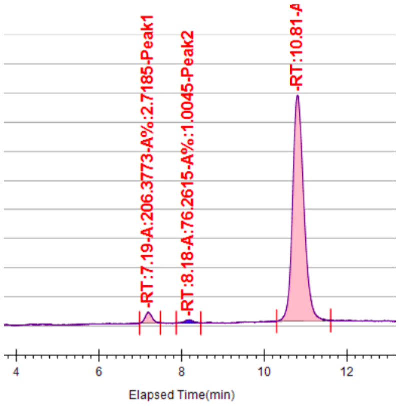

18
single
diastereomer

***tert*-Butyl (*S*)-2-((3R,4R)-6-((*tert*-butyldiphenylsilyl)oxy)-4-methylhex-1-en-3-yl)pyrrolidine-1-carboxylate (5)**

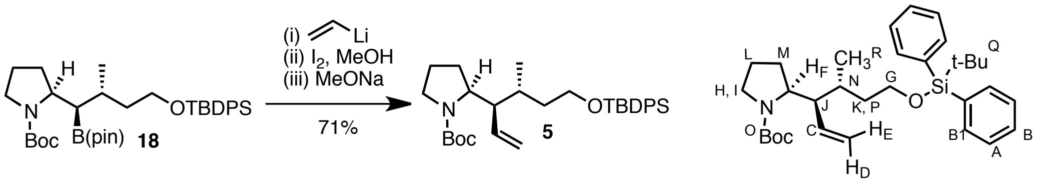

n-BuLi (1.6 M in hexanes, 1.55 mL, 2.48 mmol, 5.00 equiv.) was added dropwise to neat tetravinyltin (280 mg, 0.23 mL, 1.24 mmol, 2.50 equiv.) at room temperature under a N_2 atmosphere. The solution was stirred for 30 min, after which the stirring was stopped, the white solid deposited and the colourless solution was decanted. The solid was washed with pentane (3 x 1 mL), stirring for 5 minutes and decanting the solution after each wash, then it was dissolved in THF (1.5 mL). **15** (308 mg, 0.50 mmol, 1.00 equiv.) was dissolved in Et_2O (5 mL) and cooled to –78 °C. The vinyl lithium solution was added dropwise. The solution was stirred for 30 min at –78 °C, then it was warmed to –42 °C and stirred for 20 min. ^{11}B crude NMR showed complete boronate complex formation [^{11}B NMR (92.6 MHz, no solvent) δ = 7]. The solution was cooled to –78 °C and a solution of iodine (630 mg, 2.48 mmol, 5.00 equiv.) in THF (4 mL) was added dropwise. The solution was stirred for 15 min at –78 °C, then a suspension of MeONa (268 mg, 4.96 mmol, 10.0 equiv.) in MeOH (2 mL) was added. The solution was warmed to room temperature and stirred for 1 h. Aqueous $Na_2S_2O_3$ (10 mL) was added and the layers were separated. The organic layer was washed with Brine (2 x 5 mL) and the combined aqueous layers were extracted with Et_2O (3 x 5 mL). The organic layers were combined, dried over $MgSO_4$ and concentrated under vacuum. The crude product was purified by column chromatography on silica gel, eluting with petrol:EtOAc 97:3 to 95:5, to give **5** as a colourless oil (185 mg, 71%). **[α]$_D$** (20 °C, CHCl3, *c* 1.0) = –47. **FT-IR** ν_{max} (film)/cm^{-1}: 2930, 1690, 1456, 1389, 1105, 700. **^{1}H NMR** (500 MHz, DMSO-*d*$_6$, 90°C): δ = 7.62 (4H, m, H^A), 7.42 (6H, m, H^B & H^{B1}), 5.60 (1H, dt, *J* 10.5, 1.2 Hz, H^C), 5.05 (1H, dd, *J* 10.5, 2.5 Hz, H^D), 4.90 (1H, dd, *J* 17.2, 2.5 Hz, H^E), 3.92 (1H, m, H^F), 3.71 (2H, m, H^G), 3.36 (1H, m, H^H), 3.04 (1H, m, H^I), 2.38 (1H, m, H^J), 1.87-1.56 (6H, m, H^K & H^L & H^M & H^N), 1.39 (9H, s, H^O), 1.23 (1H, m, H^P), 1.02 (9H, s, H^Q), 0.86 (3H d, *J* 6.4 Hz, H^R). **^{13}C NMR** (125 MHz, DMSO-*d*$_6$, 90 °C): δ = 153.9 (C=O), 138.1 (CH), 135.5 (4 x CH), 134.1 (2 x C), 130.1 (2 x CH), 128.1 (4 x CH), 118.0 (CH_2), 78.5 (C), 62.4 (CH_2), 58.7 (CH), 51.8 (CH), 46.9 (CH_2), 37.4 (CH_2), 30.8 (CH), 28.7 (3 x CH_3), 27.3 (3 x CH_3), 26.4 (CH_2), 23.8 (CH_2), 19.2 (C),

17.9 (CH_3). **HRMS** (ESI) Found: $[M+Na]^+$, 544.3209, $[M+H]^+$, 522.3391. $C_{32}H_{47}NaNO_3Si$ requires $[M+Na]^+$, 544.3217, $C_{32}H_{47}NO_3Si$ requires $[M+H]^+$, 522.3398.

*One-pot lithiation-borylation-Zweifel olefination procedure from **6** and **7** to **5**:*

6 (2.52 g, 4.40 mmol, 1.00 equiv.) and (–)-sparteine (1.24 g, 1.20 mL, 5.30 mmol, 1.20 equiv.) were dissolved in Et_2O (45 mL). The mixture was cooled to –78 °C and *s*-BuLi (1.3 M in hexanes, 4.08 mL, 5.30 mmol, 1.20 equiv.) was added. The mixture was stirred for 4 h at –78 °C, then **7** (1.58 g, 5.30 mmol, 1.20 equiv.) in Et_2O (5 mL) was added. The solution was stirred for 45 min at –78 °C, after which crude ^{11}B NMR showed complete boronate complex formation [^{11}B NMR (92.6 MHz, no solvent) $\delta = 7$]. The solution was warmed to room temperature, Et_2O was removed under high vacuum and $CHCl_3$ (45 mL) was added. The solution was heated under reflux (70 °C) for 36 h. Crude ^{11}B NMR showed full product formation [^{11}B NMR (92.6 MHz, no solvent) $\delta = 33$]. $CHCl_3$ was removed under vacuum and Et_2O (45 mL) was added. The mixture was cooled to –78 °C and a freshly prepared solution of vinyl lithium (from tetravinyl tin 2.5 g, 2.0 mL, 11 mmol, 2.5 equiv, and *n*-BuLi, 1.6 M in hexanes, 13.75 mL, 22 mmol, 5.0 equiv.) in THF (20 mL) was added. The solution was stirred for 30 min at –78 °C, then it was warmed to –42 °C and stirred for 20 min. ^{11}B crude NMR showed complete boronate complex formation [^{11}B NMR (92.6 MHz, no solvent) $\delta = 7$]. The solution was cooled to –78 °C and a solution of iodine (5.60 g, 22.0 mmol, 5.00 equiv.) in THF (15 mL) was added dropwise. The solution was stirred for 15 min at –78 °C, then a suspension of MeONa (2.38 mg, 44.0 mmol, 10.0 equiv.) in MeOH (10 mL) was added. The solution was warmed to room temperature and stirred for 1 h. Aqueous $Na_2S_2O_3$ (70 mL) was added and the layers were separated. The organic layer was washed with Brine (2 x 30 mL) and the combined aqueous layers were extracted with Et_2O (3 x 30 mL). The organic layers were combined, dried over $MgSO_4$ and concentrated under vacuum. The crude product was purified by column chromatography on silica gel, eluting with petrol:EtOAc 97:3 to 95:5, to give **5** as a colourless oil (1.6 g, 70%). Data as above.

5. Synthesis of 20

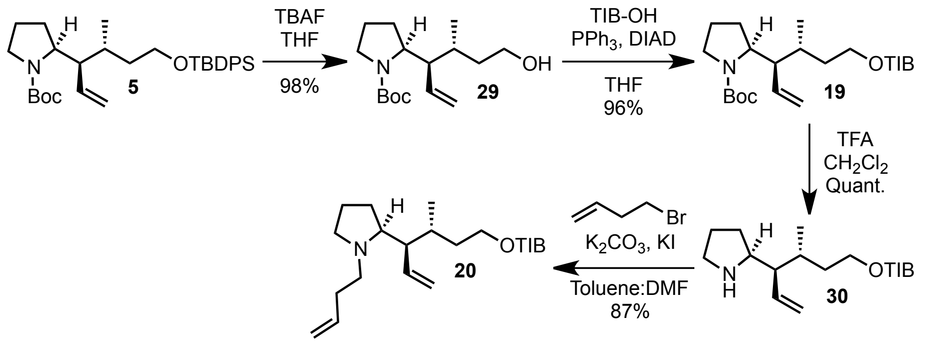

tert-Butyl (S)-2-((3R,4R)-6-hydroxy-4-methylhex-1-en-3-yl)pyrrolidine-1-carboxylate (29)

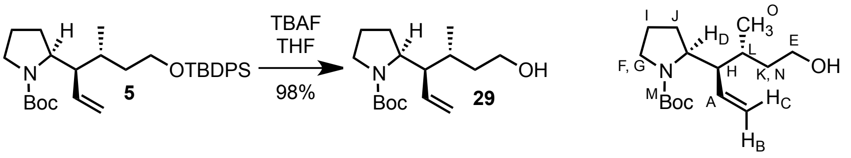

To a solution of **5** (970 mg, 1.86 mmol, 1.00 equiv.) in THF (20 mL) was added tetrabutylammonium fluoride (1.00 M in THF, 3.70 mL, 3.71 mmol, 2.00 equiv.) dropwise at 0 °C. The solution was stirred for 16 h warming to room temperature. Aqueous NH_4Cl (20 mL) was added and the layers were separated. The organic layer was washed with Brine (2 x 10 mL) and the combined aqueous layers were extracted with Et_2O (3 x 10 mL). The organic layers were combined, dried over $MgSO_4$ and concentrated under vacuum. The crude product was purified by column chromatography on silica gel, eluting with petrol: EtOAc 80:20 to 75:25, to give **29** as a colourless oil (525 mg, 98%). **[α]** $[\alpha]_D$ (20 °C, $CHCl_3$, *c* 1.0) = –2. **FT-IR** ν_{max} (film)/cm^{-1}: 3410 (broad), 2925, 1693, 1392, 1168. **^{1}H NMR** (500 MHz, DMSO-d_6, 90 °C): δ = 5.64 (1H, dt, *J* 17.0, 9.7 Hz, H^A), 5.07 (1H, dd, *J* 9.7, 2.0 Hz, H^B), 4.92 (1H, dd, *J* 17.0, 2.0 Hz, H^C), 3.95 (1H, m, H^D), 3.51-3.34 (3H, m, H^E & H^F), 3.05 (1H, m, H^G), 2.39 (1H, m, H^H), 1.90-1.62 (5H, m, H^I & H^J & H^K), 1.57 (1H, m, H^L), 1.42 (9H, s, H^M), 1.11 (1H, m, H^N), 0.91 (3H, d, *J* 7.7 Hz, H^O). **^{13}C NMR** (125 MHz, DMSO-d_6, 90 °C): δ = 154.0 (C=O), 138.4 (CH), 117.8 (CH_2), 78.5 (C), 59.5 (CH_2), 58.7 (CH), 52.1 (CH), 46.9 (CH_2), 37.8 (CH_2), 30.9 (CH), 28.8 (3 x CH_3), 26.5 (CH_2), 23.9 (CH_2), 18.0 (CH_3). **HRMS** (ESI) Found: $[M+Na]^+$, 306.2028, $[M+H]^+$, 284.2211. $C_{16}H_{29}NaNO_3$ requires $[M+Na]^+$, 306.2040, $C_{16}H_{29}NO_3$ requires $[M+H]^+$, 284.2220.

***tert*-Butyl (*S*)-2-((3*R*,4*R*)-4-methyl-6-((2,4,6-triisopropylbenzoyl)oxy)hex-1-en-3-yl)pyrrolidine-1-carboxylate (19)**

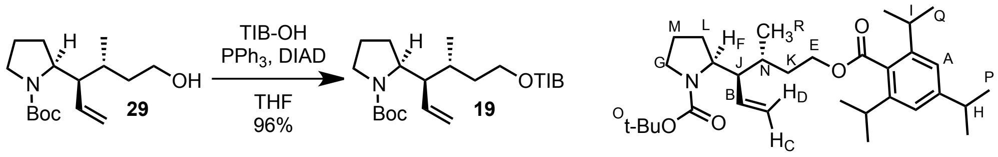

To a solution of **29** (431 mg, 1.52 mmol, 1.10 equiv.) in THF (0.25 M, 6 mL) were added triphenylphosphine (400 mg, 1.52 mmol, 1.10 equiv.) and triisopropylbenzoic acid (343 mg, 1.38 mmol, 1.00 equiv.). The solution was cooled to 0 °C and diisopropylazadicarbozylate (307 mg, 0.30 mL, 1.52 mmol, 1.10 equiv.) was added dropwise. The solution was stirred for 30 min at 0 °C, then it was warmed to room temperature and stirred for another 4 h. Aqueous NH_4Cl (10 mL) was added and the layers were separated. The organic layer was washed with Brine (2 x 5 mL) and the combined aqueous layers were extracted with Et_2O (3 x 10 mL). The organic layers were combined, dried over $MgSO_4$ and concentrated under vacuum. The crude product was purified by column chromatography on silica gel, eluting with petrol:EtOAc 95:5 to 90:10, to give **19** as an oil (685 mg, 96%). **$[\alpha]_D$** (20 °C, $CHCl_3$, *c* 1.0) = –50. **FT-IR** ν_{max} (film)/cm^{-1}: 2962, 1722, 1681,, 1460, 1391, 1250, 753. **^{1}H NMR** (500 MHz, DMSO-d_6, 90°C): δ = 7.05 (2H, s, H^A), 5.65 (1H, dt, *J* 16.8, 9.9 Hz, H^B), 5.09 (1H, dd, *J* 9.9, 1.8 Hz, H^C), 4.96 (1H, dd, *J* 16.8, 1.8 Hz, H^D), 4.30 (2H, m, H^E), 3.96 (1H, m, H^F), 3.38 (1H, m, H^G), 3.06 (1H, m, H^G), 2.90 (1H, sept, *J* 6.7 Hz, H^H), 2.80 (2H, sept, *J* 6.7 Hz, H^I), 2.43 (1H, m, H^J), 1.97 (1H, m, H^K), 1.85 (1H, m, H^L), 1.80-1.57 (4H, m, H^L & H^M & H^N), 1.41 (9H, s, H^O), 1.36 (1H, m, H^K), 1.22 (6H, d, *J* 6.7 Hz, H^P), 1.19 (12 H, d, *J* 6.7 Hz, H^Q), 0.98 (3H, d, *J* 7.0 Hz, H^R). **^{13}C NMR** (125 MHz, DMSO-d_6, 90 °C): δ = 170.3 (C=O), 154.0 (C=O), 150.2 (C), 144.8 (2 x C), 137.8 (CH), 121.0 (2 x CH), 118.2 (CH_2), 78.6 (C), 63.5 (CH_2), 58.5 (CH), 51.6 (CH), 46.9 (CH_2), 34.0 (CH), 33.4 (CH_2), 31.3 (2 x CH), 31.1 (CH), 28.7 (3 x CH_3), 26.6 (CH_2), 24.2 (4 x CH_3), 24.1 (2 x CH_3), 23.8 (CH_2), 17.6 (CH_3). **HRMS** (ESI) Found: $[M+Na]^+$, 536.3710, $[M+H]^+$, 514.3892. $C_{32}H_{51}NaNO_4$ requires $[M+Na]^+$, 336.3710, $C_{32}H_{52}NO_4$ requires $[M+H]^+$, 514.3891.

(3*R*,4*R*)-3-Methyl-4-((*S*)-pyrrolidin-2-yl)hex-5-en-1-yl 2,4,6-triisopropylbenzoate (30)

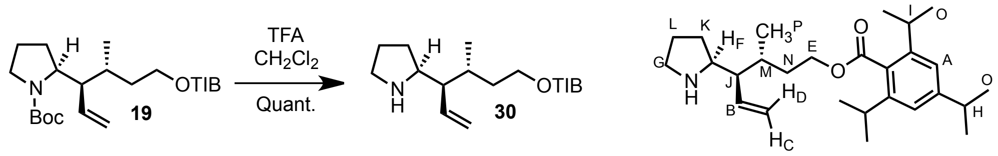

To a solution of **19** (835 mg, 1.62 mmol, 1.00 equiv.) in CH_2Cl_2 (15 mL) was added dropwise TFA (1.24 mL, 16.2 mmol, 10.0 equiv.) at room temperature. The solution was stirred for 90 min, then water (15 mL) was added. The biphasic solution was cooled to 0 °C and NH_4OH was added until the pH of the aqueous layer was basic. The layers were separated and the aqueous layer was extracted with CH_2Cl_2:MeOH:NH_4OH 89.9:10:0.1 (4 x 10 mL). The organic layers were combined, dried over $MgSO_4$ and concentrated under vacuum, to give **30** as an oil, which was used in the following step without further purification (670 mg, quantitative crude yield). **[α]$_D$** (20 °C, $CHCl_3$, *c* 1.0) = –13. **FT-IR** ν_{max} (film)/cm^{-1}: 2960, 1721, 1606, 1460, 1250, 1076, 737. **^{1}H NMR** (400 MHz, $CDCl_3$): δ = 6.98 (2H, s, H^A), 5.53 (1H, dt, *J* 17.0, 10.1 Hz, H^B), 5.15 (1H, dd, *J* 10.1, 2.1 Hz, H^C), 5.09 (1H, dd, *J* 17.0, 2.1 Hz, H^D), 4.38 (1H, ddd, *J* 11.0, 8.0, 5.0 Hz, H^E), 4.27 (1H, dt, *J* 11.0, 7.7 Hz, H^E), 3.16 (1H, m, H^F), 3.01 (1H, dt, *J* 11.1, 6.5 Hz, H^G), 2.93-2.76 (4H, m, H^G & H^H & H^I), 2.03 (1H, m, H^J), 1.97 (1H, m, H^K), 1.81 (1H, m, H^K), 1.76-1.65 (3H, m, H^L & H^M), 1.36 (2H, m, H^N), 1.22 (18H, d, *J* 6.9 Hz, H^O), 0.98 (3H, d, *J* 6.6 Hz, H^P). **^{13}C NMR** (100 MHz, $CDCl_3$): δ = 171.1 (C=O), 150.2 (C), 144.8 (2 x C), 136.1 (CH), 130.7 (C), 120.9 (2 x CH), 119.2 (CH_2), 63.7 (CH_2), 59.5 (CH), 54.6 (CH), 46.4 (CH_2), 34.5 (CH_2), 31.6 (CH), 31.5 (2 x CH), 31.4 (CH_2), 28.6 (CH), 25.3 (CH_2), 24.2 (4 x CH_3), 24.0 (2 x CH_3), 17.9 (CH_3). **HRMS** (ESI) Found: $[M+H]^+$, 414.3385. $C_{27}H_{44}NO_2$ requires $[M+H]^+$, 414.3367.

(3*R*,4*R*)-4-((*S*)-1-(but-3-en-1-yl)pyrrolidin-2-yl)-3-methylhex-5-en-1-yl 2,4,6-triisopropylbenzoate (20)

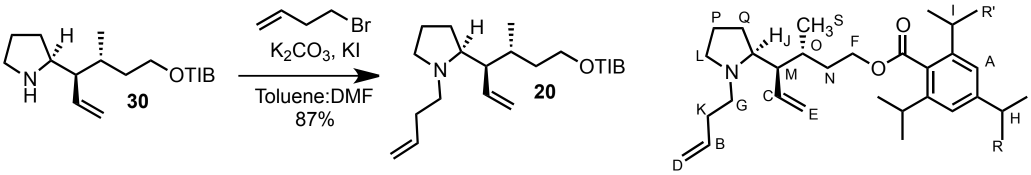

To a solution of **30** (670 mg, 1.62 mmol, 1.00 equiv.) in toluene (0.3 M, 5.5 mL) was added K_2CO_3 (448 mg, 3.24 mmol, 2.00 equiv.). The solution was cooled to 0 °C and 4-bromo-1-butene (0.25 mL, 2.43 mmol, 1.50 equiv.) was added dropwise, followed by addition of KI (54 mg, 0.32 mmol, 0.20 equiv.) and DMF (0.2 mL). The solution was heated to reflux and stirred for 72 h. Water (5 mL) was added and the layers were separated. The organic layer was washed with Brine (2 x 5 mL) and the combined aqueous layers were extracted with EtOAc (3 x 5 mL). The organic layers were combined, dried over $MgSO_4$ and concentrated under vacuum. The crude product was purified by column chromatography on silica gel, eluting with CH_2Cl_2:MeOH:NH_4OH 99.5:0:0.5 to 96.5:3:0.5, to give **20** as an oil (663 mg, 87%). **[α]$_D$** (20 °C, $CHCl_3$, *c* 1.0) = –47. **FT-IR** ν_{max} (film)/cm^{-1}: 2960, 1723, 1460, 1250, 1074, 909, 756. **^{1}H NMR** (500 MHz, $CDCl_3$): δ = 7.02 (2H, s, H^A), 5.80 (1H, m, H^B), 5.68 (1H, dt, *J* 17.0, 9.9 Hz, H^C), 5.18-4.97 (4H, m, H^D & H^E), 4.41 (1H, ddd, *J* 11.0, 7.9, 5.0 Hz, H^F), 4.31 (1H, dt, *J* 11.0, 7.6 Hz, H^F), 3.19 (1H, m, H^G), 2.95-2.79 (4H, m, H^G & H^H & H^I), 2.47 (1H, m, H^J), 2.24 (2H, m, H^K), 2.14-2.00 (4H, m, H^L & H^M & H^N), 1.77-1.64 (5H, m, H^O & H^P & H^Q), 1.40 (1H, m, H^N), 1.26 (6H, d, *J* 6.7 Hz, H^R), 1.25 (12H, d, *J* 6.7 Hz, $H^{R'}$), 1.02 (3H, d, *J* 6.8 Hz, H^S). **^{13}C NMR** (125 MHz, $CDCl_3$): δ = 171.0 (C=O), 150.1 (C), 144.7 (2 x C), 137.1 (CH), 136.8 (CH), 130.7 (C), 120.8 (2 x CH), 117.2 (CH_2), 115.3 (CH_2), 65.2 (CH), 63.3 (CH_2), 53.8 (CH_2), 53.7 (CH_2), 51.3 (CH), 34.4 (CH), 33.3 (CH_2), 32.9 (CH_2), 31.5 (2 x CH), 30.9 (CH), 25.5 (CH_2), 24.2 (4 x CH_3), 24.1 (2 x CH_3), 22.2 (CH_2), 17.6 (CH_3). **HRMS** (ESI) Found: $[M+H]^+$, 468.3829. $C_{31}H_{50}NO_2$ requires $[M+H]^+$, 468.3836.

6. Synthesis of 3

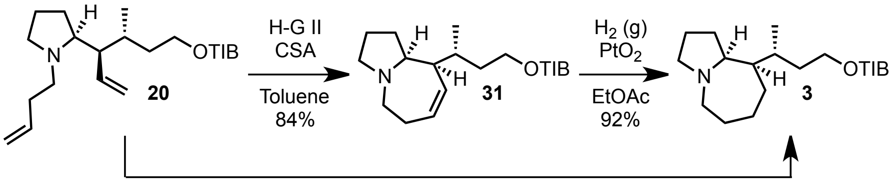

(*R*)-3-((9*S*,9a*S*)-2,3,5,6,9,9*a*-Hexahydro-1*H*-pyrrolo[1,2-*a*]azepin-9-yl)butyl 2,4,6-triisopropylbenzoate (31)

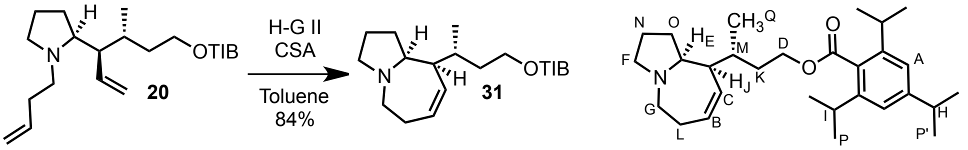

20 (350 mg, 0.75 mmol, 1.00 equiv.) was dissolved in toluene (0.01 M, 75 mL). The solution was degassed, then camphorsulfonic acid (184 mg, 0.79 mmol, 1.05 equiv.) was added. The solution was stirred for 20 min, then Hoveyda-Grubbs 2nd Generation catalyst (94 mg, 0.15 mmol, 0.20 equiv.) was added. The solution was heated to 80 °C and stirred at that temperature for 5 h. Ethyl vinyl ether (0.2 mL) was added, followed by water (50 mL) and NH_4OH, until the aqueous layer reached basic pH. The layers were separated, the organic layer was washed with Brine (2 x 20 mL) and the combined aqueous layers were extracted with EtOAc (3 x 30 mL). The organic layers were combined, dried over $MgSO_4$ and concentrated under vacuum. The crude product was purified by column chromatography on silica gel, eluting with CH_2Cl_2:MeOH:NH_4OH 99.5:0:0.5 to 94.5:5:0.5, to give **31** as an oil (275 mg, 84%). **[α]$_D$** (20 °C, $CHCl_3$, *c* 1.0) = +42. **FT-IR** ν_{max} (film)/cm^{-1}: 2960, 1721, 1460, 1250, 1075, 908, 731. **^{1}H NMR** (500 MHz, $CDCl_3$): δ = 7.03 (2H, s, H^A), 5.72 (1H, ddd, *J* 11.3, 5.8, 4.3 Hz, H^B), 5.64 (1H, dd, *J* 11.3, 6.4, H^C), 4.41 (1H, ddd, *J* 11.1, 7.9, 5.1 Hz, H^D), 4.31 (1H, dt, *J* 11.1, 7.6 Hz, H^D), 3.10-2.97 (3H, m, H^E & H^F & H^G), 2.91 (1H, sept, *J* 7.0 Hz, H^H), 2.87 (2H, sept, *J* 7.0 Hz, H^I), 2.74-2.59 (2H, m, H^F & H^G), 2.46 (1H, m, H^J), 2.43-2.34 (2H, m, H^K & H^L), 2.29 (1H, m, H^L), 1.96-1.88 (2H, m, H^M & H^N), 1.83-1.71 (3H, m, H^N & H^O), 1.45 (1H, m, H^K), 1.26 (12H, d, *J* 7.0 Hz, H^P), 1.25 (6H, d, *J* 7.0 Hz, $H^{P'}$), 1.05 (3H, d, *J* 6.7 Hz, H^Q). **^{13}C NMR** (125 MHz, $CDCl_3$): δ = 171.0 (C=O), 150.0 (C), 144.7 (2 x C), 132.2 (CH), 130.7 (C), 129.4 (CH), 120.8 (2 x CH), 66.9 (CH), 63.8 (CH_2), 55.2 (CH_2), 51.0 (CH_2), 46.3 (CH), 34.4 (CH), 33.2 (CH_2), 31.7 (CH), 31.5 (2 x CH), 28.5 (CH_2), 28.1 (CH_2), 24.2 (4 x CH_3), 24.0 (2 x CH_3), 23.3 (CH_2), 18.6 (CH_3). **HRMS** (ESI)

Found: $[M+H]^+$, 440.3524. $C_{29}H_{46}NO_2$ requires $[M+H]^+$, 440.3523.

(*R*)-3-((9*R*,9a*S*)-Octahydro-1*H*-pyrrolo[1,2-*a*]azepin-9-yl)butyl 2,4,6-triisopropylbenzoate (3)

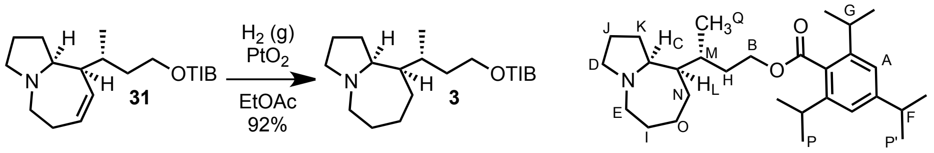

To a solution of **31** (373 mg, 0.85 mmol, 1.00 equiv.) in EtOAc (0.1 M, 8.5 mL) was added PtO_2 (19.3 mg, 0.085 mmol, 0.10 equiv.) under a N_2 atmosphere. The solution was purged with H_2 (g) for 30 min, after which it was stirred under a H_2 atmosphere for 24 h. The solution was filtered through celite, washing copiously with EtOAc, then the solvent was removed under vacuum. The crude product was purified by column chromatography on silica gel, eluting with CH_2Cl_2:MeOH:NH_4OH 96.9:3:0.1, to give **3** as an oil (334 mg, 90%). **$[\alpha]_D$** (20 °C, $CHCl_3$, *c* 1.0) = –10. **FT-IR** ν_{max} (film)/cm^{-1}: 2962, 1718, 1264, 1076, 733. **^{1}H NMR** (500 MHz, $CDCl_3$): δ = 7.02 (2H, s, H^A), 4.40 (1H, ddd, *J* 10.8, 7.8, 5.3 Hz, H^B), 4.32 (1H, dt, *J* 10.8, 7.8 Hz, H^B), 3.18 (1H, br m, H^C), 3.06-2.99 (2H, m, H^D & H^E), 2.90 (1H, sept, *J* 7.0 Hz, H^F), 2.86 (2H, sept, *J* 7.0 Hz, H^G), 2.78-2.64 (2H, m, H^D & H^E), 2.16 (1H, m, H^H), 1.86-1.72 (4H, m, H^I & H^J), 1.70-1.60 (3H, m, H^K & H^L & H^M), 1.55 (1H, m, H^N), 1.52-1.41 (2H, m, H^H & H^N), 1.32-1.19 (21H, m, H^K & H^O & H^P & $H^{P'}$), 0.97 (3H, d, *J* 6.5 Hz, H^Q). **^{13}C NMR** (125 MHz, $CDCl_3$): δ = 171.0 (C=O), 150.1 (C), 144.7 (2 x C), 130.7 (C), 120.9 (2 x CH), 65.3 (CH), 63.7 (CH_2), 53.9 (CH_2), 51.8 (CH_2), 46.0 (CH), 34.4 (CH), 32.6 (CH_2), 32.5 (CH), 31.5 (2 x CH), 29.7 (CH_2), 28.0 (CH_2), 27.7 (CH_2), 25.8 (CH_2), 24.2 (4 x CH_3), 23.9 (2 x CH_3), 23.7 (CH_2), 18.3 (CH_3). **HRMS** (ESI) Found: $[M+H]^+$, 442.3678. $C_{29}H_{48}NO_2$ requires $[M+H]^+$, 442.3680.

*One-pot RCM-hydrogenation procedure from **17** to **3**:*

17 (200 mg, 0.43 mmol, 1.00 equiv.) was dissolved in toluene (0.01 M, 45 mL). The solution was degassed, then camphorsulfonic acid (105 mg, 0.45 mmol, 1.05 equiv.) was added. The solution was stirred for 30 min, then Hoveyda-Grubbs 2nd Generation catalyst (53 mg, 0.085 mmol, 0.20 equiv.) was added. The solution was stirred at 80 °C for 5 h, then it was cooled to room temperature. PtO_2 (10 mg, 0.043 mmol, 0.1 equiv.) was added under a N_2 atmosphere. The solution was purged with H_2 (g) for 30 min, after which it was stirred under a H_2 atmosphere for 24 h. The solution was filtered

through celite, washing copiously with EtOAc, then the solvent was removed under vacuum. The crude product was purified by column chromatography on silica gel, eluting with CH_2Cl_2:MeOH:NH_4OH 96.9:3:0.1, to give **3** as an oil (155 mg, 82%).

7. Optimisation of the Asymmetric Lithiation of 3

7.1. Initial *in situ* React-IR Monitoring

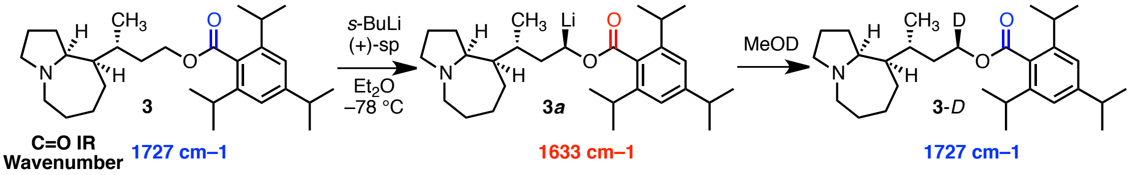

To a three-necked round-bottomed flask equipped with a ReactIR probe was added a solution of TIB ester **3** (140 mg, 0.32 mmol, 1.00 equiv.) and (+)-sparteine (73 mg, 0.07 mL, 0.32 mmol, 1.00 equiv.) in Et_2O (1.5 mL). A peak at 1727 cm^{-1} was observed, which was assigned to the C=O bond stretch in **3**. The solution was cooled to –78 °C and *s*-BuLi (1.3 M in hexanes, 0.24 mL, 0.32 mmol, 1.00 equiv.) was added dropwise. After 1 h no change in the IR signals was observed, which indicated that the lithiation to give **3*a*** was not taking place. Upon addition of a second equivalent of *s*-BuLi, the peak at 1727 cm^{-1} started to decrease slowly, accompanied by the appearance of a peak at 1633 cm^{-1}, characteristic of the C=O bond stretch of lithiated TIB ester **3*a***. The reaction mixture was stirred for another 3 h, observing a very slow conversion, and after another 2 h of no evolution the reaction was quenched with MeOD (0.1 mL). The reaction mixture was diluted with EtOAc (5 mL), water was added (5 mL) and the layers were separated. The organic layer was washed with Brine (2 x 5 mL) and the combined aqueous layers were extracted with EtOAc (3 x 5 mL). The organic layers were combined, dried over $MgSO_4$ and concentrated under vacuum. After purification by column chromatography on silica gel, eluting with CH_2Cl_2:MeOH:NH_4OH 99.9:0:0.1 to 96.9:3:0.1, TIB ester **3** was recovered (110 mg, 79%) with <5% D incorporation observed by ^{1}H NMR.

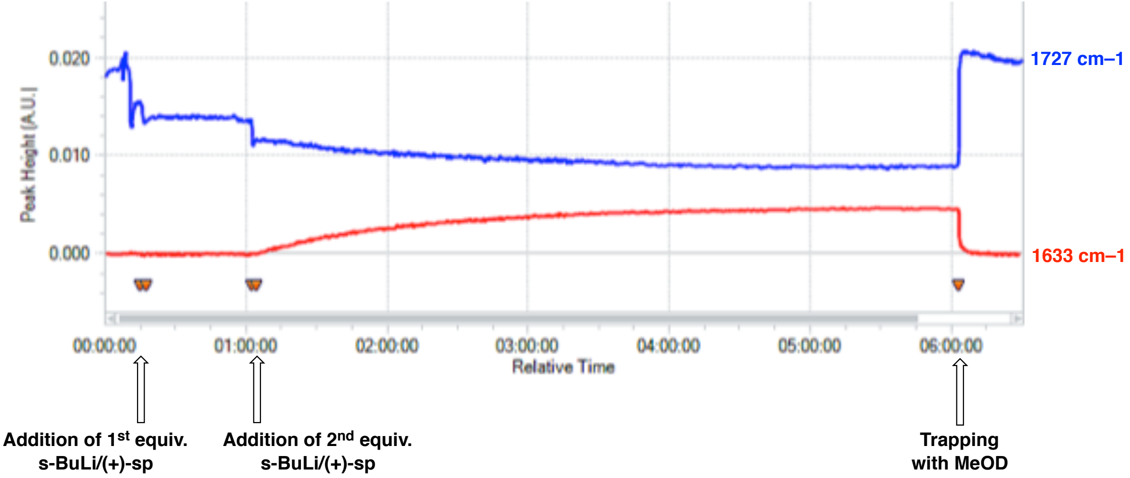

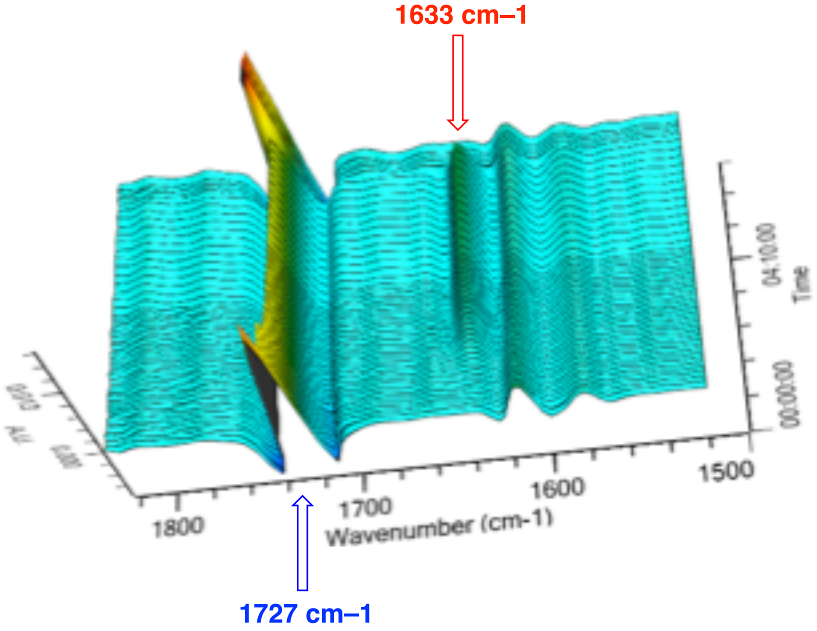

1633 cm–1
1727 cm–1
1800
1700
1600
1500
Wavenumber (cm-1)
Time

7.2. Optimization of the Asymmetric Lithiation of 3 Using (+)-Sparteine Surrogate

General Procedure for the Asymmetric Lithiation of 3 – GP3

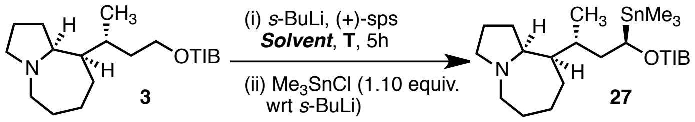

3 (20 mg, 0.045 mmol, 1.0 equiv.) and (+)-sparteine surrogate (see Table 2 for equiv.) were dissolvent in the appropriate solvent (1 mL). The solution was cooled to the appropriate temperature and *s*-BuLi (1.3 M in hexanes, see Table 2 for equiv.) was added dropwise). The mixture was stirred for 5 h at the appropriate temperature, then Me_3SnCl (1.0 M in hexane, 1.1 equiv. with respect to *s*-BuLi/(+)-sps) was added dropwise. The reaction mixture was stirred at –78 °C for 1 h then slowly warmed to room temperature. Aqueous KF (5 mL) was added and the layers were separated. The organic layer was washed with Brine (2 x 5 mL) and the combined aqueous layers were extracted with EtOAc (3 x 5 mL). The organic layers were combined, dried over $MgSO_4$ and concentrated under vacuum. The ratio between **3** and stannane **27** was determined by ^{1}H NMR analysis of the crude mixture.

Entry	Solvent	*s*-BuLi•(+)-sps (equiv.)	T (°C)	**3**:**27**	Yield (%)
1	Et_2O	2.0	–78	100:0	-
2	Toluene	2.0	–78	100:0	-
3	TBME	2.0	–78	49:51	-
4	CPME	2.0	–78	35:65	-
5	CPME	2.0	–63	0:100	55
6	CPME	2.5	–78	0:100	67
7	CPME	3.0[a]	–78	65:35	-
8	CPME	3.0	–78	0:100	84
9	CPME	3.0	–78	0:100	92[b]

[a] Reaction carried out with (+)-sparteine instead of (+)-sparteine surrogate
[b] Reaction carried out in a 230 mg (0.50 mmol) scale under optimised conditions (1 h lithiation - *vide infra* for ReactIR analysis)

7.3. *in situ* React-IR monitoring

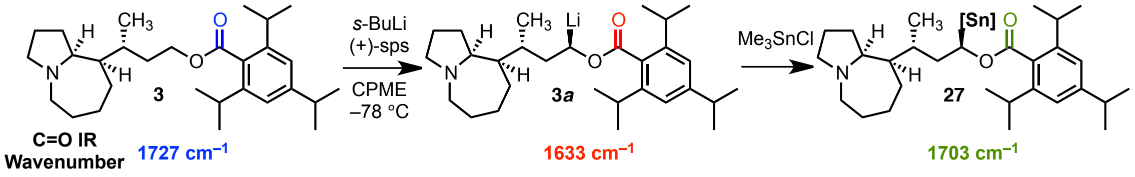

To a three-necked round-bottomed flask equipped with a ReactIR probe was added a solution of TIB ester **3** (100 mg, 0.23 mmol, 1.00 equiv.) and (+)-sparteine surrogate (132 mg, 0.13 mL, 0.68 mmol, 3.00 equiv.) in CPME (3.0 mL). A peak at 1727 cm^{-1} was observed, which was assigned to the C=O bond stretch in **3**. The solution was cooled to –78 °C and *s*-BuLi (1.3 M in hexanes, 0.52 mL, 0.68 mmol, 3.00 equiv.) was added dropwise. This led to the gradual disappearance of the signal at 1727 cm^{-1} and the appearance of a new signal at 1633 cm^{-1}, characteristic of the C=O bond stretch of lithiated TIB ester **3*a***. This signal rose to a plateau over a period of 20 min following completion of *s*-BuLi addition. After another 20 min, Me_3SnCl (1.0 M in hexanes, 0.76 mL, 0.76 mmol, 3.30 equiv.) was added dropwise. This led to the almost immediate disappearance of the signal at 1633 cm^{-1} and the simultaneous appearance of a third signal at 1703 cm^{-1}, characteristic of the C=O bond stretch of stannane **27**. The reaction mixture was stirred at –78 °C for 1 h then slowly warmed to room temperature. Aqueous KF (3 mL) was added and the layers were separated. The organic layer was washed with Brine (2 x 5 mL) and the combined aqueous layers were extracted with EtOAc (3 x 5 mL). The organic layers were combined, dried over $MgSO_4$ and concentrated under vacuum. ^{1}H NMR analysis of the crude product showed no presence of TIB ester **7**, indicating that its lithiation was complete. The crude product was purified by column chromatography on silica gel, eluting with CH_2Cl_2:MeOH:NH_4OH 99.9:0:0.1 to 96.9:3:0.1, to give **27** as an oil (105 mg, 76%).

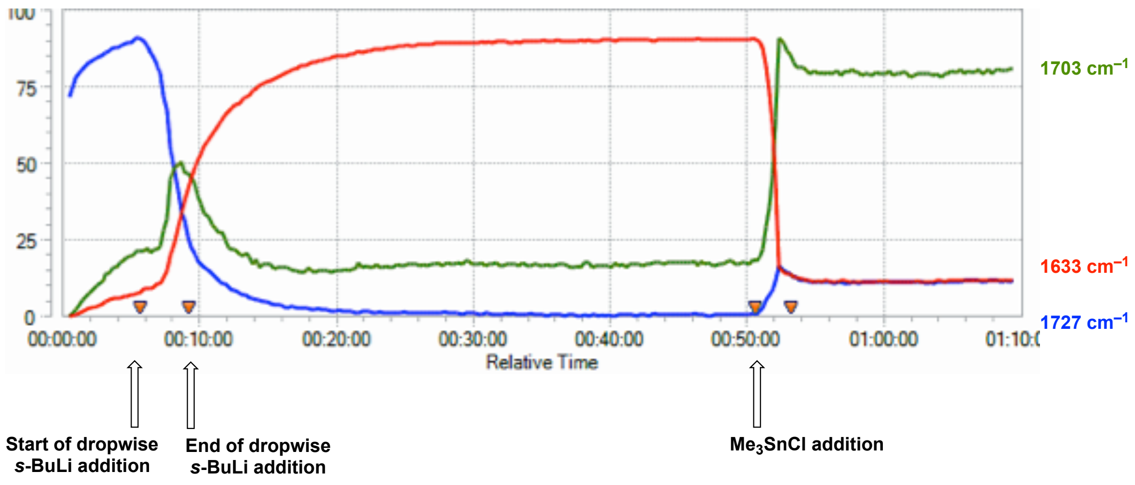

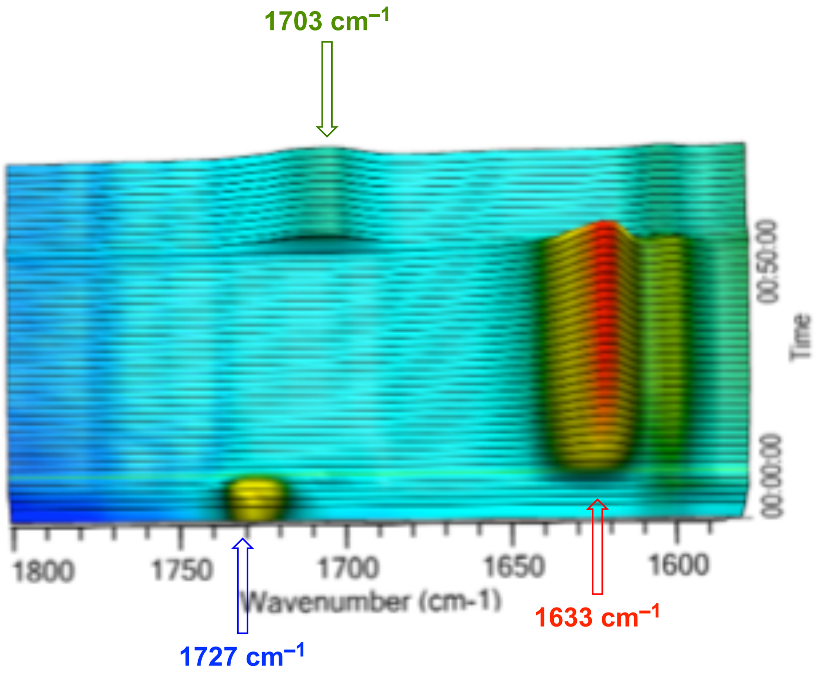

(1*R*,3*R*)-3-((9*R*,9a*S*)-octahydro-1*H*-pyrrolo[1,2-*a*]azepin-9-yl)-1-(trimethylstannyl)butyl 2,4,6-triisopropylbenzoate (27)

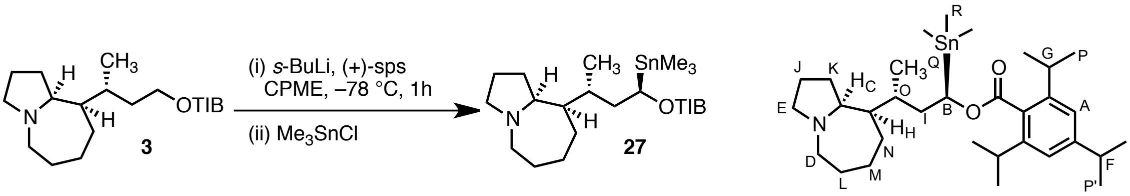

A solution of **3** (230 mg, 0.52 mmol, 1.00 equiv.) and (+)-sparteine surrogate (303 mg, 0.30 mL, 1.56 mmol, 3.00 equiv.) in CPME (0.1 M, 5.0 mL) was cooled to –78 °C and *s*-BuLi (1.3 M in hexanes, 1.20 mL, 1.56 mmol, 3.00 equiv.) was added dropwise. The reaction mixture was stirred at –78 °C for 1 h, then Me_3SnCl (1.0 M in hexanes, 1.72 mL, 1.72 mmol, 3.30 equiv.) was added dropwise. The reaction mixture was stirred at –78 °C for 1 h then slowly warmed to room temperature. Aqueous KF (5 mL) was added and the layers were separated. The organic layer was washed with Brine (2 x 5 mL) and the combined aqueous layers were extracted with EtOAc (3 x 5 mL). The organic layers were combined, dried over $MgSO_4$ and concentrated under vacuum. The crude product was purified by column chromatography on silica gel, eluting with CH_2Cl_2:MeOH:NH_4OH 99.9:0:0.1 to 96.9:3:0.1, to give **27** as an oil (288 mg, 92%, 94:6 d.r. as seen by ^{1}H NMR). **[α]D** (20 °C, $CHCl_3$, *c* 1.0) = –29. **FT-IR** ν_{max} (film)/cm^{-1}: 2960, 1703, 1460, 1250, 876, 767. **^{1}H NMR** (500 MHz, $CDCl_3$): δ = 7.01 (2H, s, 2 x CH^A), 5.20 (1H, dd, *J* 9.5, 6.2 Hz, CH^B), 3.89 (1H, m, CH^C), 3.47 (1H, m, CH_2^{D}), 3.24

(2H, m, CH_2^E), 3.02 (1H, m, CH_2^D), 2.89 (1H, sept, *J* 6.9 Hz, CH^F), 2.82 (2H, sept, *J* 6.9 Hz, 2 x CH^G), 2.15-1.34 (14H, m, CH^H & CH_2^I & CH_2^J & CH_2^K & CH_2^L & CH_2^M & CH_2^N & CH^O), 1.25 (12H, d, *J* 6.9 Hz, 4 x CH_3^P), 1.24 (6H, d, *J* 6.9 Hz, 2 x $CH_3^{P'}$), 0.99 (3H, d, *J* 7.0 Hz, CH_3^Q), 0.24 (9H, s and d, *J* 53.0 Hz, 3 x CH_3^R). **^{13}C NMR** (125 MHz, $CDCl_3$): δ = 171.3 (C=O), 150.0 (C), 144.8 (2 x C), 130.7 (C), 120.8 (2 x CH), 71.3 (CH), 64.6 (CH), 53.4 (CH_2), 50.7 (CH_2), 44.9 (CH), 38.2 (CH_2), 35.3 (CH), 34.4 (CH), 31.5 (2 x CH), 28.5 (CH_2), 27.7 (CH_2), 26.5 (CH_2), 24.4 (2 x CH_3), 24.3 (4 x CH_3), 23.9 (CH_2), 23.4 (CH_2), 18.8 (CH_3), –9.0 (CH_3, s and d, *J* 330.6 Hz, and d, *J* 316.2 Hz) **HRMS** (ESI) Found: $[M+H]^+$, 442.3678. $C_{29}H_{48}NO_2$ requires $[M+H]^+$, 442.3680.

8. Synthesis of Building Block 4

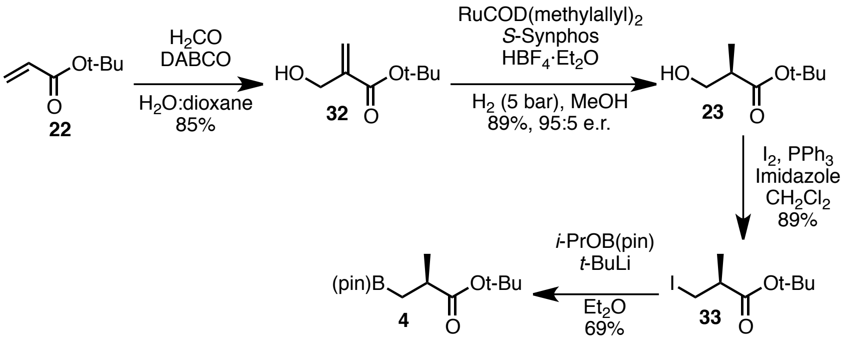

tert-Butyl 2-(hydroxymethyl)acrylate (32)

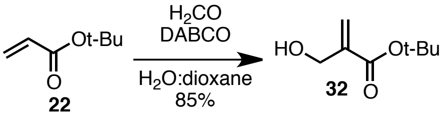

Following a literature procedure,[13] *tert*-butyl acrylate (**22**) (1.00 g, 1.14 mL, 7.80 mmol, 1.00 equiv.) was dissolved in H_2O:dioxane (50 mL, 0.15 M). DABCO (2.62 g, 23.4 mmol, 3.00 equiv.) and formaldehyde (37% w/w solution in H_2O, 1.90 mL, 23.4 mmol, 3.00 equiv.) were added and the solution was stirred at room temperature for 48 h. Et_2O (30 mL) was added and the layers were separated. The organic layer was washed with Brine (2 x 20 mL) and the combined aqueous layers were extracted with Et_2O (3 x 20 mL). The organic layers were combined, dried over $MgSO_4$ and concentrated under vacuum. The crude product was purified by column chromatography on silica gel, eluting with petrol:EtOAc 3:1, to give **32** as a colourless oil (1.04 g, 85%). **FT-IR** ν_{max} (film)/cm^{-1}: 3418 (broad), 2978, 1705, 1368, 1147, 1051, 847. **^{1}H NMR** (400 MHz, $CDCl_3$): δ = 6.14 (1H, m), 5.73 (1H, dt, J 2.8, 1.3 Hz), 4.27 (2H, br m), 2.34 (1H, br s), 1.49 (9H, s). **^{13}C NMR** (100 MHz, $CDCl_3$): δ = 165.7 (C=O), 140.8 (C), 124.8 (CH_2), 81.4 (C), 62.8 (CH_2), 28.1 (3 x CH_3). LRMS (ESI) Found [M+Na]$^+$, 181.0840. Data in accordance with the literature.[13]

tert-butyl 3-hydroxy-2-methylpropanoate (23)

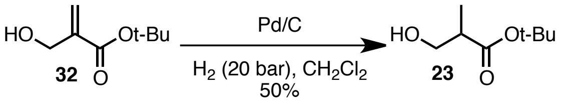

32 (100 mg, 0.63 mmol, 1.0 equiv.) was dissolved in CH_2Cl_2 (3 mL). 5% Palladium on charcoal (116 mg, 0.10 equiv. Pd) was added. The solution was placed under H_2 atmosphere (20 bar) for 2 h. The Pd salts were filtered through celite, washing with

CH_2Cl_2, to give crude *rac*-**23** as an oil (50 mg, 50%), which was used in the next step without further purification. **FT-IR** ν_{max} (film)/cm^{-1}: 3442 (broad), 2977, 1724, 1367, 1152, 1030, 846. **^{1}H NMR** (400 MHz, $CDCl_3$): δ = 3.67 (1H, dd, *J* 11.2, 7.0 Hz), 3.63 (1H, dd, *J* 11.2, 5.0 Hz), 2.54 (1H, ap. pd, *J* 7.1, 5.0 Hz), 1.45 (9H, s), 1.12 (3H, d, *J* 7 Hz). **^{13}C NMR** (100 MHz, $CDCl_3$): δ = 175.3 (C=O), 81.0 (C), 64.8 (CH_2), 42.5 (CH), 28.2 (3 x CH_3), 13.6 (CH_3). LRMS (ESI) Found $[M+Na]^+$, 183.0992. Data in accordance with the literature.[13]

***tert*-Butyl (*R*)-3-hydroxy-2-methylpropanoate [(*R*)-23]**

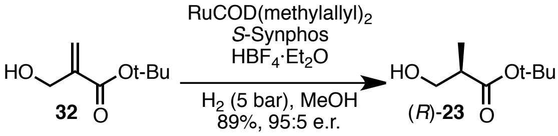

Following a literature procedure,[13] Ru(COD)(methylallyl)$_2$ (10 mg, 0.02 mmol, 0.01 equiv.) and *(S)*-Synphos (21 mg, 0.033 mmol, 0.011 equiv.) were added to a Schlenck tube, under N_2 atmosphere, and dissolved in degassed CH_2Cl_2 (3 mL). The solution was cooled to 0 °C and $HBF_4\cdot Et_2O$ (0.17 M in degassed CH_2Cl_2, 0.05 mL solution, 0.066 mmol, 0.022 equiv.) was added dropwise. The solution was stirred for 30 min at room temperature, at which time an orange suspension appeared. The solvent was removed under vacuum, and a solution of **32** (475 mg, 3.00 mmol, 1.00 equiv.) in degassed MeOH (9 mL) was added. The solution was placed under H_2 atmosphere (5 bar) and stirred at room temperature for 24 h. After evaporation of the solvent, the crude product was purified by column chromatography on silica gel, eluting with petrol:EtOAc 3:1, to give **23** as a colourless oil (427 mg, 89%, 95: e.r. – determined after derivatisation to **23*a***, *vide infra*). **$[\alpha]_D$** (20 °C, $CHCl_3$, *c* 1.0) = –12. Other data as above. Data in accordance with the literature.[13]

3-(*tert*-butoxy)-2-methyl-3-oxopropyl benzoate (23*a*)

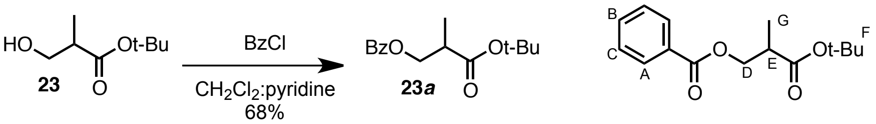

Following a modified literature procedure[14], a solution of **23** (72 mg, 0.45 mmol, 1.00 equiv.) in CH_2Cl_2:pyridine 2:1 (0.5 M, 0.3 mL) was cooled to 0 °C and benzoyl chloride (100 mg, 0.084 mL, 0.72 mmol, 1.50 equiv.) was added dropwise. The solution was warmed to room temperature and stirred for 6 h. Aqueous NH_4Cl (0.5 mL) was

added and the layers were separated. The organic layer was washed with Brine (2 x 1 mL) and the combined aqueous layers were extracted with CH_2Cl_2 (3 x 2 mL). The organic layers were combined, dried over $MgSO_4$ and concentrated under vacuum. The crude product was purified by column chromatography on silica gel, eluting with petrol:EtOAc 99:1, to give **23*a*** as an oil (80 mg, 68%). **FT-IR** ν_{max} (film)/cm^{-1}: 2977, 1722, 1270, 710. **^{1}H NMR** (400 MHz, $CDCl_3$): δ = 8.03 (2H, d, *J* 7.4 Hz, H^A), 7.56 (1H, t, *J* 7.4 Hz, H^B), 7.35 (2H, t, *J* 7.4 Hz, H^C), 4.44 (1H, dd, *J* 11.0, 7.3 Hz, H^D), 4.39 (1H, dd, *J* 11.0, 5.9 Hz, H^D), 2.84 (1H, ap. sext., *J* 6.8 Hz, H^E), 1.44 (9H, s, H^F), 1.25 (3H, d, *J* 6.8 Hz, H^G). **^{13}C NMR** (100 MHz, $CDCl_3$): δ =173.0 (C=O), 166.2 (C=O), 132.9 (CH), 130.1 (C), 129.6 (2 x CH), 128.3 (2 x CH), 80.8 (C), 66.4 (CH_2), 40.2(CH), 28.0 (3 x CH_3), 13.8 (CH_3). **HRMS** (ESI) Found: $[M+Na]^+$, 287.1259. $C_{15}H_{20}NaO_4$ requires $[M+Na]^+$, 287.1254.

Resolution of the enantiomers of 23 was achieved using chiral HPLC analysis (IA column, hexane:IPA 99:1 (10% of 10% IPA in hexane), 1 mL/min, 273 nm, t_R 8.020 min (*S*), 9.058 (*R*).

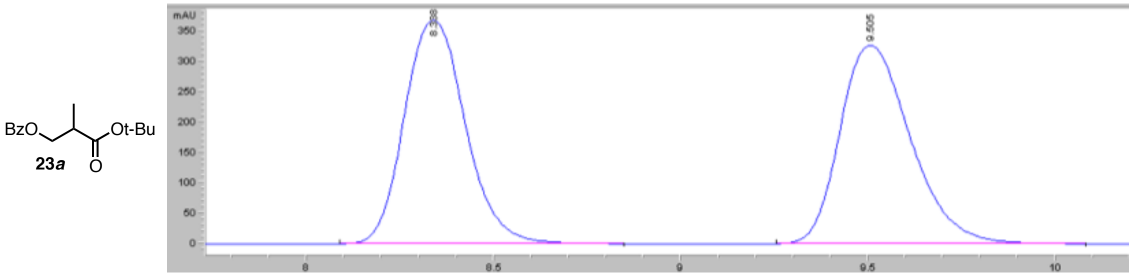

(*R*)-3-(*tert*-Butoxy)-2-methyl-3-oxopropyl benzoate [(*R*)-23*a*]

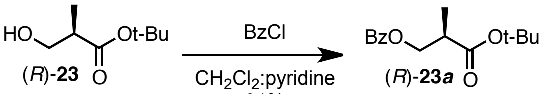

Following a modified literature procedure[14], a solution of (*R*)-**23** (30 mg, 0.20 mmol, 1.00 equiv.) in CH_2Cl_2:pyridine 2:1 (0.5 M, 0.3 mL) was cooled to 0 °C and benzoyl chloride (40 mg, 0.033 mL, 0.30 mmol, 1.50 equiv.) was added dropwise. The solution was warmed to room temperature and stirred for 6 h. Aqueous NH_4Cl (0.5 mL) was added and the layers were separated. The organic layer was washed with Brine (2 x 1 mL) and the combined aqueous layers were extracted with CH_2Cl_2 (3 x 2 mL). The organic layers were combined, dried over $MgSO_4$ and concentrated under vacuum. The crude product was purified by column chromatography on silica gel, eluting with

petrol:EtOAc 99:1, to give (*R*)-**23*a*** as an oil (40 mg, 81%, 95:5 e.r.). **[α]D** (20 °C, $CHCl_3$, *c* 1.0) = –9. Other data as above.

Chrial HPLC analysis (IA column, hexane:IPA 99:1 (10% of 10% IPA in hexane), 1 mL/min, 273 nm, t_R 8.020 min [(*S*), minor], 9.058 [(*R*), major]:

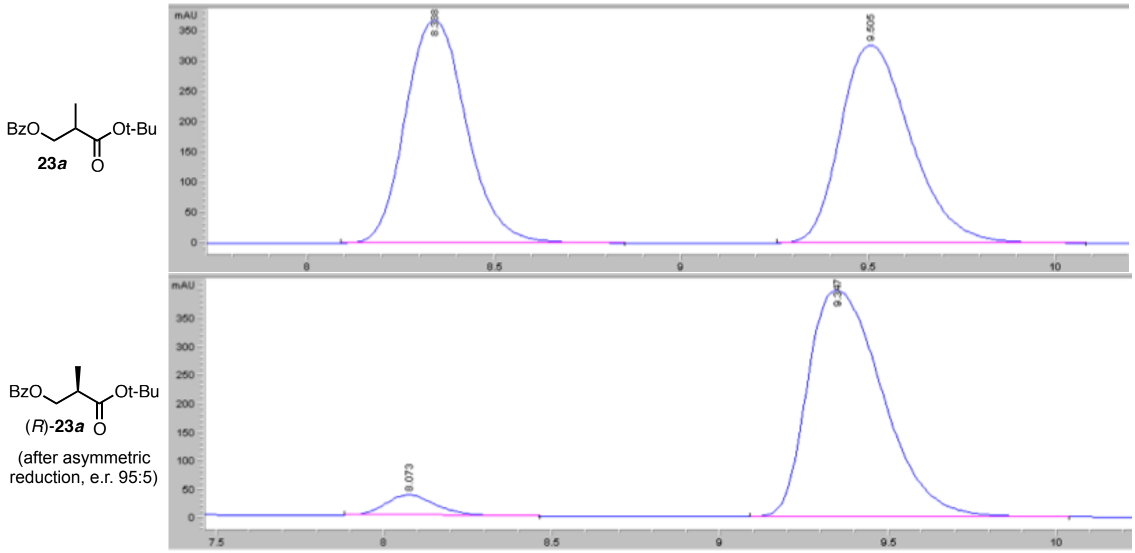

***tert*-Butyl (*S*)-3-iodo-2-methylpropanoate (33)**

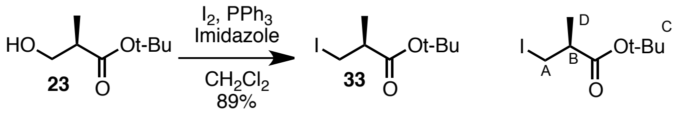

Following a modified literature procedure,[15] **23** (86 mg, 0.54 mmol, 1.00 equiv.) was dissolved in CH_2Cl_2 (6 mL). The flask was protected from light and triphenylphosphine (247 mg, 0.94 mmol, 1.50 equiv.), imidazole (73 mg, 1.08 mmol, 2.00 equiv.) and iodine (238 mg, 0.94 mmol, 1.50 equiv.) were added. The solution was stirred for 4 h at room temperature, then saturated aqueous $Na_2S_2O_3$ (5 mL) was added and the layers were separated. The organic layer was washed with Brine (2 x 5 mL) and the combined aqueous layers were extracted with CH_2Cl_2 (3 x 5 mL). The organic layers were combined, dried over $MgSO_4$ and concentrated under vacuum. The crude product was purified by column chromatography on silica gel, eluting with petrol:Et_2O 95:5, to give **33** as a colourless oil (130mg, 89%). **[α]D** (20 °C, $CHCl_3$, *c* 1.0) = –12. **FT-IR** ν_{max} (film)/cm^{-1}: 2976, 1727, 1457, 1367, 1144, 846. **^{1}H NMR** (400 MHz, $CDCl_3$): δ = 3.33 (1H, dd, *J* 9.6, 6.5 Hz, H^A), 3.22 (1H, dd, *J* 9.6, 6.5 Hz, H^A), 2.65 (1H, ap. sext., *J* 6.5 Hz, H^B), 1.45 (9H, s, H^C), 1.22 (3H, d, *J* 6.5 Hz, H^D). **^{13}C NMR** (100 MHz, $CDCl_3$): δ = 170.9 (C=O), 79.5 (C), 41.3 (CH), 26.3 (3 x CH_3), 16.4 (CH_3), 6.1 (CH_2). **HRMS** (ESI) could not be obtained.

***tert*-Butyl (*R*)-2-methyl-3-(4,4,5,5-tetramethyl-1,3,2-dioxaborolan-2-yl)propanoate (4)**

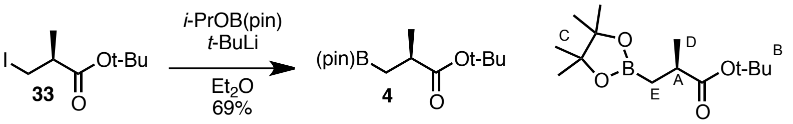

To a solution of **33** (110 mg, 0.41 mmol, 1.00 equiv.) in Et_2O (4 mL) was added *i*-PrOB(pin) (91 mg, 0.10 mL, 0.49 mmol, 1.20 equiv.). The solution was cooled to −105 °C and *t*-BuLi (1.6 M in pentane, 0.51 mL, 0.82 mmol, 2.00 equiv.) was added dropwise. The solution was stirred for 10 min at –105 °C, then it was slowly warmed to room temperature. Water (5 mL) was added and the layers were separated. The organic layer was washed with Brine (2 x 5 mL) and the combined aqueous layers were extracted with Et_2O (3 x 5 mL). The organic layers were combined, dried over $MgSO_4$ and concentrated under vacuum. The crude product was purified by column chromatography, eluting with petrol:Et_2O 95:5, to give **4** as a colourless oil (75 mg, 69%, 94:6 e.r.). **$[\alpha]_D$** (20 °C, $CHCl_3$, *c* 1.0) = –3. **FT-IR** ν_{max} (film)/cm^{-1}: 2977, 1725, 1367, 1140, 846, 755. **^{1}H NMR** (400 MHz, $CDCl_3$): δ = 2.54 (1H, ap. sext, *J* 7.0 Hz, H^A), 1.42 (9H, s, H^B), 1.22 (6H, s, H^C), 1.21 (6H, s, H^C), 1.14 (3H, d, *J* 7.0 Hz, H^D), 1.07 (1H, dd, *J* 15.8, 7.0 Hz, H^E), 0.85 (1H, dd, *J* 15.8, 7.0 Hz, H^E). **^{13}C NMR** (100 MHz, $CDCl_3$): δ = 176.8 (C=O), 83.1 (2 x C), 79.6 (C), 36.4 (CH), 28.1 (3 x CH_3), 24.9 (4 x CH_3), 19.6 (CH_3). **^{11}B NMR** (96 MHz, $CDCl_3$): δ = 33. **HRMS** (ESI) Found: $[M+Na]^+$, 293.1904. $C_{14}H_{27}BNaO_4$ requires $[M+Na]^+$, 293.1897.

e.r. Determination: A solution of **4** (50 mg, 0.19 mmol, 1.0 equiv.) in THF (1 mL) was cooled to 0 °C, then aqueous NaOH 2M:H_2O_2 30% (2:1, 1 mL) was added dropwise. The biphasic solution was stirred vigorously for 1 h. Water (5 mL) and Et_2O (5 mL) were added and the layers were separated. The organic layer was washed with Brine (2 x 3 mL) and the combined aqueous layers were extracted with Et_2O (2 x 5 mL). The organic layers were combined, dried over $MgSO_4$ and concentrated under vacuum. The crude product was purified by flash column chromatography on silica gel, eluting with petrol:EtOAc 3:1, to give (*R*)-**23** as a colourless oil (70%). (*R*)-**23** was then converted into benzoate (*R*)-**23*a*** following the procedure described above (98%).

Chiral HPLC analysis (IA column, hexane:IPA 99:1 (10% of 10% IPA in hexane), 1 mL/min, 273 nm, t_R 8.020 min [(*S*), minor], 9.058 [(R), major]:

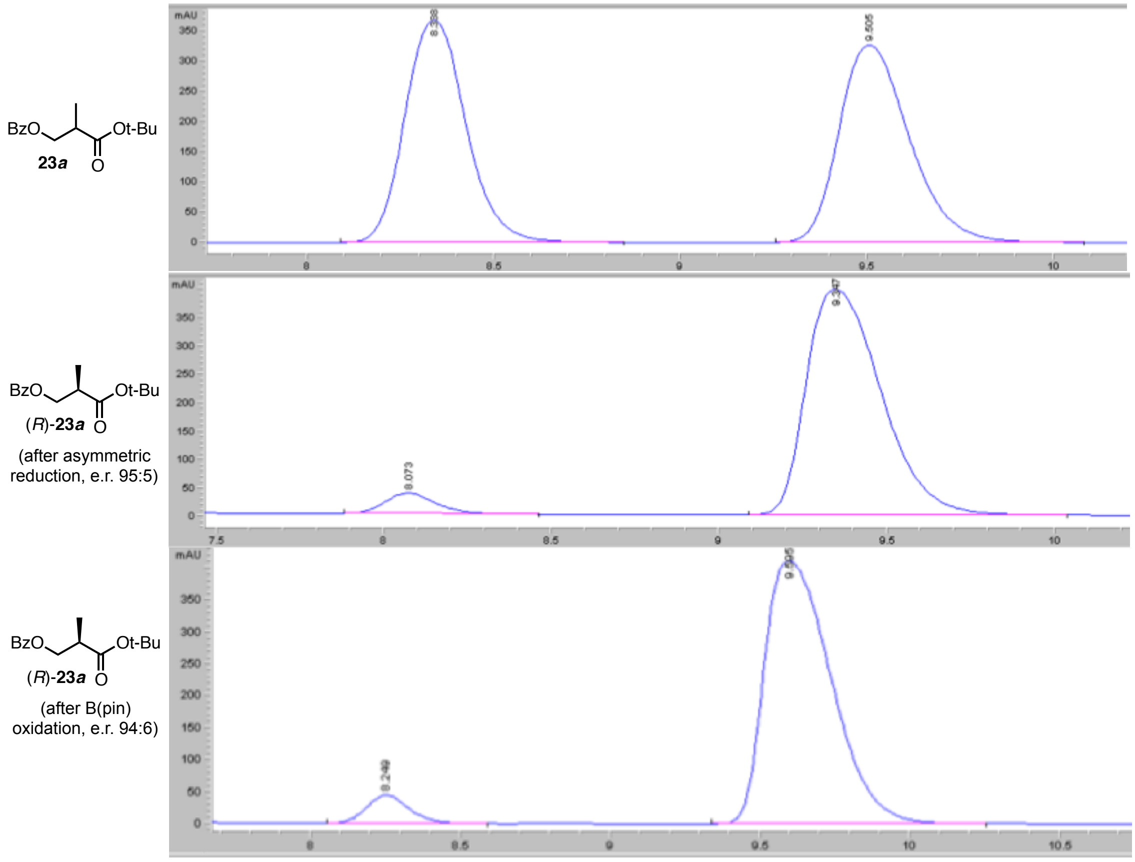

8.1. Model Lithiation-Borylation with Boronic Ester 4

(2*R*,4*S*)-*tert*-Butyl 6-(4-methoxyphenyl)-2-methyl-4-(4,4,5,5-tetramethyl-1,3,2-dioxaborolan-2-yl)hexanoate (34)

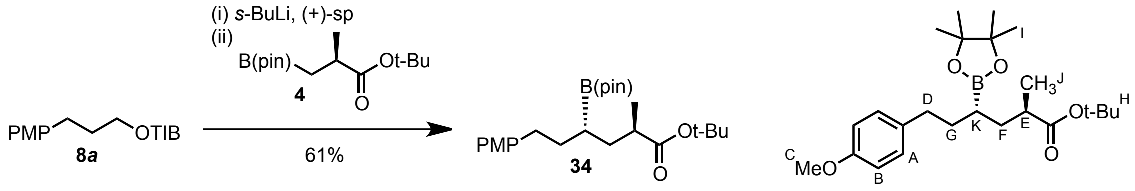

TIB ester **8*a***[4] (see TIB ester 8, section 2 for synthesis) (50 mg, 0.13 mmol, 1.30 equiv.) and (+)-sparteine (28 mg, 0.03 mL, 0.12 mmol, 1.20 equiv.) were dissolved in CPME (1.3 mL). The solution was cooled to –78 °C and *s*-BuLi (1.3 M in hexanes, 0.09 mL, 0.12 mmol, 1.20 equiv.) was added dropwise. The solution was stirred at –78 °C for 3 h, then a solution of **4** (26 mg, 0.1 mmol, 1.00 equiv.) in CPME (0.3 mL) was added dropwise. The solution was stirred for 45 min at –78 °C, after which ^{11}B NMR analysis of the crude mixture showed complete boronate complex formation [^{11}B NMR (92.6 MHz, no solvent) δ = 7]. The solution was warmed to 105 °C and stirred for 3 h. ^{11}B NMR analysis of the crude mixture showed full product formation [^{11}B NMR (92.6 MHz, no solvent) δ = 33]. Water (5 mL) and Et_2O (5 mL) were added and the layers were separated. The organic layer was washed with Brine (2 x 5 mL) and the combined aqueous layers were extracted with Et_2O (3 x 5 mL). The organic layers were combined, dried over $MgSO_4$ and concentrated under vacuum. The crude product was purified by column chromatography on silica gel, eluting with petrol:EtOAc 97:3 to 95:5, to give **34** as an oil (25 mg, 61%). **[α]D** (20 °C, $CHCl_3$, *c* 1.0) = +2. **FT-IR** ν_{max} (film)/cm^{-1}: 2926, 1725, 1512, 1244, 1142, 1037, 753. **^{1}H NMR** (400 MHz, $CDCl_3$): δ = 7.10 (2H, d, *J* 8.2 Hz, H^A), 6.82 (2H, d, *J* 8.2 Hz, H^B), 3.79 (3H, s, H^C), 2.54 (2H, m, H^D), 2.39 (1H, sext, *J* 7.0 Hz, H^E), 1.83 (1H, m, H^F), 1.69 (2H, m, H^G), 1.49-1.37 (10H, m, H^F & H^H), 1.27 (12H, s, H^I), 1.08 (3H, d, *J* 7.0 Hz, H^J), 0.87 (1H, m, H^K). **^{13}C NMR** (100 MHz, $CDCl_3$): δ = 176.3 (C=O), 157.6 (C), 135.0 (C), 129.2 (2 x CH), 113.7 (2 x CH), 83.0 (2 x C), 79.6 (C), 55.2 (CH_3), 39.3 (CH), 34.9 (CH_2), 34.6 (CH_2), 33.4 (CH_2), 28.1 (3 x CH_3), 24.9 (2 x CH_3), 24.8 (2 x CH_3), 16.9 (CH_3). **^{11}B NMR** (96 MHz, $CDCl_3$): δ = 33. **HRMS** (ESI) Found: $[M+Na]^+$, 441.2779. $C_{24}H_{39}BNaO_5$ requires $[M+Na]^+$, 441.2787.

9. Synthesis of Building Block 26

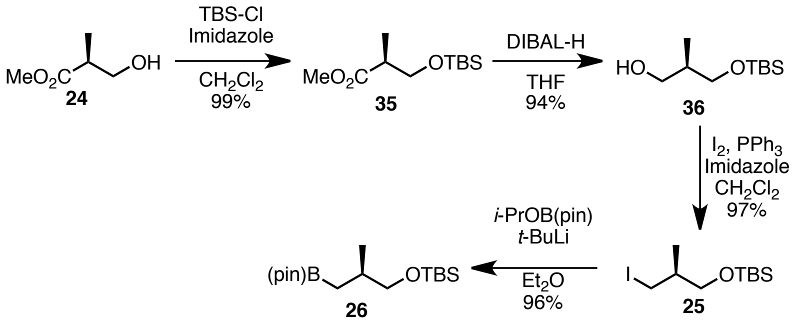

Methyl (*S*)-3-((*tert*-butyldimethylsilyl)oxy)-2-methylpropanoate (35)

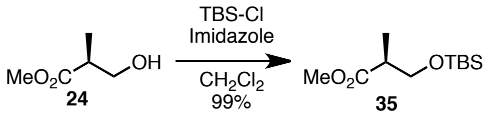

Following a literature procedure,[15] to a solution of methyl (*S*)-3-hydroxy-2-methylpropanoate (**24**) (1.00 g, 0.934 mL, 8.47 mmol, 1.00 equiv.) in dichloromethane (40 mL) was added imidazole (1.15 g, 16.94 mmol, 2.00 equiv.) followed by *tert*-butyldimethylsilyl chloride (1.53 g, 10.16 mmol, 1.20 equiv.). The solution was stirred at room temperature for 16 h, then aqueous NH_4Cl (30 mL) was added. The layers were separated, the organic layer was washed with Brine (2 x 10 mL) and the combined aqueous layers were extracted with dichloromethane (3 x 10 mL). The organic layers were combined, dried over $MgSO_4$ and concentrated under vacuum. The crude product was purified by column chromatography on silica gel, eluting with petrol:EtOAc 97:3, to give **35** as a colourless oil (1.97 g, 99%). **^{1}H NMR** (400 MHz, $CDCl_3$): δ = 3.77 (1H, dd, *J* = 9.7, 6.8 Hz), 3.67 (3H, s), 3.64 (1H, dd, *J* = 9.7, 6.3 Hz), 2.65 (1H, sextet, *J* = 6.9 Hz), 1.13 (3H, d, *J* = 6.9 Hz), 0.87 (9H, s), 0.03 (6H, s). **^{13}C NMR** (100 MHz, $CDCl_3$): δ = 175.5 (C=O), 65.2 (CH_2), 51.5 (CH_3), 42.5 (CH), 25.8 (3 x CH_3), 18.2 (CH_3), 13.5 (C), –5.5 (2 x CH_3). Data in accordance with the literature. [15]

(*R*)-3-((*tert*-Butyldimethylsilyl)oxy)-2-methylpropan-1-ol (36)

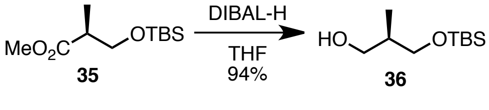

Following a literature procedure,[15] a solution of **35** (2.0 g, 8.60 mmol, 1.00 equiv.) in dichloromethane (40 mL) was cooled down to –78 °C. DIBAL-H (1.0 M in CH_2Cl_2, 25.8 mL, 25.8 mmol, 3.00 equiv.) was added dropwise to the solution, which was then stirred for 1 h at –78 °C. The solution was warmed to room temperature. Et_2O (51.6

mL) was added, followed by water (2.3 mL), dropwise, while stirring vigorously. The solution was stirred for 30 min, then 1.3 M NaOH (7.0 mL) was added. The aluminium salts were filtered, washing with EtOAc. The solvent was removed under vacuum. The crude product was purified by column chromatography on silica gel, eluting with petrol:EtOAc 90:10 to 85:15, to give **36** as a colourless oil (1.65 g, 94%). **^{1}H NMR** (400 MHz, $CDCl_3$): δ = 3.73 (1H, dd, *J* = 9.7, 4.5 Hz), 3.67-3.59 (2H, m), 3.54 (1H, dd, *J* = 9.7, 8.0 Hz), 1.94 (1H, m), 0.90 (9H, s), 0.83 (3H, d, *J* = 7.4 Hz), 0.07 (6H, s). **^{13}C NMR** (100 MHz, $CDCl_3$): δ = 68.8 (CH_2),68.3 (CH_2), 37.0 (CH), 25.9 (3 x CH_3), 18.2 (CH_3), 13.1 (C), −5.5 (2 x CH_3). Data in accordance with the literature. [15]

(*S*)-*tert*-Butyl(3-iodo-2-methylpropoxy)dimethylsilane (25)

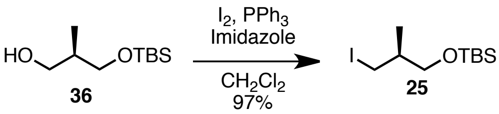

To a solution of **36** (200 mg, 0.98 mmol, 1.00 equiv.) in CH_2Cl_2 (10 mL), covered in Al foil, were added imidazole (133 mg, 1.96 mmol, 2.00 equiv.), triphenylphosphine (385 mg, 1.47 mmol, 1.50 equiv.) and iodine (373 mg, 1.47 mmol, 1.50 equiv.). The solution was stirred at room temperature for 4 h, then aqueous $Na_2S_2O_3$ (10 mL) was added. The layers were separated, the organic layer was washed with Brine (2 x 5 mL) and the combined aqueous layers were extracted with CH_2Cl_2 (2 x 10 mL). The organic layers were combined, dried over $MgSO_4$ and concentrated under vacuum. The crude product was purified by column chromatography on silica gel, eluting with neat pentane to pentane:Et_2O 97:3, to give **26** as a colourless oil (280 mg, 91%). **^{1}H NMR** (400 MHz, $CDCl_3$): δ = 3.53 (1H, dd, *J* = 10.1, 5.1 Hz), 3.40 (1H, dd, *J* = 10.1, 6.9 Hz), 3.31 (1H, dd, *J* = 9.5, 5.1 Hz), 3.25 (1H, dd, *J* = 9.3, 5.6 Hz), 1.65 (1H, m), 0.95 (3H, d, *J* = 6.8 Hz), 0.90 (9H, s), 0.07 (6H, s). **^{13}C NMR** (100 MHz, $CDCl_3$): δ = 66.7 (CH_2), 37.4 (CH), 25.9 (3 x CH_3), 18.2 (CH_3), 17.2 (C), 13.7 (CH_2), -5.4 (2 x CH_3). Data in accordance with the literature. [15]

(*R*)-*tert*-Butyldimethyl(2-methyl-3-(4,4,5,5-tetramethyl-1,3,2-dioxaborolan-2-yl)propoxy)silane (26)

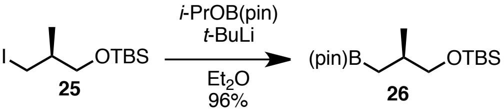

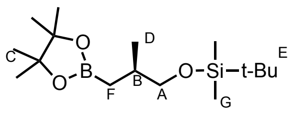

A solution of **25** (530 mg, 1.69 mmol, 1.00 equiv.) and *i*-PrOB(pin) (377 mg, 0.42 mL,

2.03 mmol, 1.20 equiv.) in Et_2O (10 mL) was cooled down to –105 °C, then *t*-BuLi (1.9 M in pentane, 1.78 mL, 3.37 mmol, 2.00 equiv.) was added. The solution was stirred at –105 °C for 10 min, then slowly let warm to room temperature. Aqueous NH_4Cl (10 mL) was added and the layers were separated. The organic layer was washed with Brine (2 x 5 mL) and the combined aqueous layers were extracted with Et_2O (3 x 5 mL). The organic layers were combined, dried over $MgSO_4$ and concentrated under vacuum. The crude product was purified by column chromatography on silica gel, eluting with petrol:EtOAc 99.5:0.5, to give **26** as a colourless oil (435 mg, 82%). **$[\alpha]_D$** (20 °C, $CHCl_3$, *c* 1.0) = +4. **FT-IR** ν_{max} (film)/cm^{-1}: 2929, 1370, 1145, 1082, 834, 773. **^{1}H NMR** (400 MHz, $CDCl_3$): δ = 3.43 (1H, dd, J = 9.6, 5.8 Hz, H^A), 3.31 (1H, dd, J = 9.6, 7.0 Hz, H^A), 1.84 (1H, m, H^B), 1.24 (12H, s, H^C), 0.92 (3H, d, J = 6.5 Hz, H^D), 0.89 (9H, s, H^E), 0.86 (1H, dd, J = 15.4, 5.8 Hz, H^F), 0.58 (1H, dd, J = 15.4, 8.8 Hz, H^F), 0.03 (6H, s, H^G). **^{13}C NMR** (100 MHz, $CDCl_3$): δ = 100.0 (C), 82.8 (2 x C), 70.0 (CH_2), 32.2 (CH), 26.0 (3 x CH_3), 24.9 (2 x CH_3), 24.8 (2 x CH_3), 19.0 (CH_3), 18.0 (C) –5.33 (2 x CH_3). **^{11}B NMR** (96.2 MHz, $CDCl_3$): δ = 33.4. **HRMS** (ESI) Found: $[M+H]^+$, 315.2515. $C_{16}H_{35}BO_3Si$ requires $[M+H]^+$, 315.2525.

10. Endgame

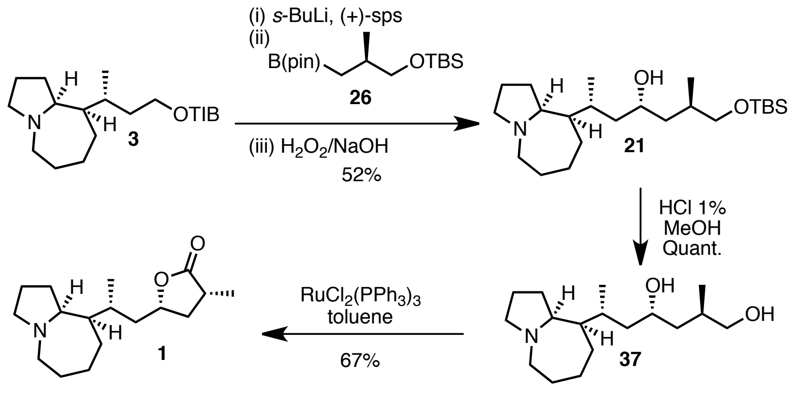

(2*R*,4*S*,6*R*)-1-((*tert*-Butyldimethylsilyl)oxy)-2-methyl-6-((9*R*,9a*S*)-octahydro-1*H*-pyrrolo[1,2-*a*]azepin-9-yl)heptan-4-ol (21)

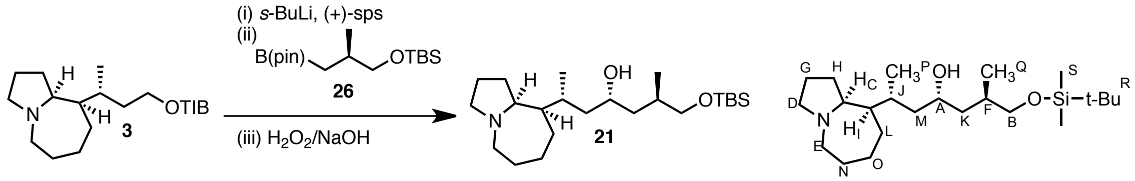

3 (250 mg, 0.57 mmol, 1.00 equiv.) and (+)-sparteine surrogate (330 mg, 0.33 mL, 1.70 mmol, 3.00 equiv.) were dissolved in CPME (6 mL). The solution was cooled to −78 °C and *s*-BuLi (1.3 M in hexanes, 1.30 mL, 3.00 equiv.) was added dropwise. The solution was stirred at –78 °C for 1 h, then a solution of **26** (534 mg, 1.70 mmol, 3.00 equiv.) in CPME (1 mL) was added dropwise. The solution was stirred for 45 min at –78 °C, after which ^{11}B NMR analysis of the crude mixture showed complete boronate complex formation [^{11}B NMR (92.6 MHz, no solvent) δ = 7]. The solution was warmed to 60 °C and stirred for 16 h. ^{11}B NMR analysis of the crude mixture showed full product formation [^{11}B NMR (92.6 MHz, no solvent) δ = 33]. CPME was removed under vacuum and THF (6 mL) was added. The solution was cooled to 0 °C and an aqueous solution of 2 M NaOH and 30% H_2O_2 (2:1 v/v, 6 mL) was added dropwise. The solution was stirred vigorously for 2 h at 0 °C, then it was warmed to room temperature and diluted with water (10 mL) and EtOAc (10 mL). The layers were separated, the organic layer was washed with Brine (2 x 10 mL) and the combined aqueous layers were extracted with EtOAc (3 x 10 mL). The organic layers were combined, dried over $MgSO_4$ and concentrated under vacuum. The crude product was purified by column chromatography on silica gel, eluting with $CHCl_3$:MeOH:NH_4OH 99.9:0:0.1 to 94.9:5:0.1, to give **21** as an oil (117 mg, 52%). **[α]D** (20 °C, $CHCl_3$,

c 1.0) = –2. **FT-IR** ν_{max} (film)/cm^{-1}: 3367, 2926, 1461, 1250, 1087, 834. **^{1}H NMR** (500 MHz, $CDCl_3$): δ = 3.77 (1H, m, H^A), 3.54 (1H, dd, J 10.1, 5.3 Hz, H^B), 3.44 (1H, dd, J 10.1, 7.4 Hz, H^B), 3.14-2.95 (3H, m, H^C & H^D & H^E), 2.69-2.51 (2H, m, H^D & H^E), 1.89-1.78 (3H, m, H^F & H^G & H^H), 1.76-1.62 (6H, m, H^G & H^H & H^I & H^J & H^K), 1.52 (2H, m, H^L), 1.44-1.28 (6H, m, H^M & H^N & H^O), 0.97 (3H, d, J 6.1 Hz, H^P), 0.94-0.90 (12H, m, H^Q & H^R), 0.09 (6H, s, H^S). **^{13}C NMR** (125 MHz, $CDCl_3$): δ = 69.3 (CH_2), 68.6 (CH), 65.4 (CH), 54.3 (CH_2), 52.4 (CH_2), 46.0 (CH), 43.1 (CH_2), 42.2 (CH_2), 34.1 (CH), 29.7 (CH_2), 28.3 (CH_2), 28.0 (CH_2), 26.0 (CH_2), 25.9 (3 x CH_3), 23.8 (CH_2), 20.0 (CH_3), 18.3 (C), 17.8 (CH_3), –5.4 (2 x CH_3). **HRMS** (ESI) Found: $[M+H]^+$, 398.3450. $C_{23}H_{48}NO_2Si$ requires $[M+H]^+$, 398.3449.

(2*R*,4*S*,6*R*)-2-Methyl-6-((9*R*,9a*S*)-octahydro-1*H*-pyrrolo[1,2-*a*]azepin-9-yl)heptane-1,4-diol (37)

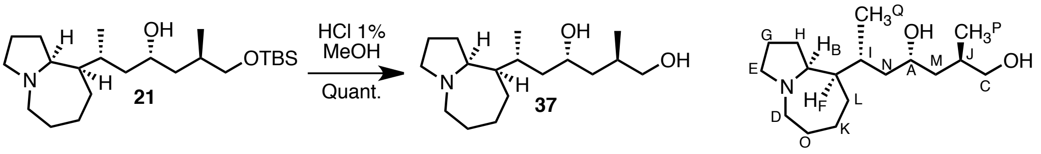

21 (150 mg, 0.38 mmol, 1.0 equiv.) was dissolved in MeOH (4 mL). 1% aqueous HCl (0.5 mL) was added and the solution was stirred for 2 h at room temperature. Solid $NaHCO_3$ was added, the solids were filtered washing with CH_2Cl_2 and the solvent was evaporated under vacuum, to give **37** as an oil (105 mg, quantitative crude yield), which was used in the next step without further purification.

The crude product could also be purified by reverse phase flash chromatography (t_R 7 min) to give **37** as the formate salt, as an oil (79 mg, 73%). **[α]$_D$** (20 °C, $CHCl_3$, c 1.0) = –21. **FT-IR** ν_{max} (film)/cm^{-1}: 3319, 2926, 1594, 1456, 1337, 1036, 761. **^{1}H NMR** (500 MHz, $CDCl_3$): δ = 8.63 (1H, s, $\underline{H}CO_2^-$) 4.00 (1H, m, H^A), 3.90 (1H, m, H^B), 3.66 (1H, dd, J 10.9, 3.8 Hz, H^C), 3.56 (1H, m, H^D), 3.40-3.30 (2H, m, H^C & H^D), 3.14 (1H, ap. dd, J 14.5, 6.6 Hz, H^E), 2.94 (1H, m, H^E), 2.41 (1H, m, H^F), 2.08 (1H, m, H^G), 2.03-1.83 (7H, m, H^G & H^H & H^I & H^J & H^K), 1.76-1.57 (4H, m, H^L & H^M), 1.53-1.37 (4H, m, H^N & H^O), 0.90 (3H, d, J 6.8 Hz, H^P), 0.89 (3H, d, J 7.3 Hz, H^Q). **^{13}C NMR** (125 MHz, $CDCl_3$): δ = 169.3 (formate $H\underline{C}O_2^-$) 68.9 (CH_2), 67.9 (CH), 64.4 (CH), 52.2 (CH_2), 51.5 (CH_2), 44.6 (CH_2), 42.0 (CH_2), 39.1 (CH), 34.7 (2 x CH), 28.4 (CH_2), 28.1 (CH_2), 27.8 (CH_2), 23.7 (CH_2), 22.9 (CH_2), 18.6 (CH_3), 17.7 (CH_3). **HRMS** (ESI) Found: $[M+H]^+$, 284.2594. $C_{17}H_{34}NO_2$ requires $[M+H]^+$, 284.2584.

(3*R*,5*S*)-3-Methyl-5-((*R*)-2-((9*R*,9a*S*)-octahydro-1*H*-pyrrolo[1,2-*a*]azepin-9-yl)propyl)dihydrofuran-2(3*H*)-one [(–)-stemaphylline, 1]

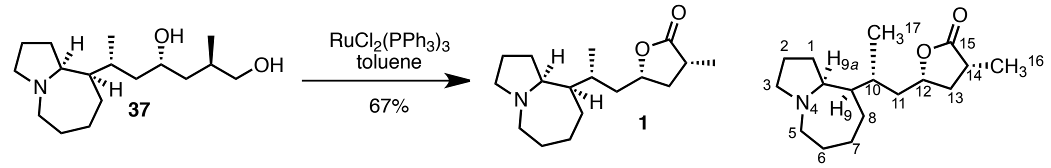

To a solution of **37** (6 mg, 0.021 mmol, 1.0 equiv.) in toluene (2 mL) was added $RuCl_2(PPh_3)_3$[16] (30 mg, 0.032 mmol, 1.5 equiv.). The mixture was stirred at room temperature for 16 h, then it was filtered through silica washing with CH_2Cl_2:MeOH:Et_2NH 98.8:1.0:0.2. The crude product was purified by preparative TLC in an aluminium-backed plate pre-coated (0.25 mm) with Merck Silica Gel 60 F254. To visualise the product on the preparative TLC plate, a reference sample was run on the same plate, then cut and stained with $KMnO_4$ (see picture below). **1** was obtained as an oil (4 mg, 67%). **[α]$_D$** (20 °C, $CHCl_3$, *c* 0.2) = –31 [lit.[17] for (–)-**1**, **[α]$_D$** (20 °C, $CHCl_3$, *c* 0.54) = –36.7]. **FT-IR** ν_{max} (film)/cm^{-1}: 2928, 1767, 1667, 1454, 1376, 1173, 1001, 927. **^{1}H NMR** (600 MHz, $CDCl_3$): δ = 4.40 (1H, m, H^{12}), 3.01 (1H, m, H^3), 2.94 (1H, m, H^5), 2.89 (1H, m, H^{9a}), 2.64 (1H, m, H^{14}), 2.53-2.44 (3H, m, H^3 & H^5 & H^{13}), 2.01 (1H, m, H^{11}), 1.82-1.74 (3H, m, H^1 & H^7), 1.72-1.63 (4H, m, H^2 & H^{10} & H^{11}), 1.61-1.42 (6H, m, H^6 & H^8 & H^9 & H^{13}), 1.32 (1H, m, H^7), 1.26 (3H, d, *J* 7.2 Hz, H^{16}), 0.99 (3H, d, *J* 6.6 Hz, H^{17}). **^{13}C NMR** (150 MHz, $CDCl_3$): δ = 179.8 (C^{15}), 78.8 (CH^{12}), 65.1 (CH^{9a}), 54.8 (CH_2^3), 52.6 (CH_2^5), 46.8 (CH^9), 39.6 (CH_2^{11}), 38.0 (CH_2^{13}), 36.0 (CH^{14}), 32.5 (CH^{10}), 28.9 (CH_2^8), 28.5 (CH_2^1), 27.9 (CH_2^7), 26.0 (CH_2^6), 24.0 (CH_2^2), 19.6 (CH_3^{17}), 15.2 (CH_3^{16}). **HRMS** (ESI) Found: $[M+H]^+$, 280.2276. $C_{17}H_{34}NO_2$ requires $[M+H]^+$, 280.2271. Data in accordance with the literature[17] (See Table 3 for NMR data comparison between natural and synthetic (–)-**1**).

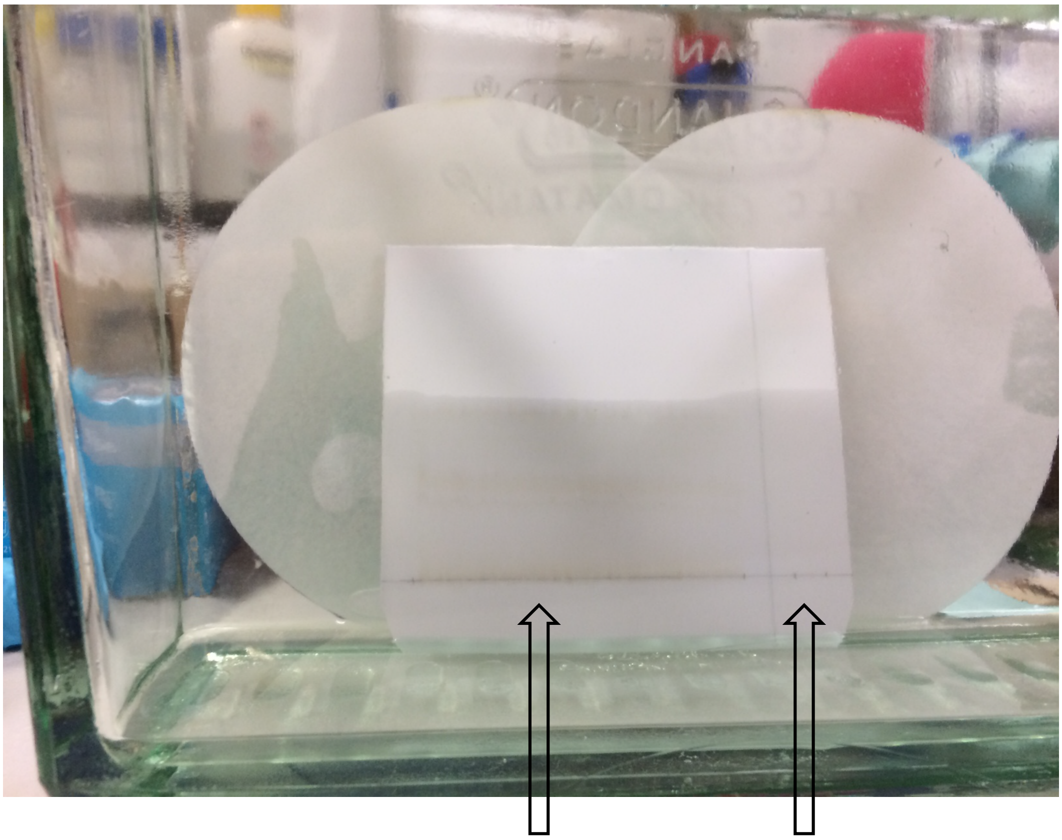

NOTE: Substantial degradation of the natural product was observed when it was kept as a solution in $CDCl_3$ for several days. The following ^{1}H NMR spectra correspond to the same sample of (–)-**1**; the bottom one was taken 72 h after the top one.

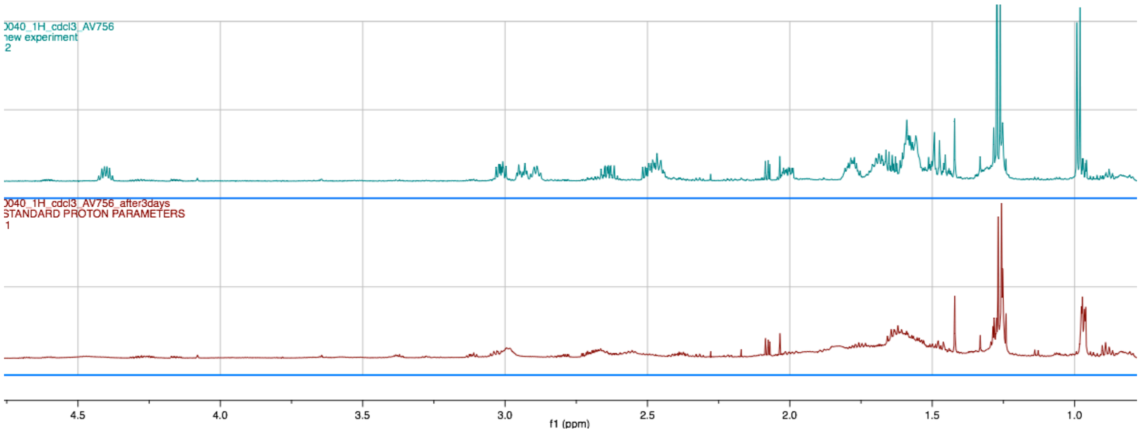

Table 3. Comparison of ^1H and ^{13}C NMR spectra between natural[17] and synthetic (–)-stemaphylline [(–)-**1**].

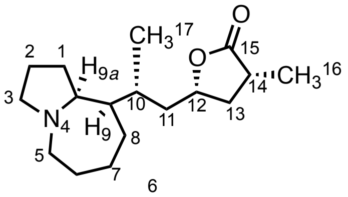

	δ_H		δ_C	
**Position**	**Natural**	**Synthetic**	**Natural**	**Synthetic**
**1**	1.79 (m)	1.82-1.74 (m)	28.1	28.5
**2**	1.72 (m)	1.72-1.63 (m)	23.8	24.0
**3**	3.01 (m)	3.01 (m)	54.3	54.8
	2.51 (m)	2.53-2.44 (m)		
**5**	2.94 (m)	2.94 (m)	52.3	52.6
	2.51 (m)	2.53-2.44 (m)		
**6**	1.50 (m)	1.61-1.42 (m)	25.9	26.0
**7**	1.80 (m)	1.82-1.74 (m)	27.7	27.9
	1.33 (m)	1.32 (m)		
**8**	1.58 (m)	1.61-1.42 (m)	28.2	28.9
**9**	1.62 (m)	1.61-1.42 (m)	45.9	46.7
**9*a***	2.93 (m)	2.94 (m)	64.8	65.1
**10**	1.70 (m)	1.72-1.63 (m)	32.5	32.5
**11**	2.00 (m)	2.01 (m)	39.5	39.6
	1.64 (m)	1.72-1.63 (m)		
**12**	4.39 (m)	4.40 (m)	78.4	78.8
**13**	2.51 (m)	2.53-2.44 (m)	37.8	38.0
	1.48 (m)	1.61-1.42 (m)		
**14**	2.61 (m)	2.64 (m)	35.8	36.0
**15**	-	-	179.6	179.8
**16**	1.26 (d, *J* 7.0)	1.26 (d, *J* 7.0)	15.0	15.2
**17**	0.98 (d, *J* 6.5)	0.99 (d, *J* 6.4)	19.2	19.6

11. ^{1}H-NMR and ^{13}C-NMR Spectra

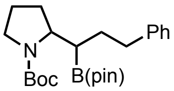

10

500 MHz, DMSO-d_6, 90 °C

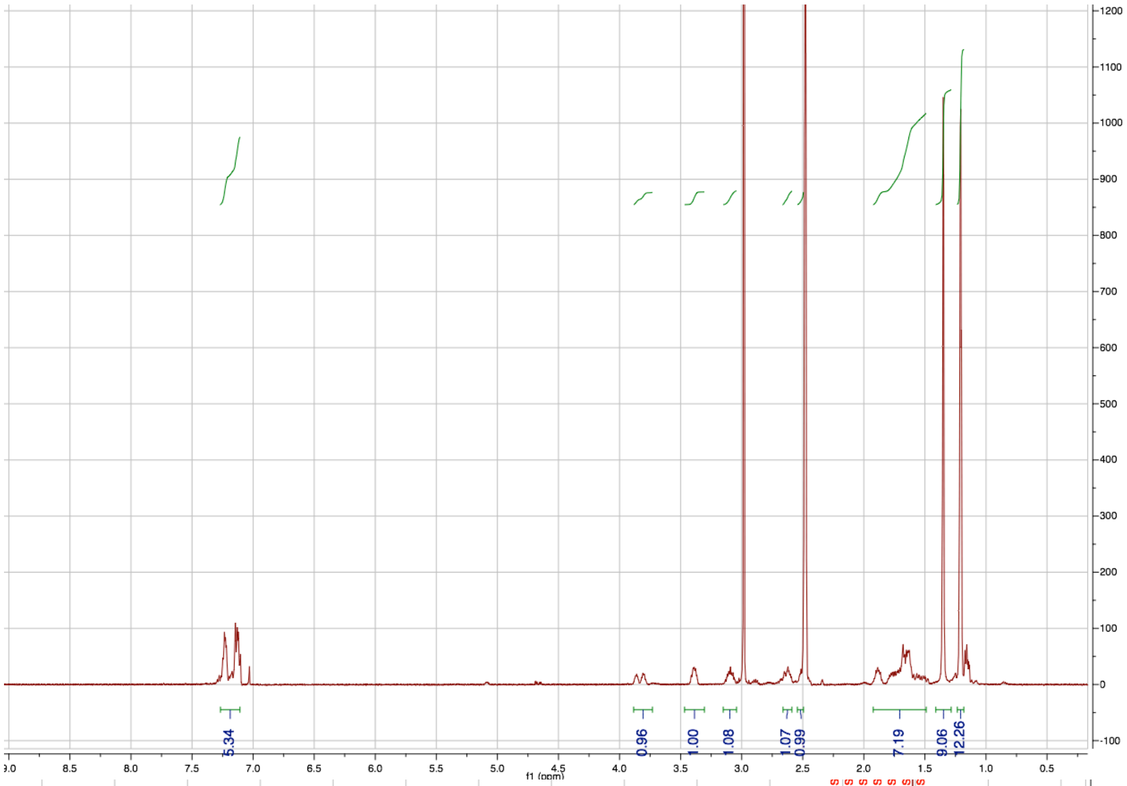

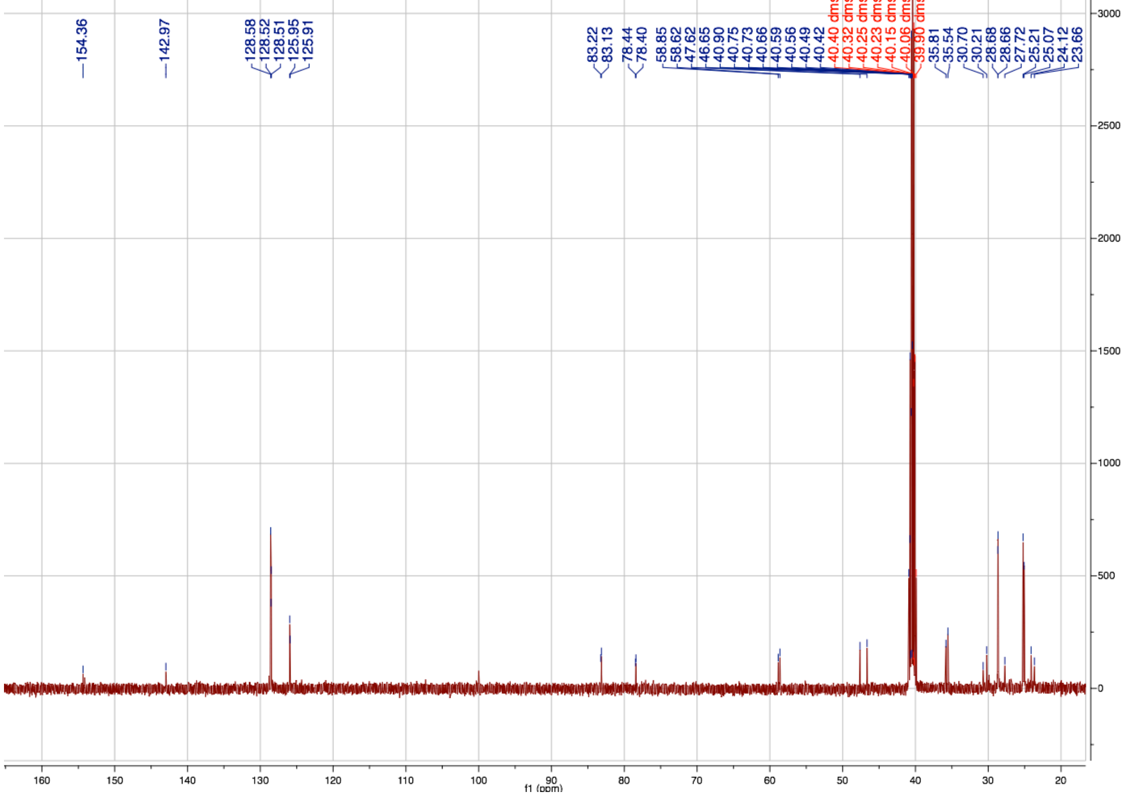

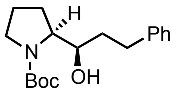

(*S*, *R*)-**11**

500 MHz, DMSO-d_6, 90 °C

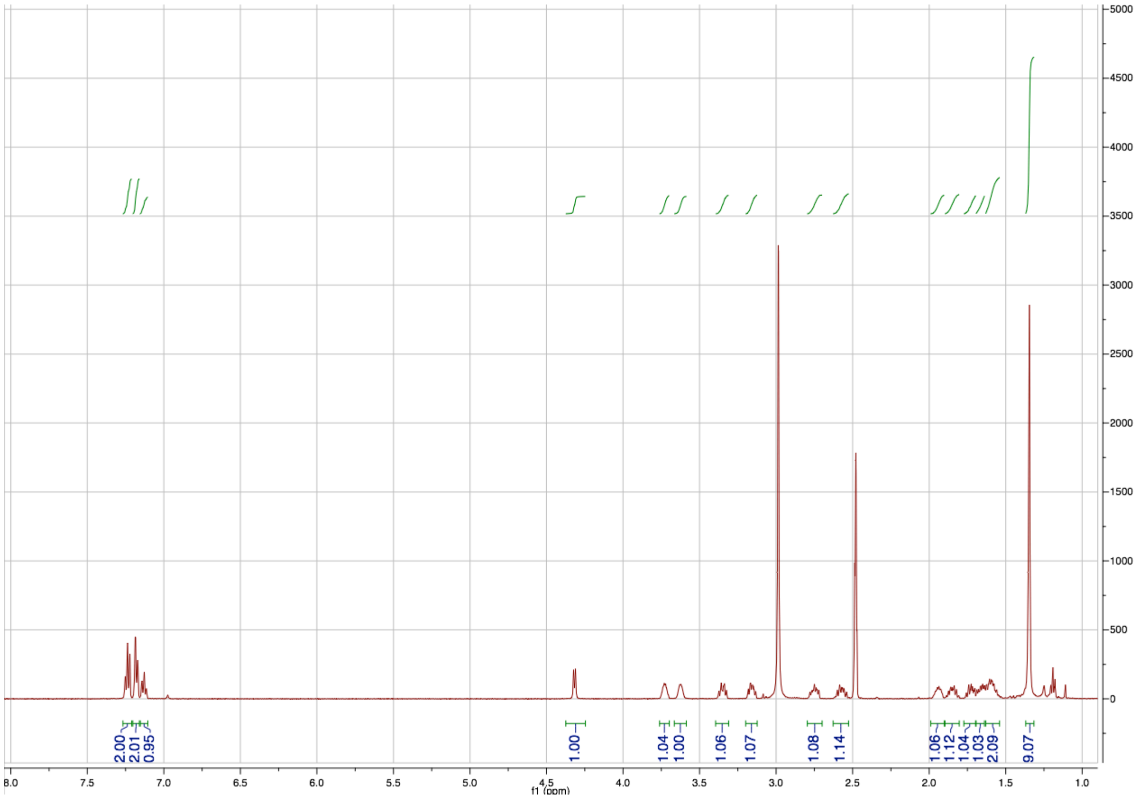

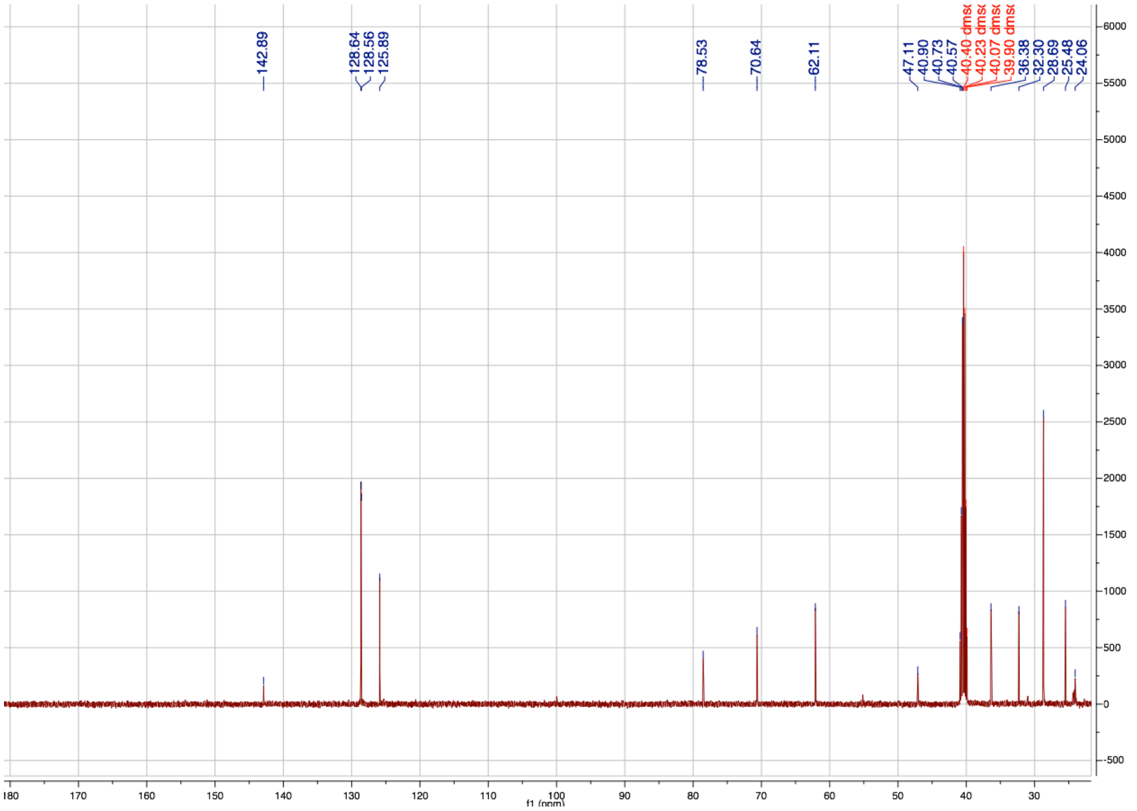

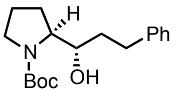

(*S*, *S*)-**11**

500 MHz, DMSO-d_6, 90 °C

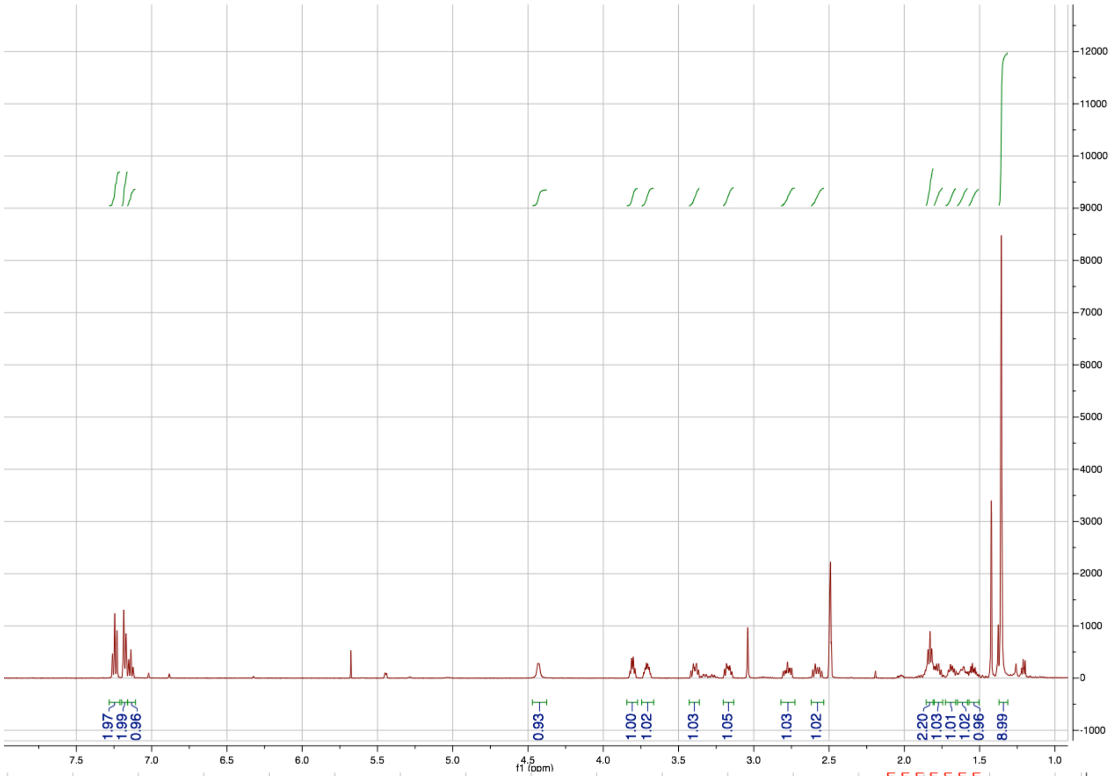

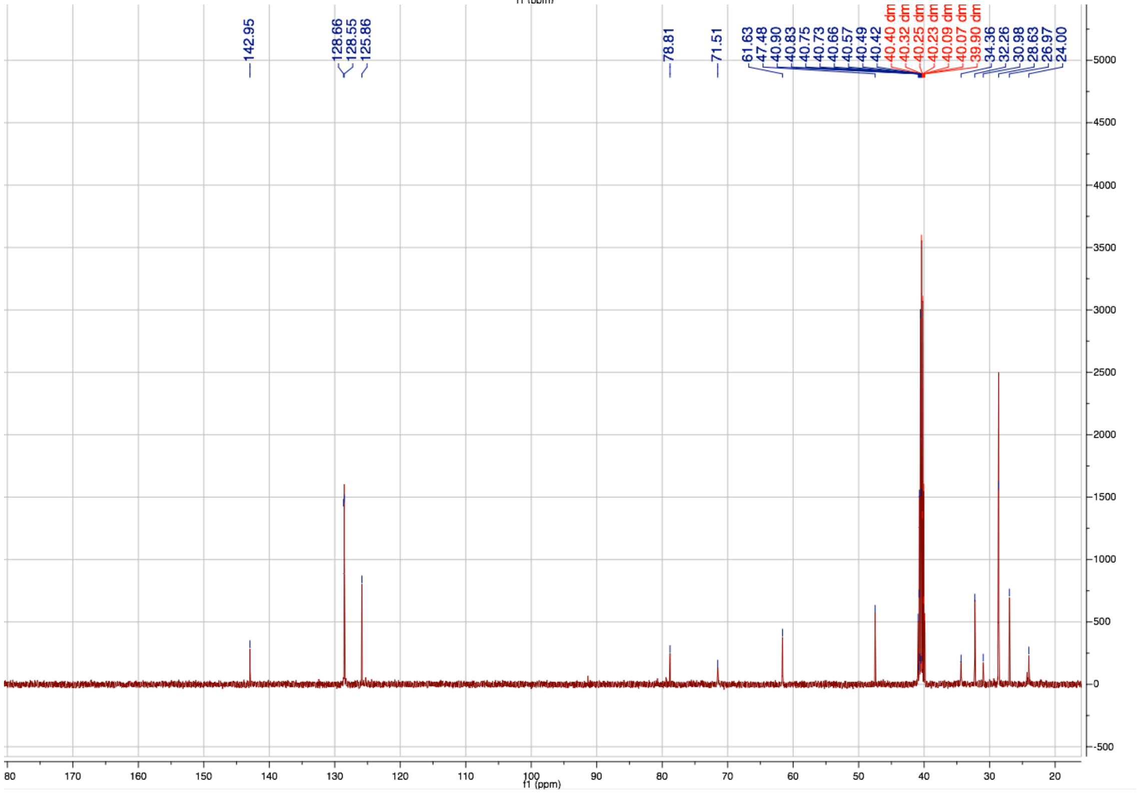

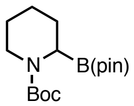

15

400 MHz, $CDCl_3$

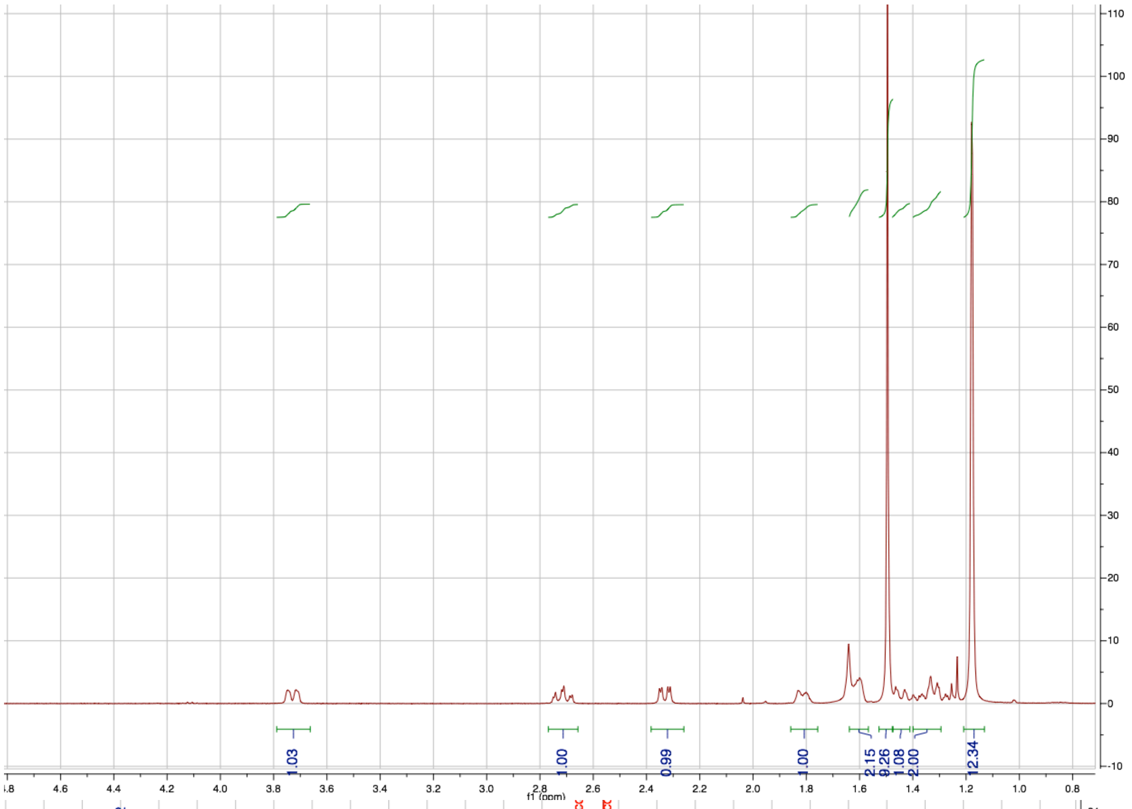

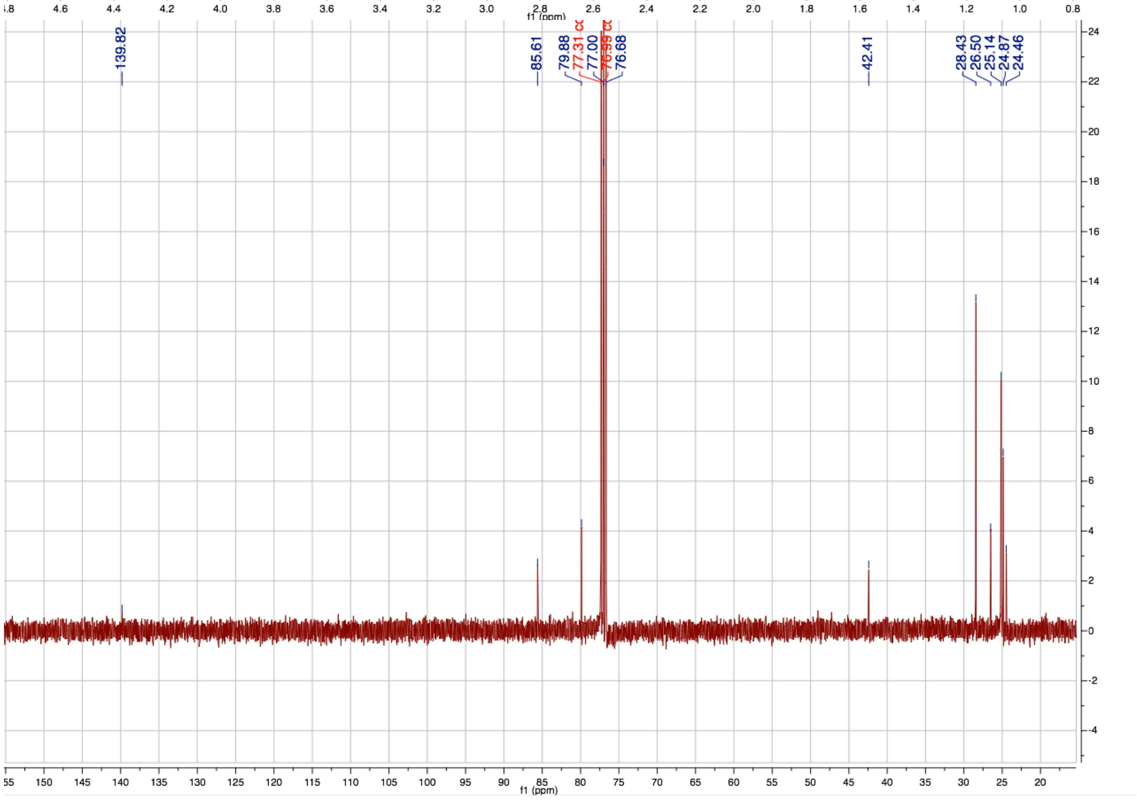

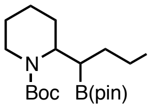

16

500 MHz, DMSO-d_6, 90 °C

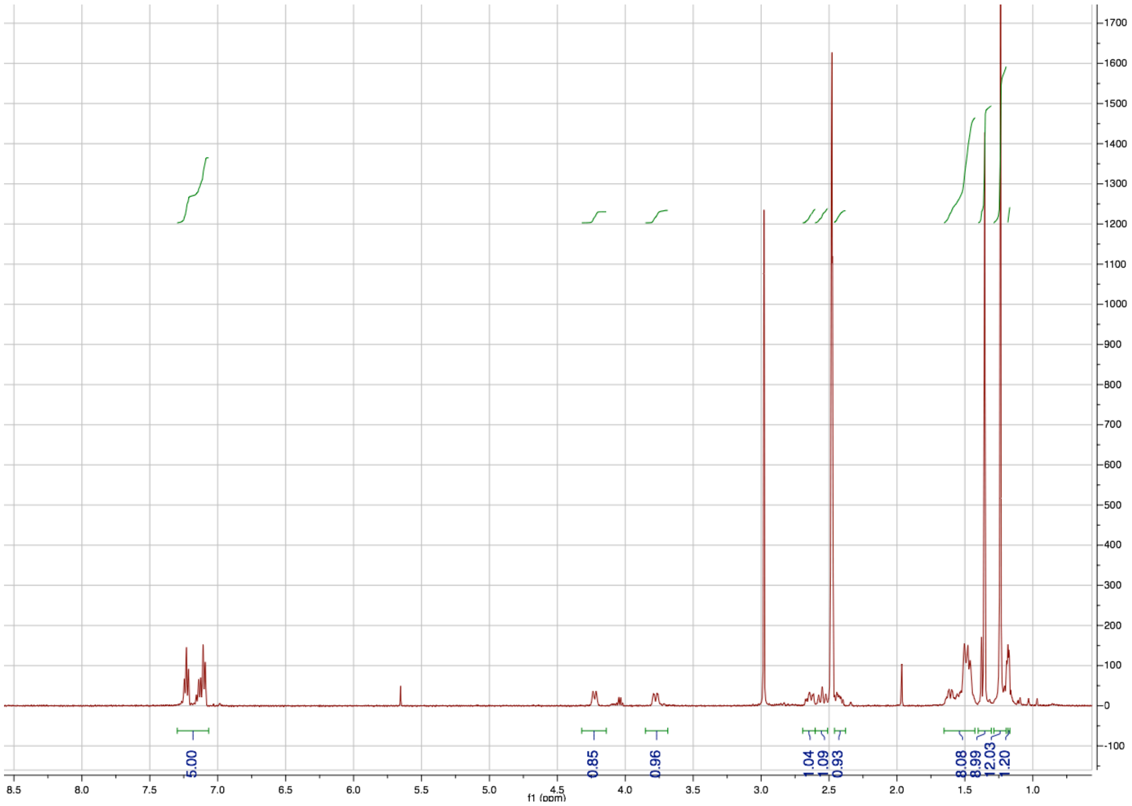

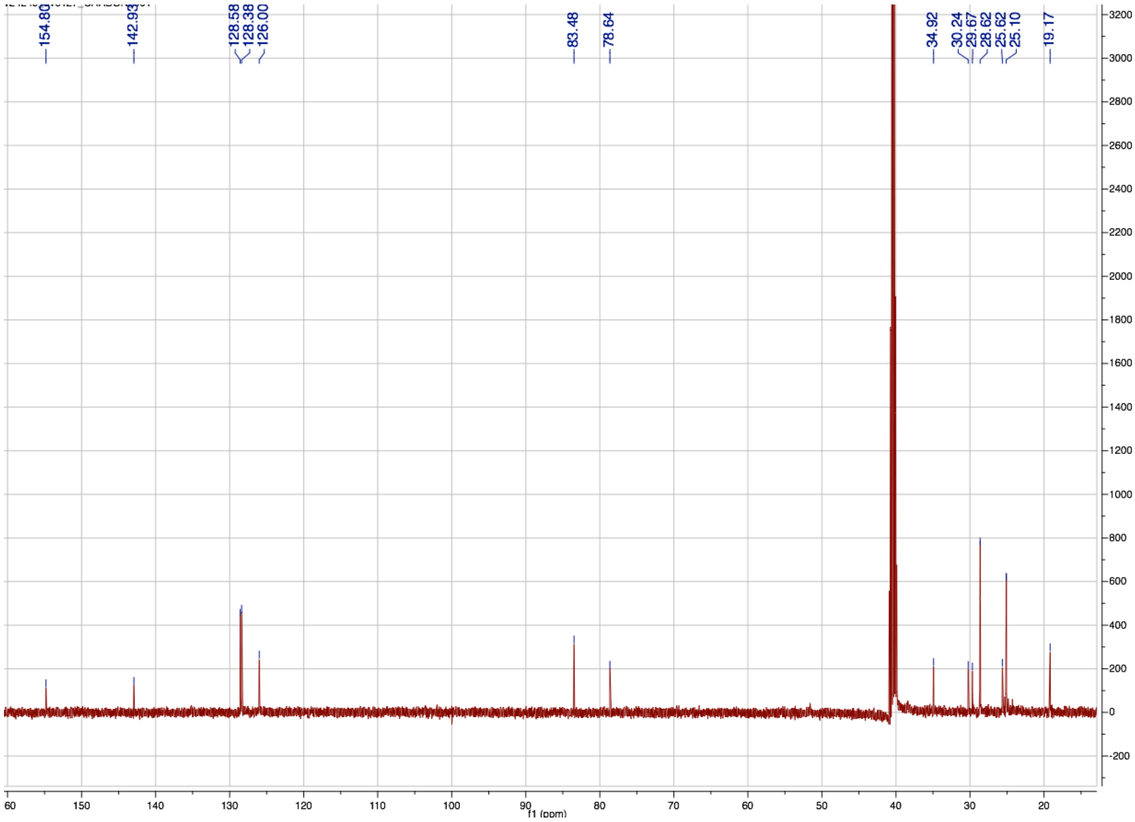

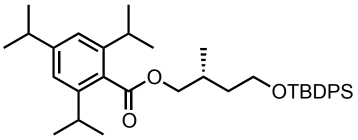

6

400 MHz, $CDCl_3$

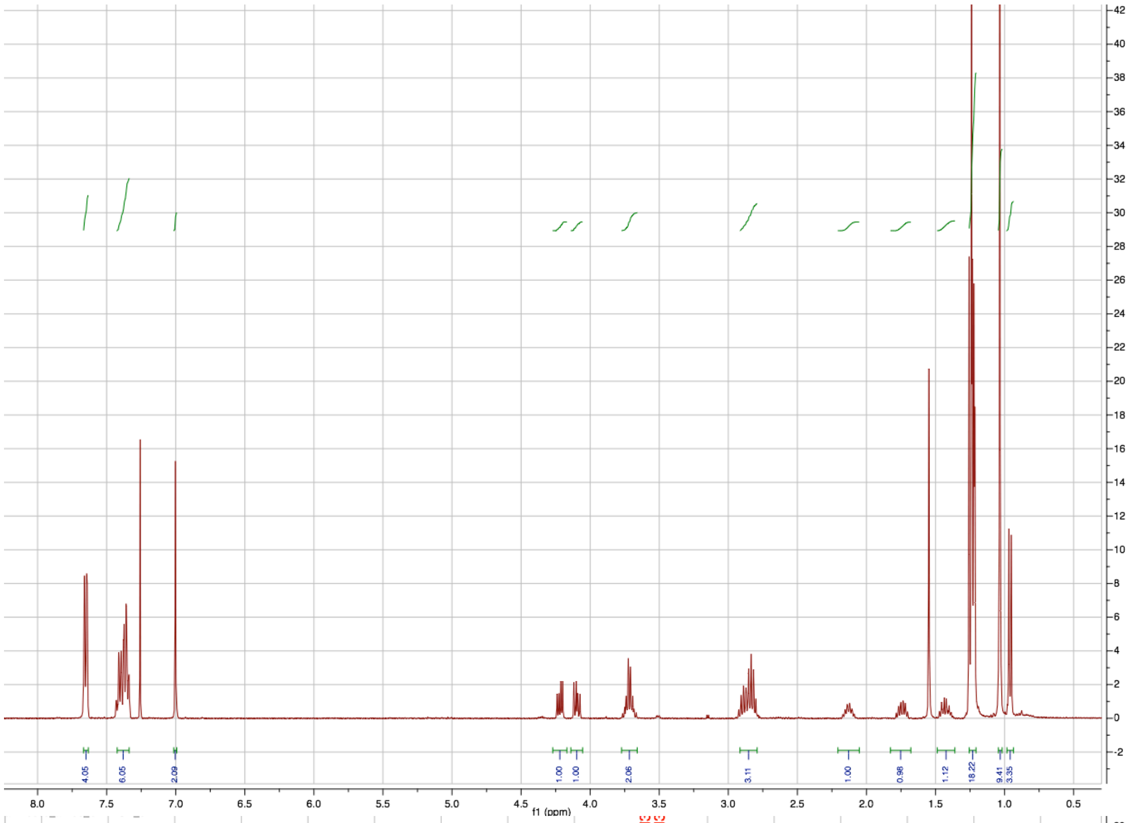

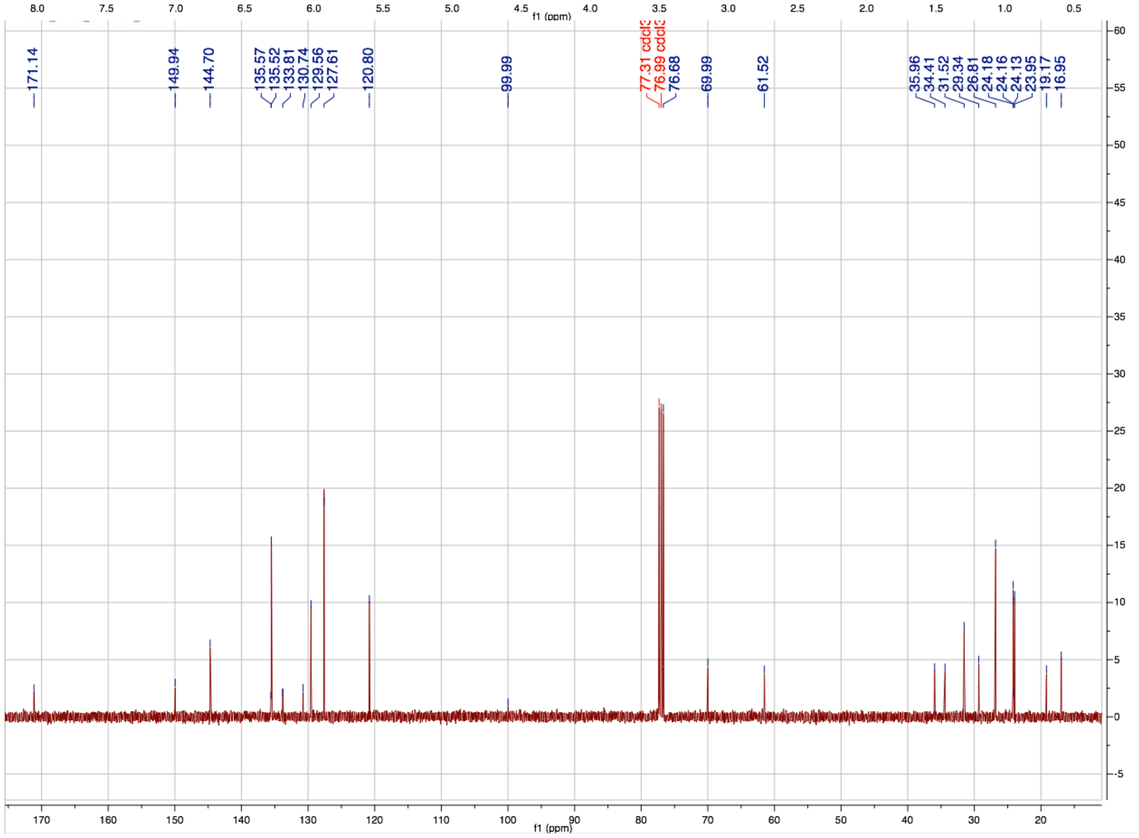

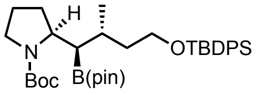

18

500 MHz, DMSO-d_6, 90 °C

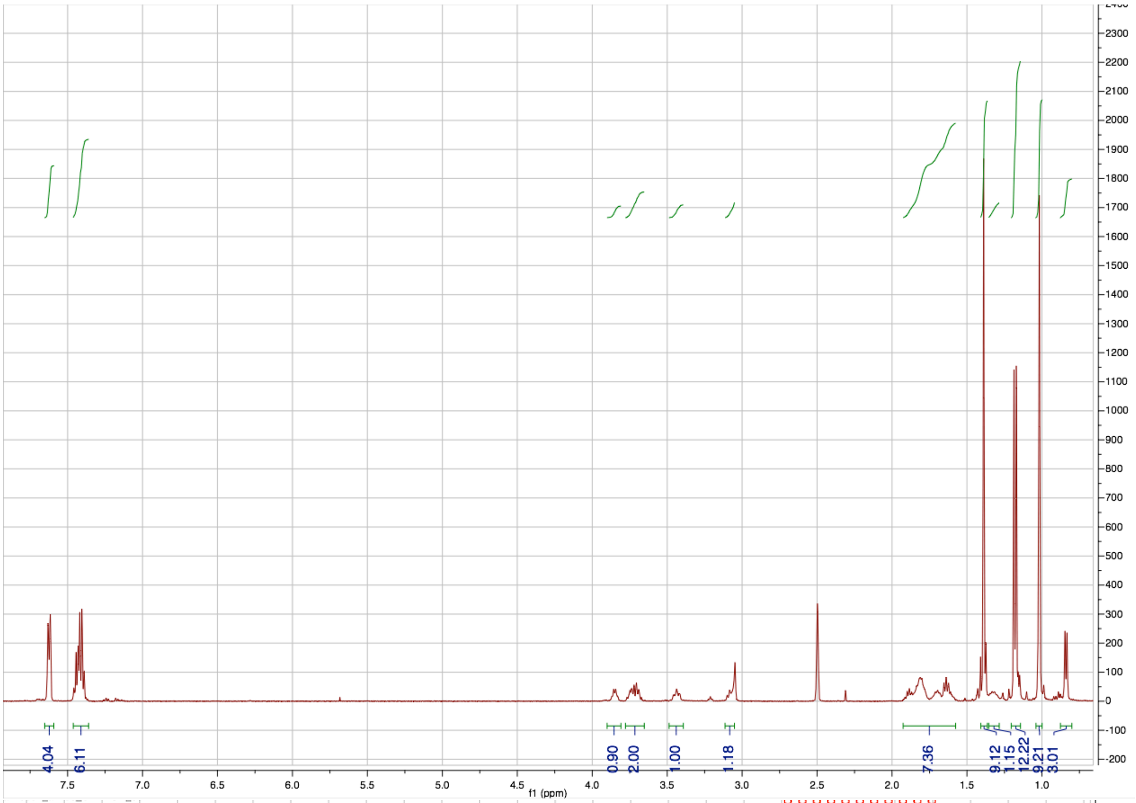

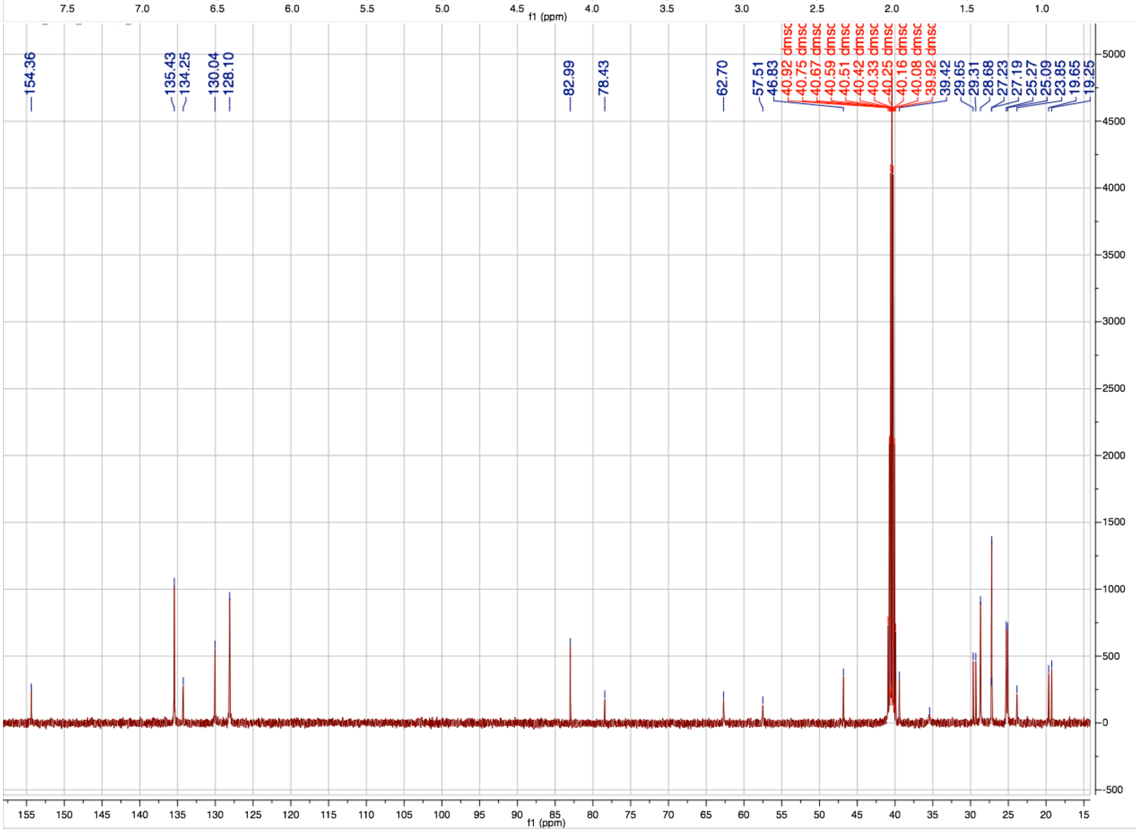

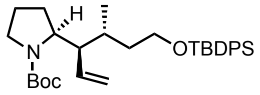

5

500 MHz, DMSO-d_6, 90 °C

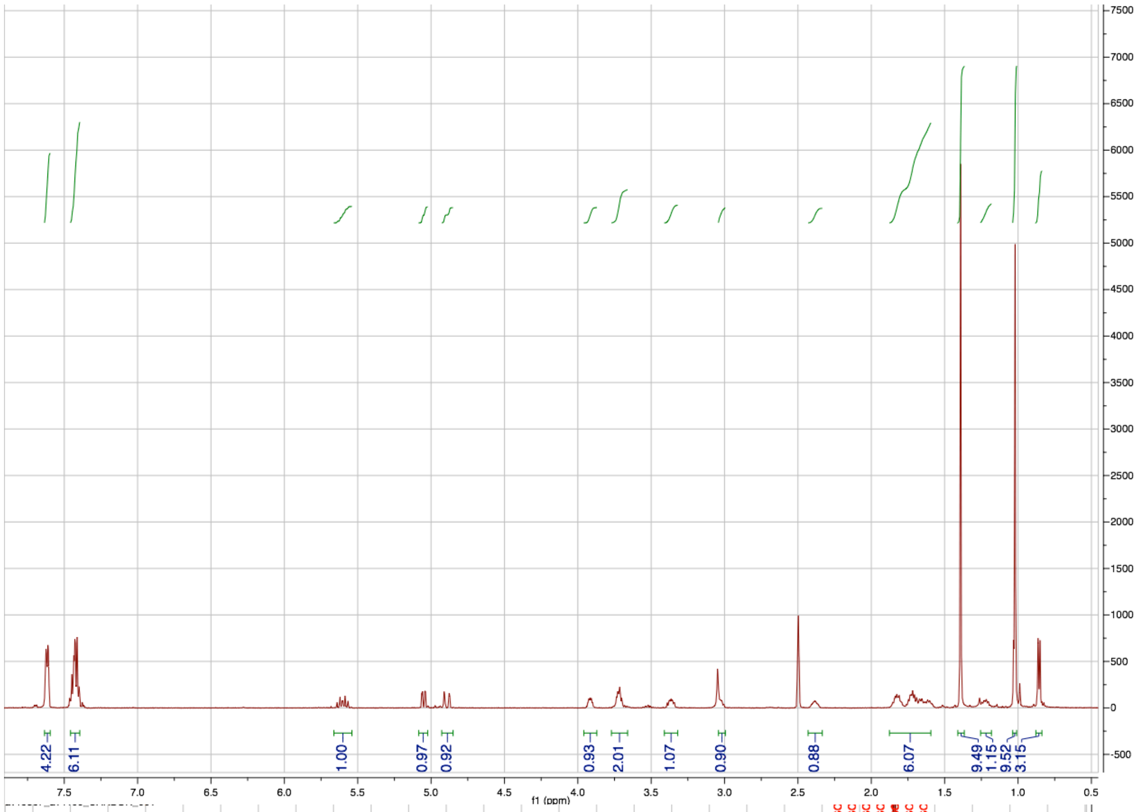

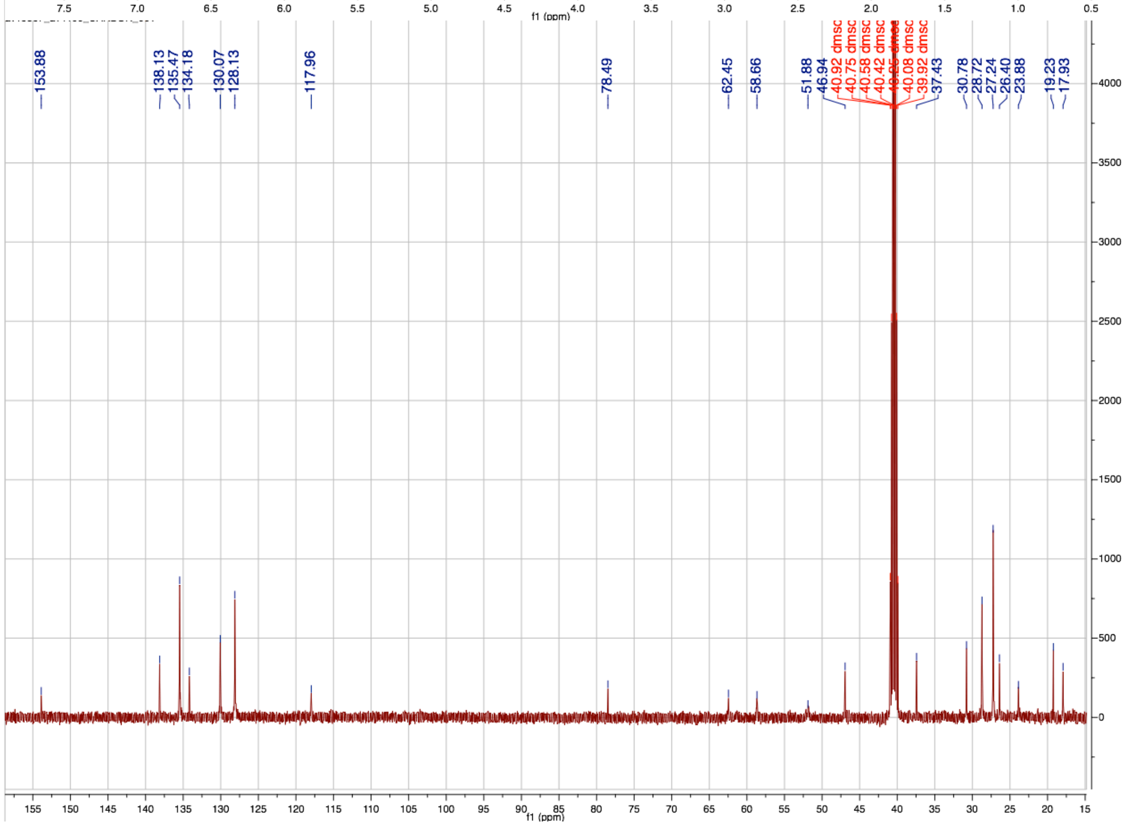

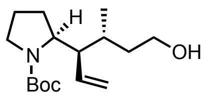

29

500 MHz, DMSO-d_6, 90 °C

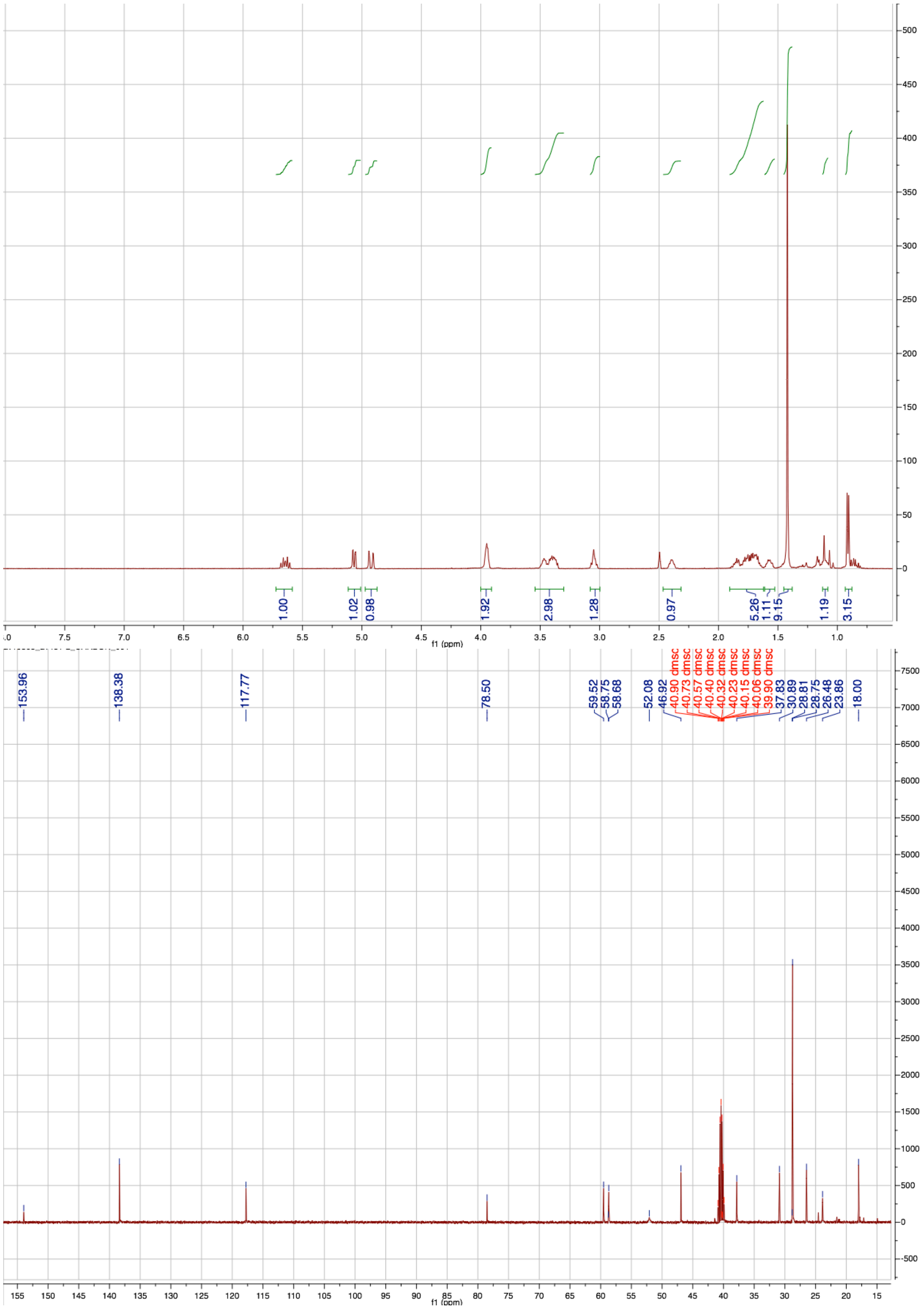

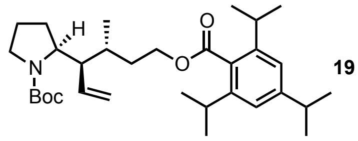

19

500 MHz, DMSO-d_6, 90 °C

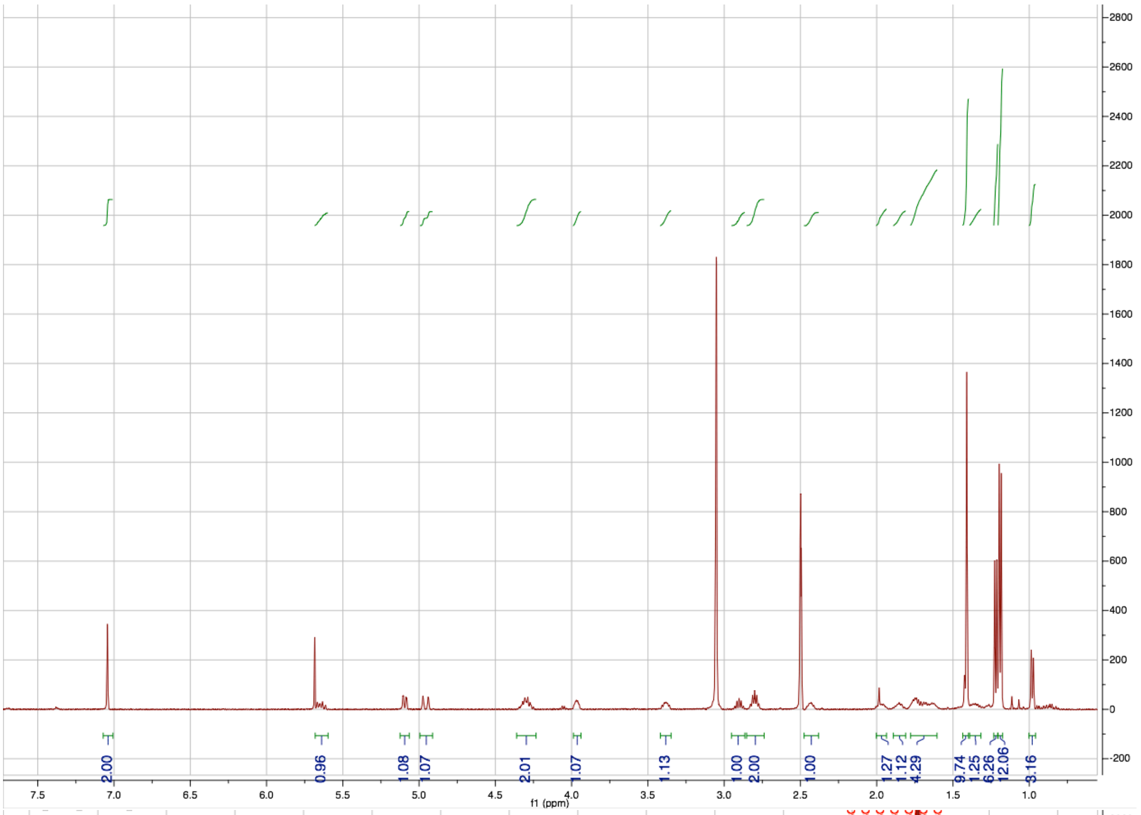

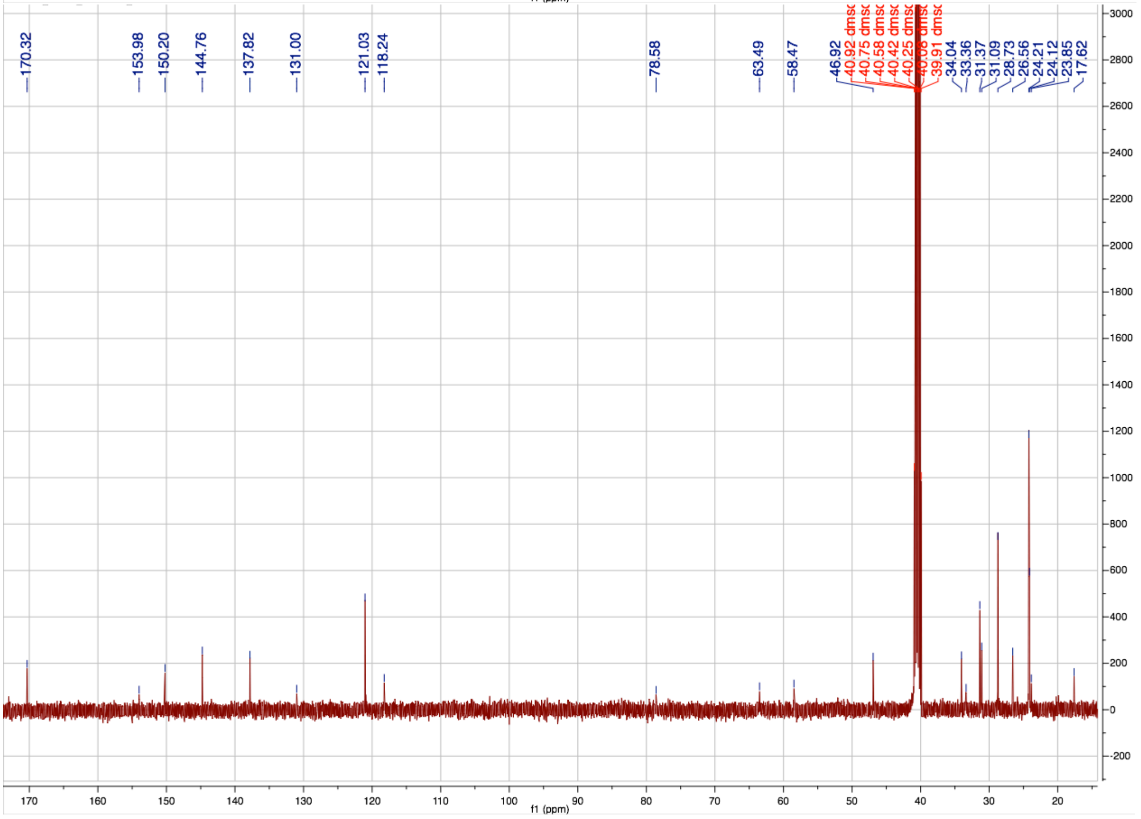

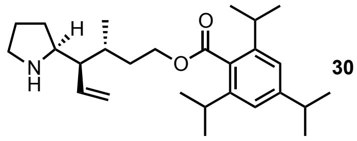

30

400 MHz, $CDCl_3$

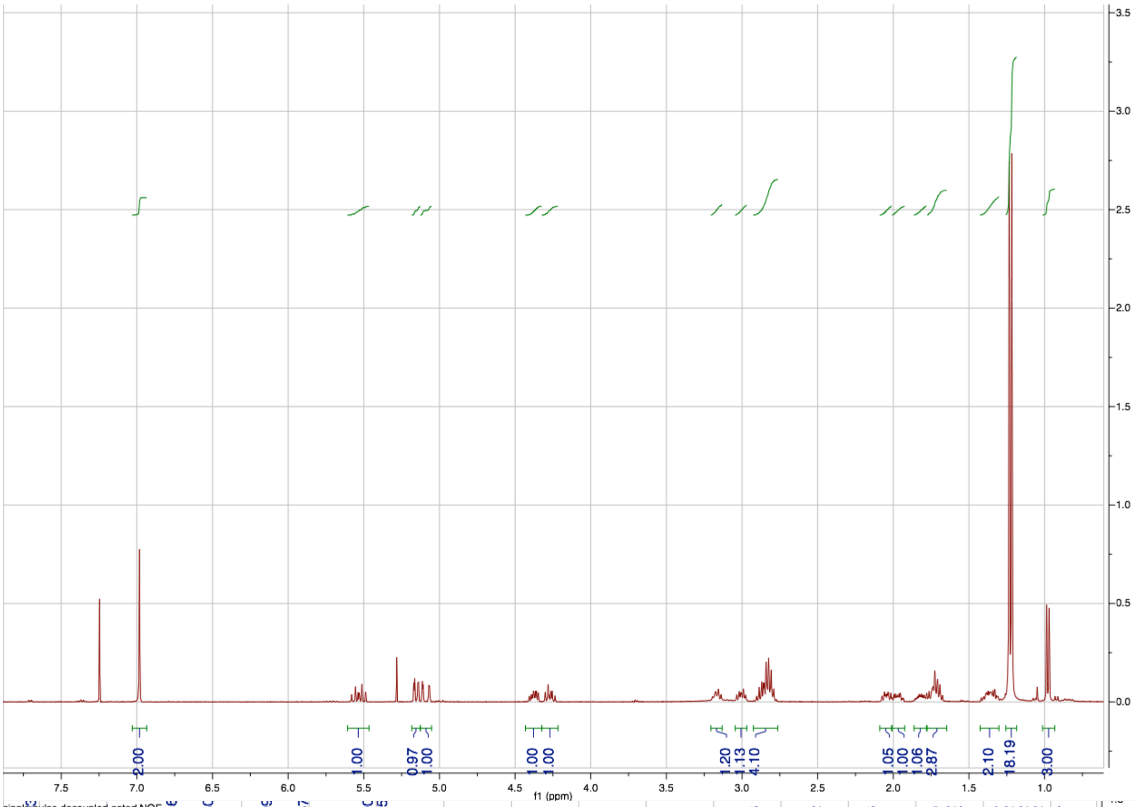

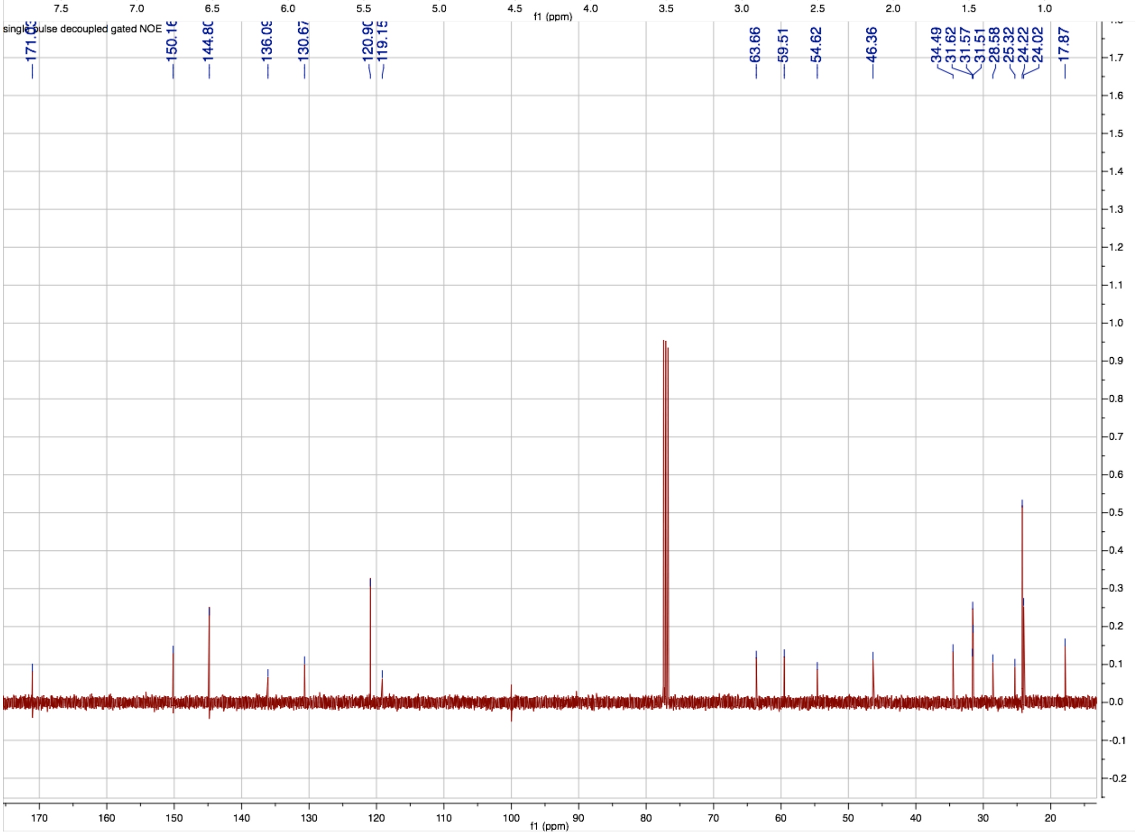

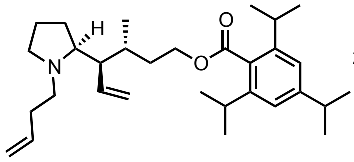

20

500 MHz, $CDCl_3$

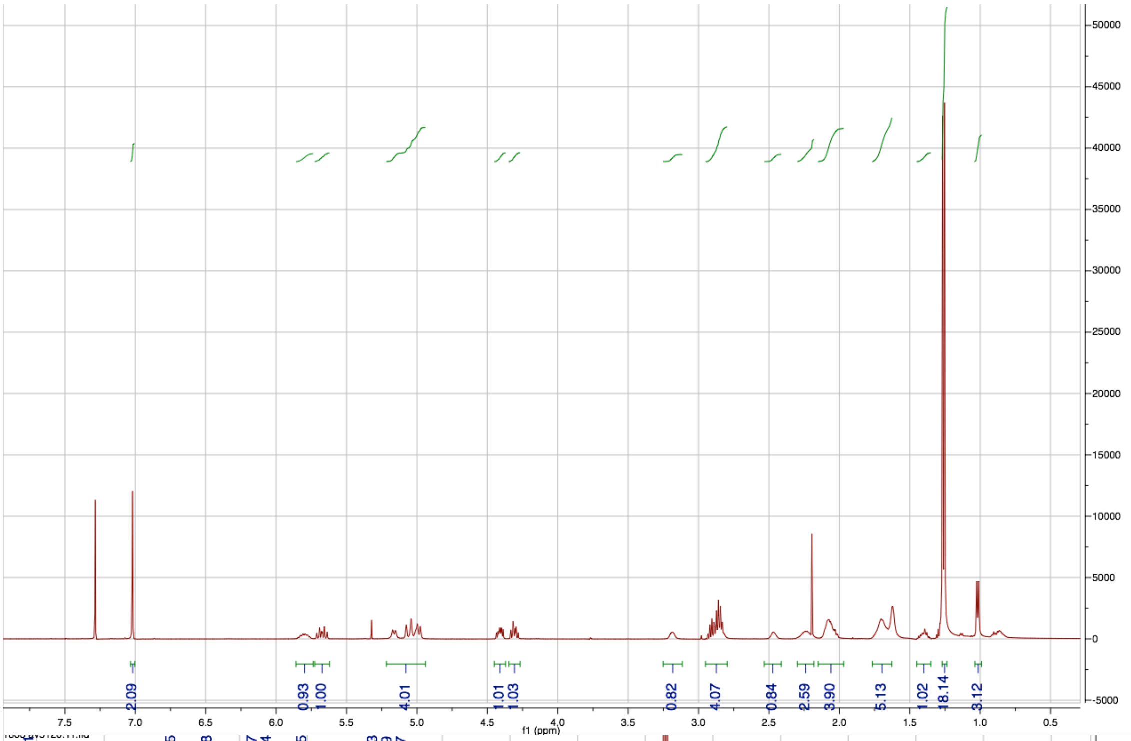

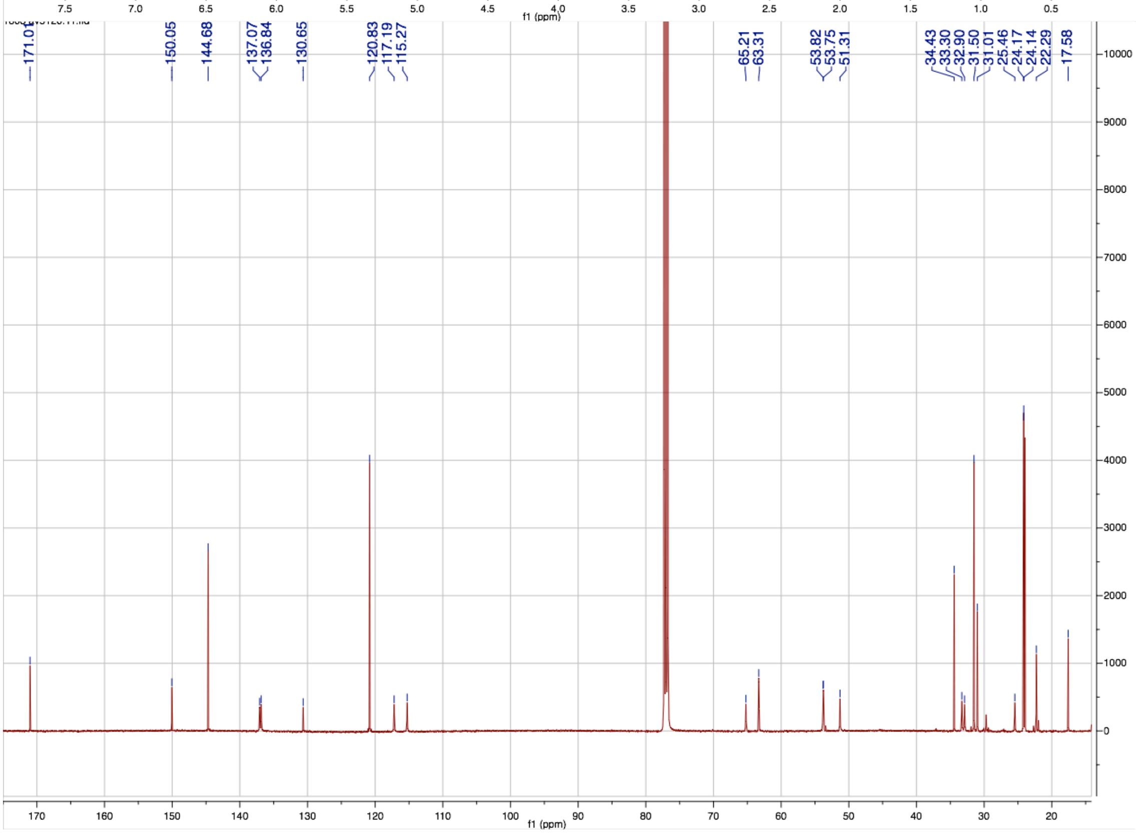

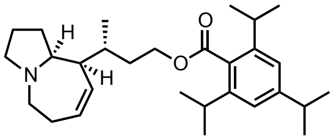

31

500 MHz, $CDCl_3$

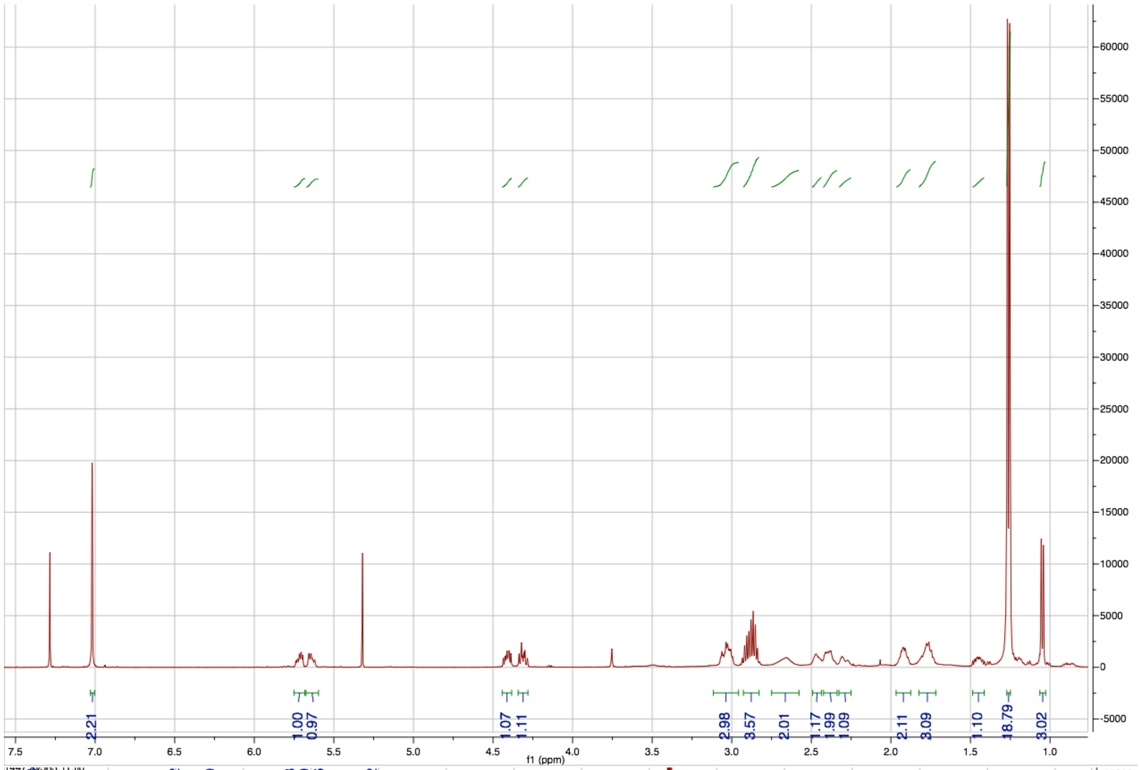

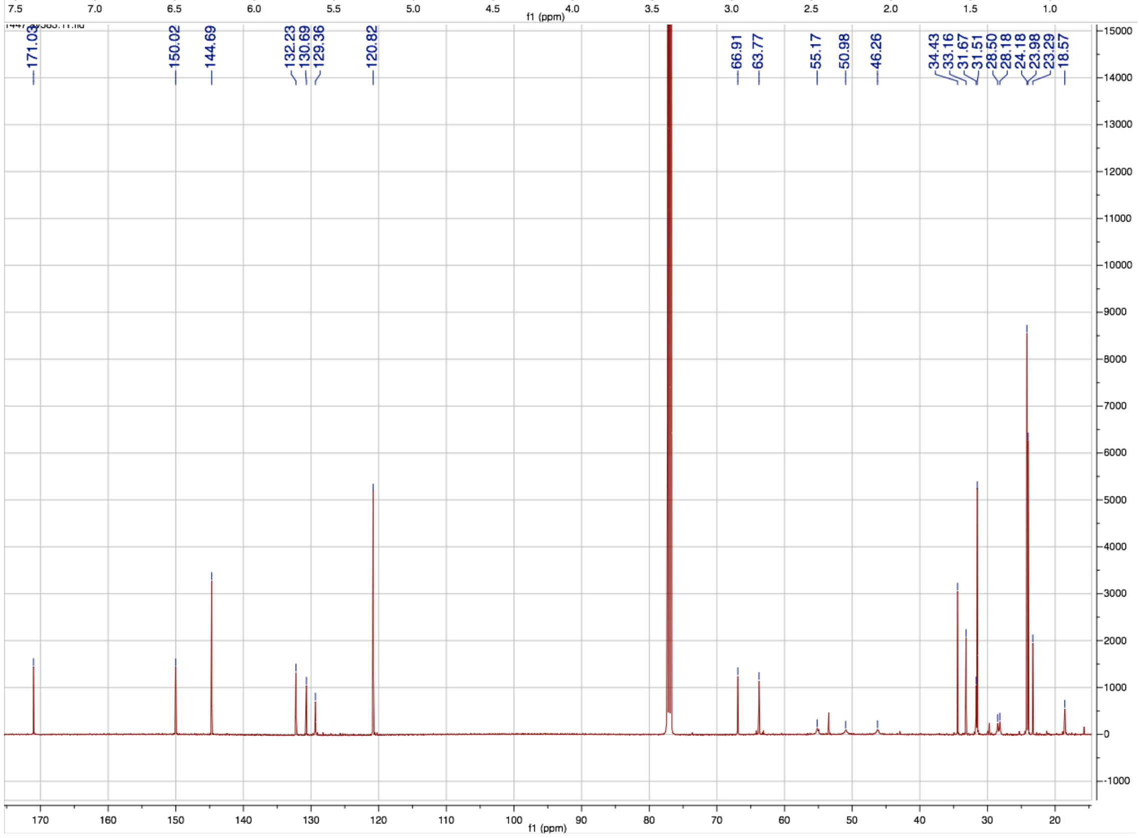

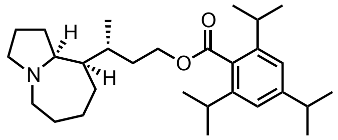

3

500 MHz, $CDCl_3$

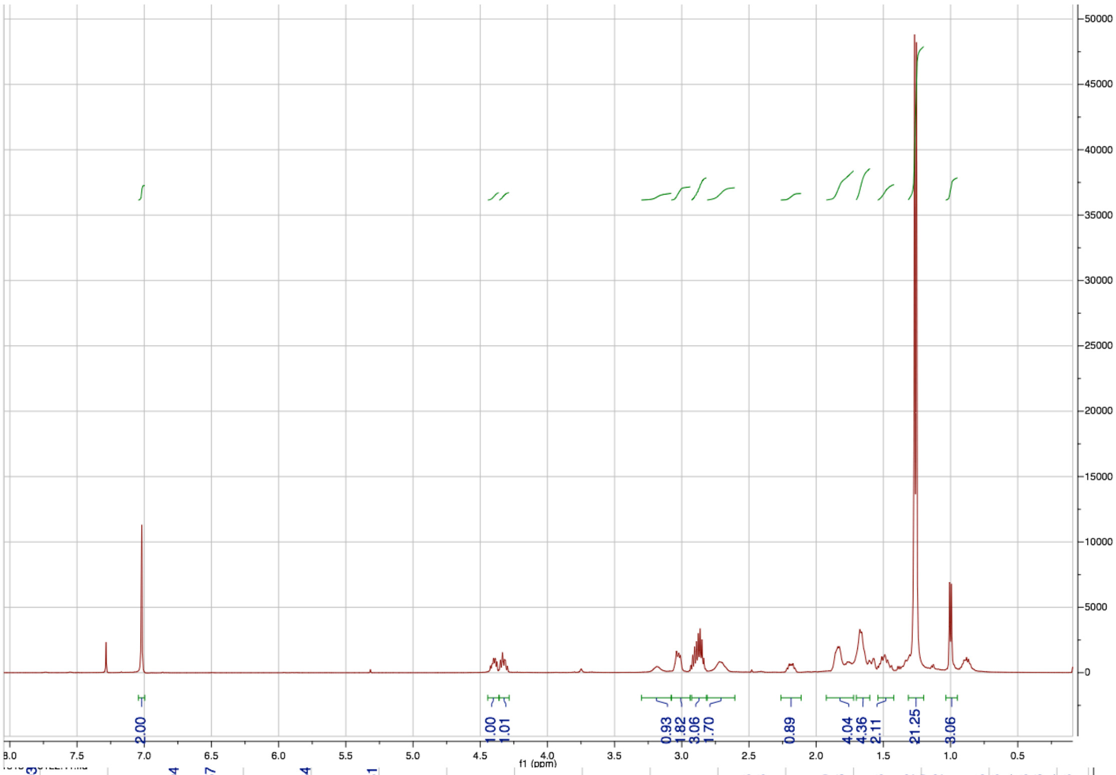

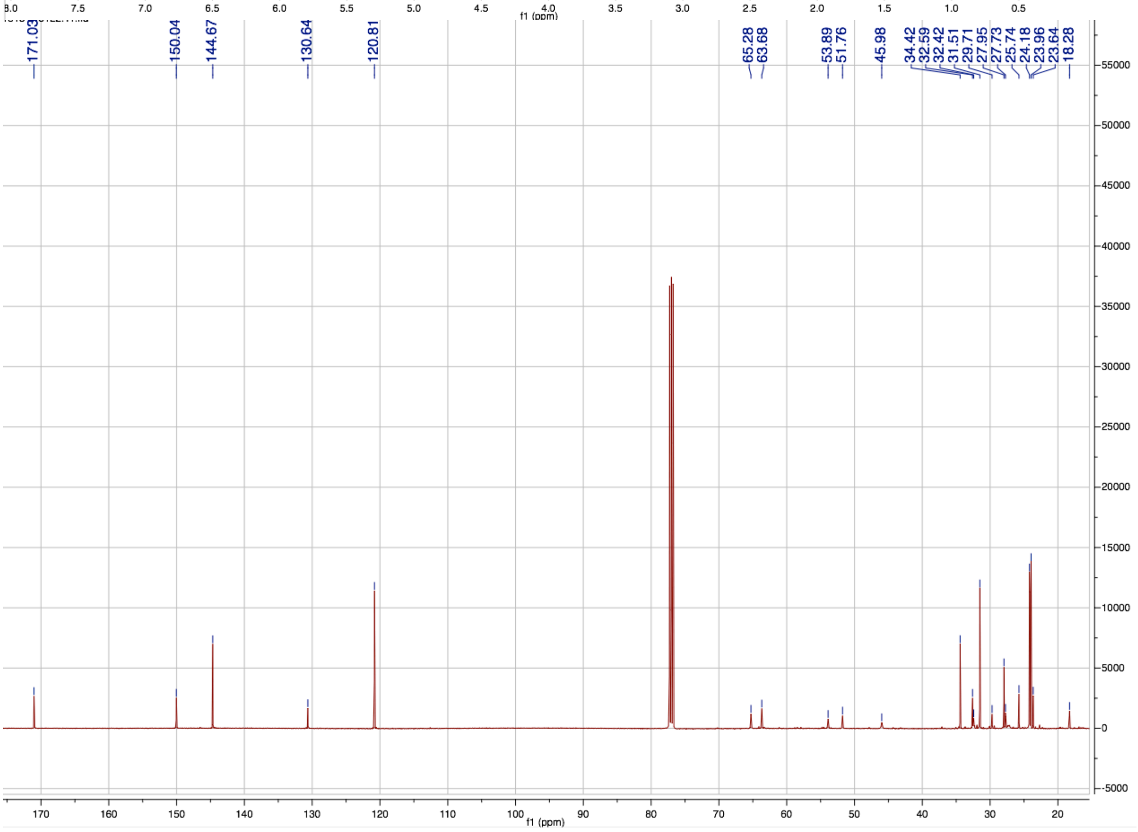

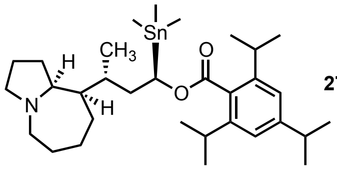

27

500 MHz, $CDCl_3$

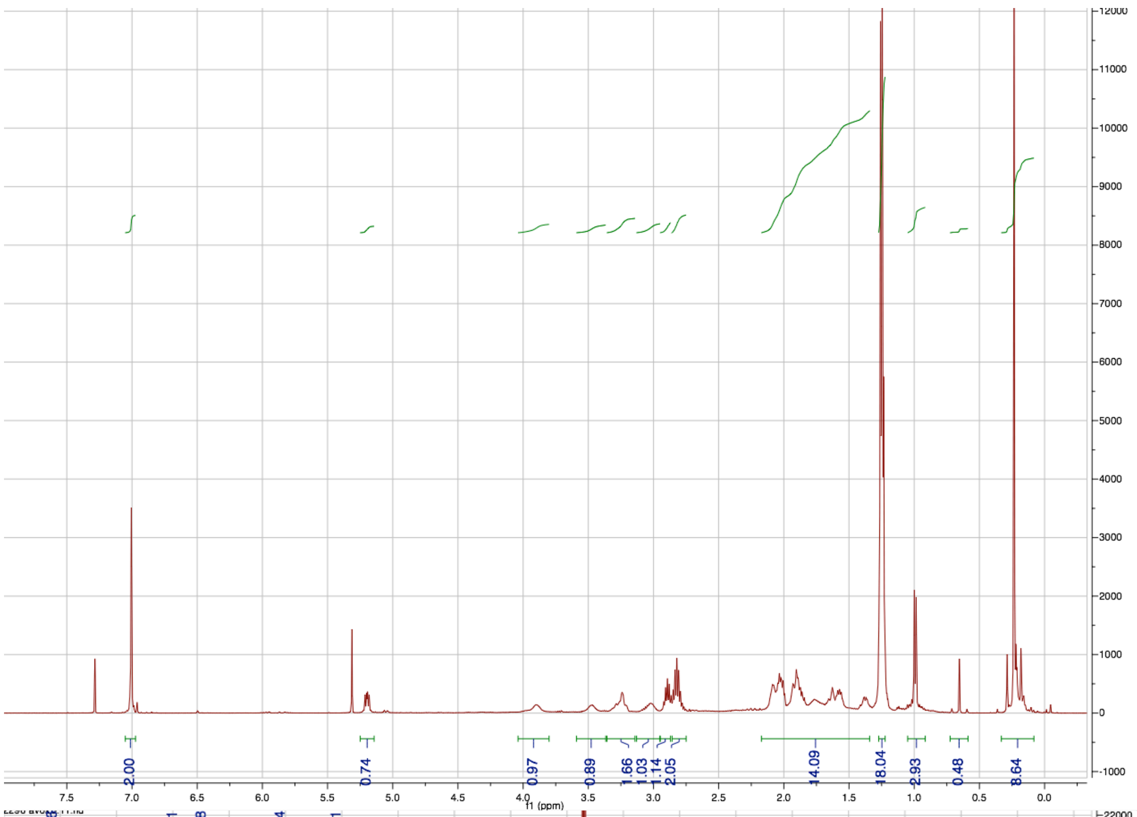

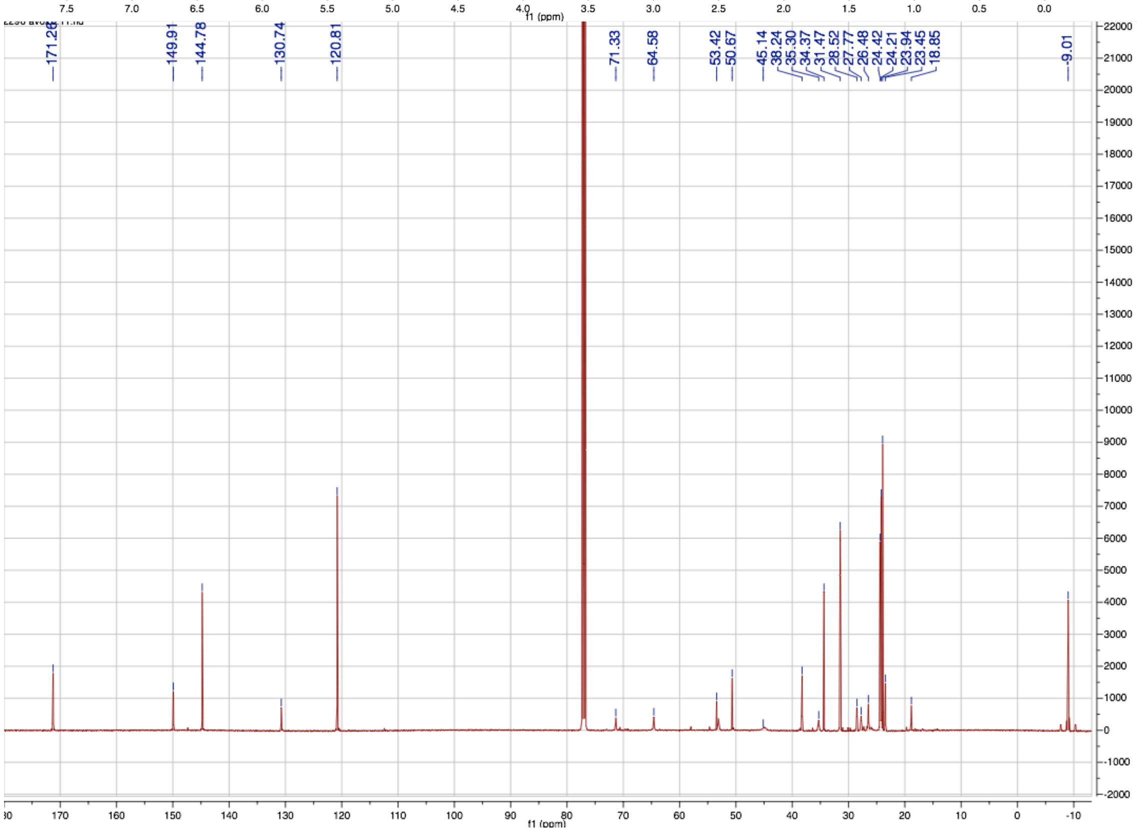

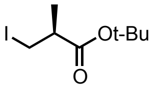

33

400 MHz, $CDCl_3$

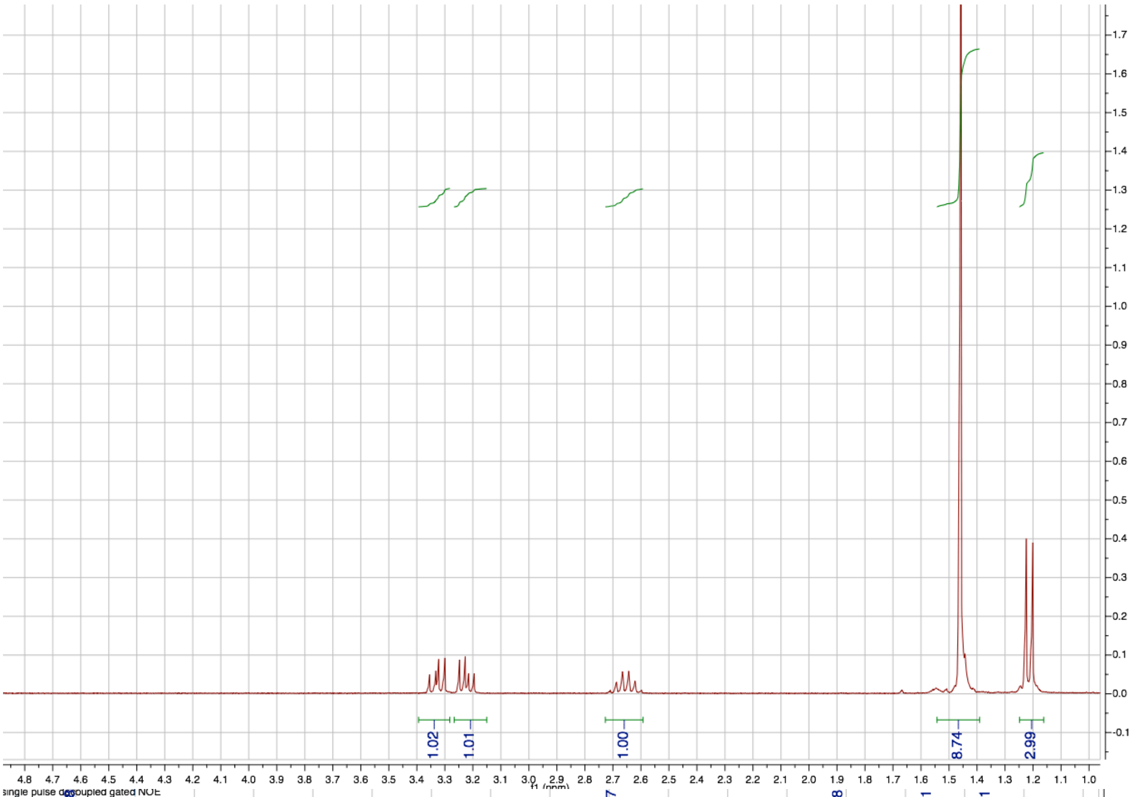

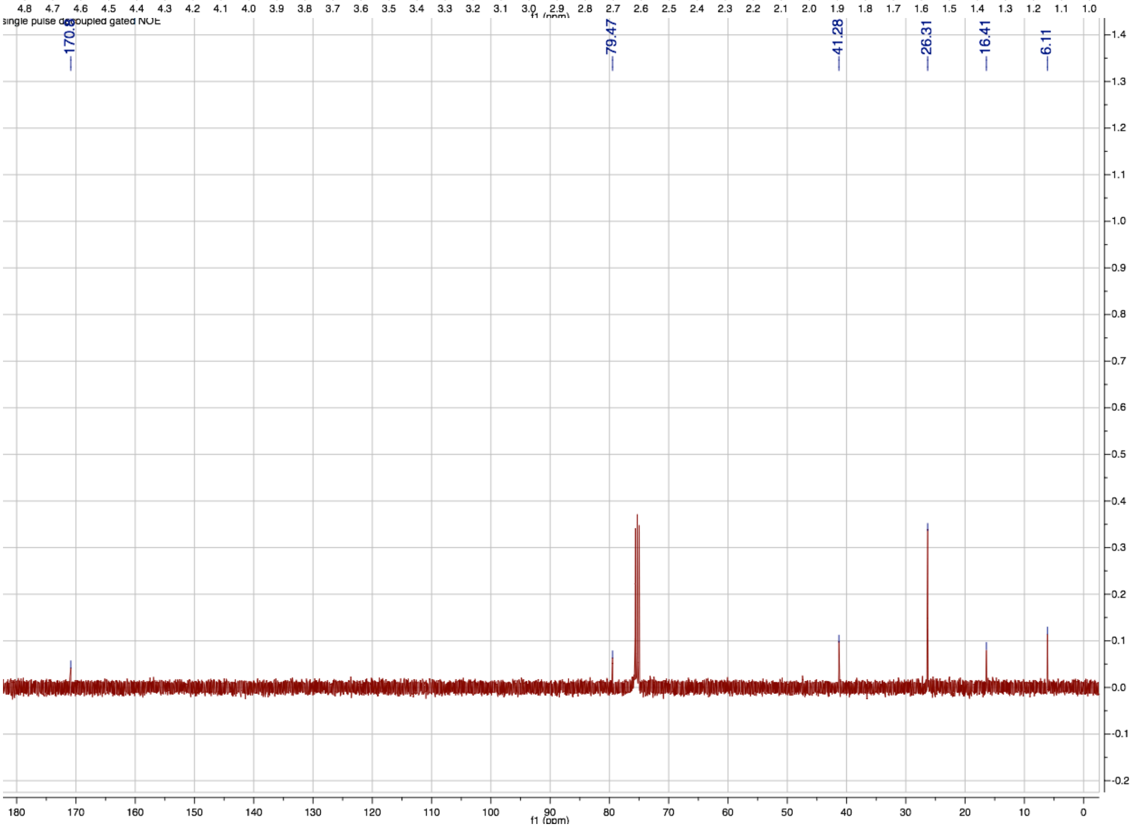

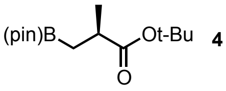

4

400 MHz, $CDCl_3$

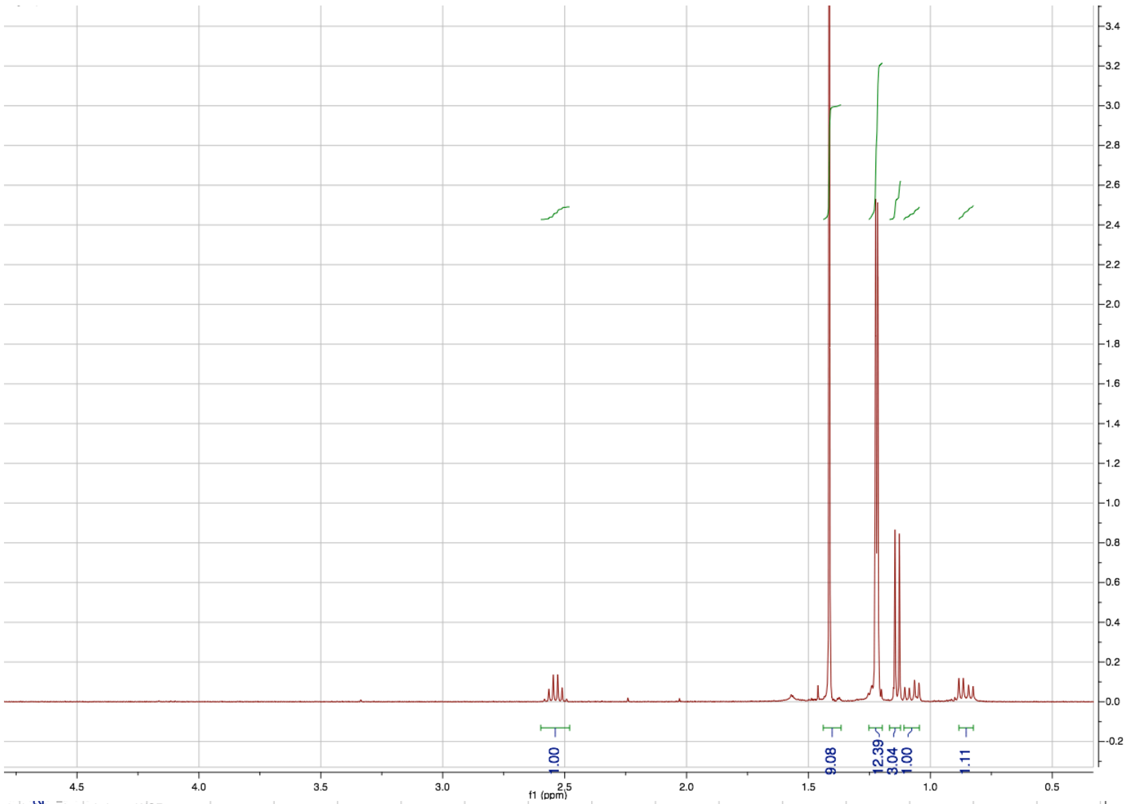

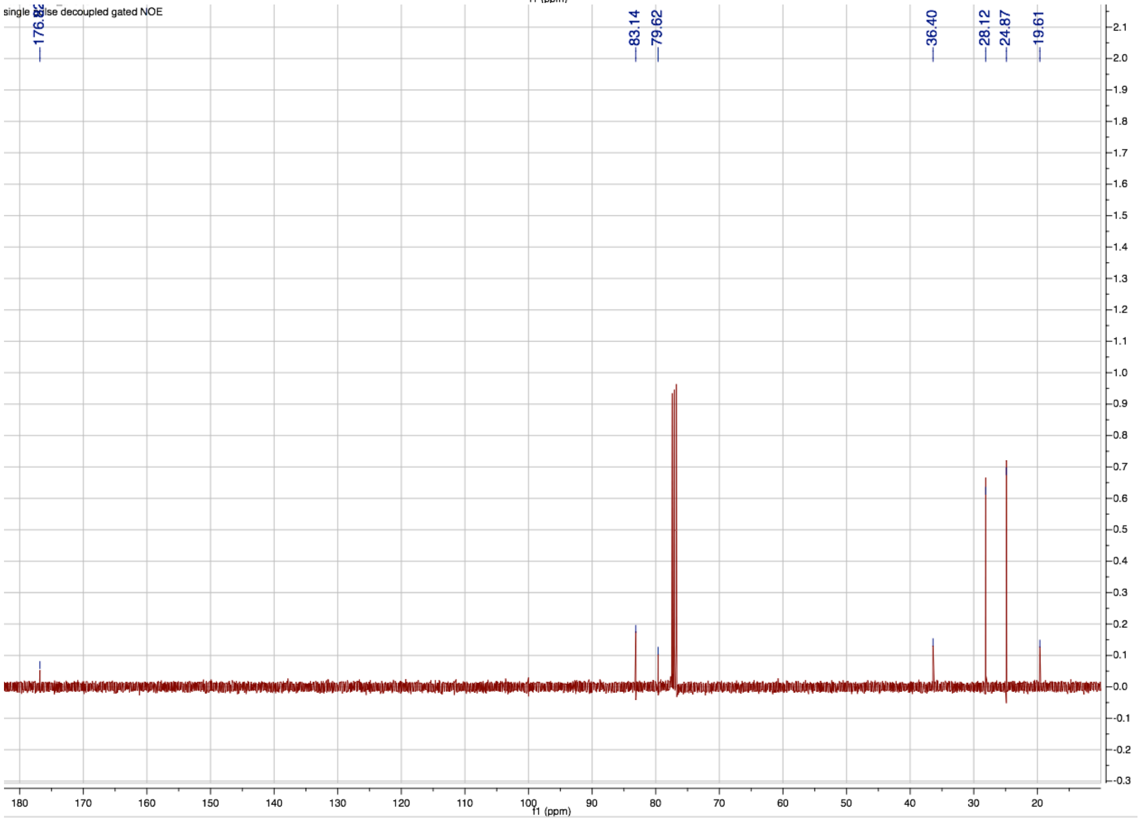

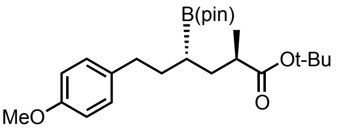

34

400 MHz, $CDCl_3$

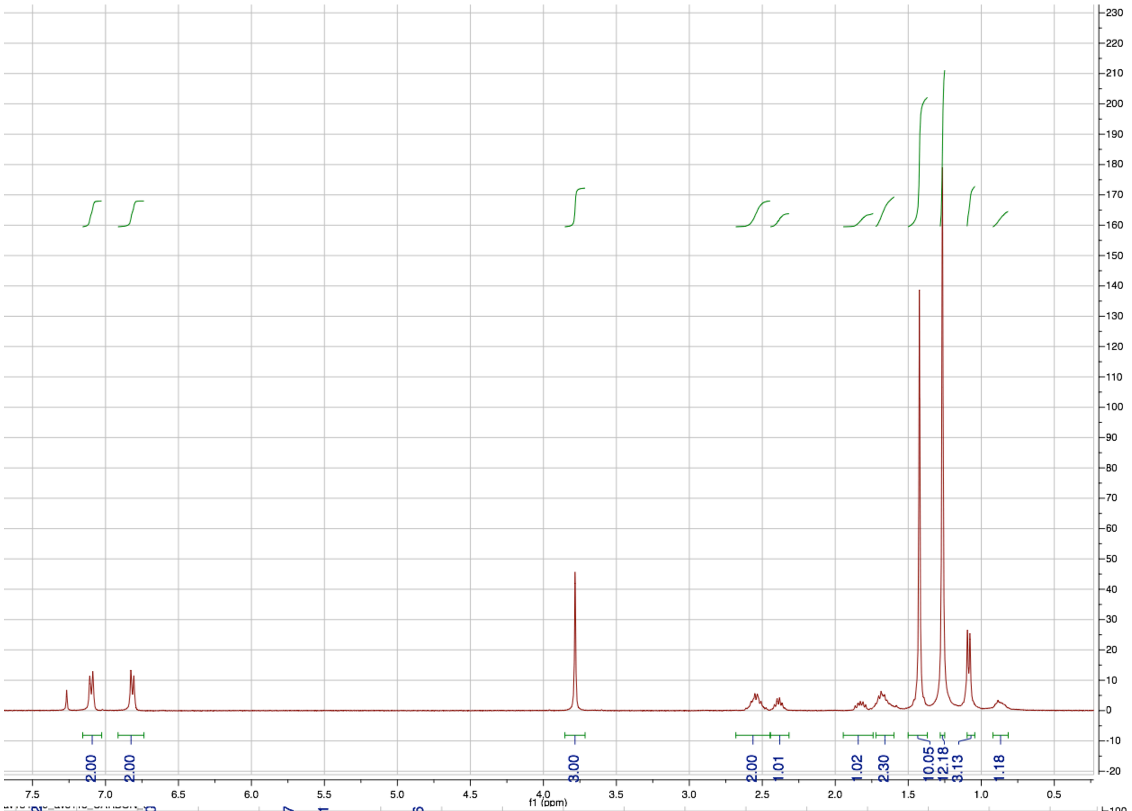

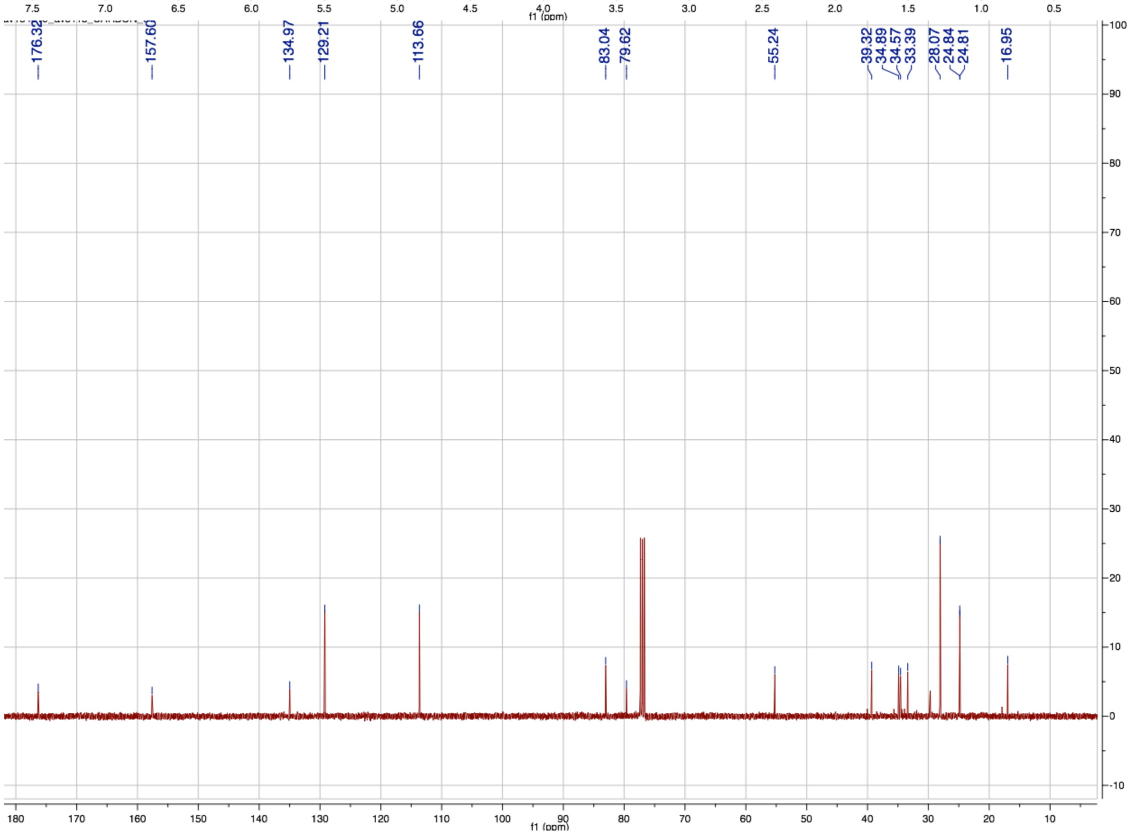

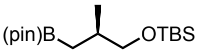

26 400 MHz, $CDCl_3$

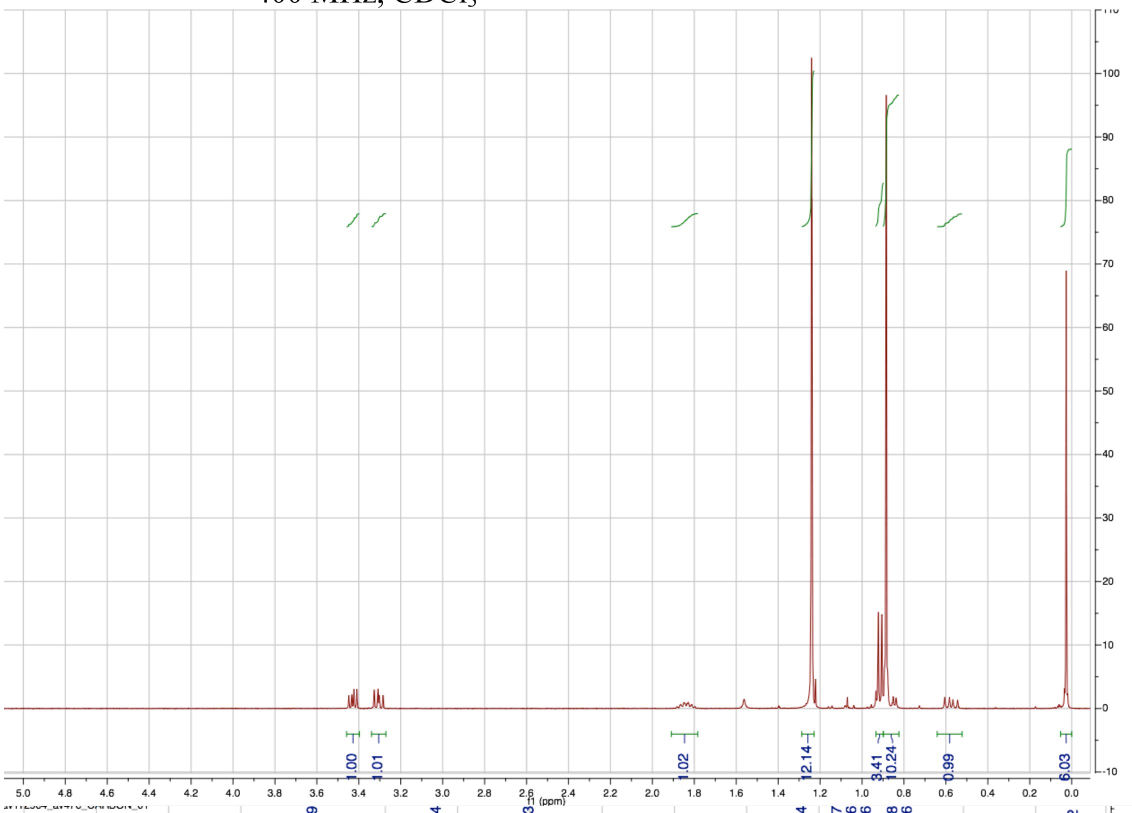

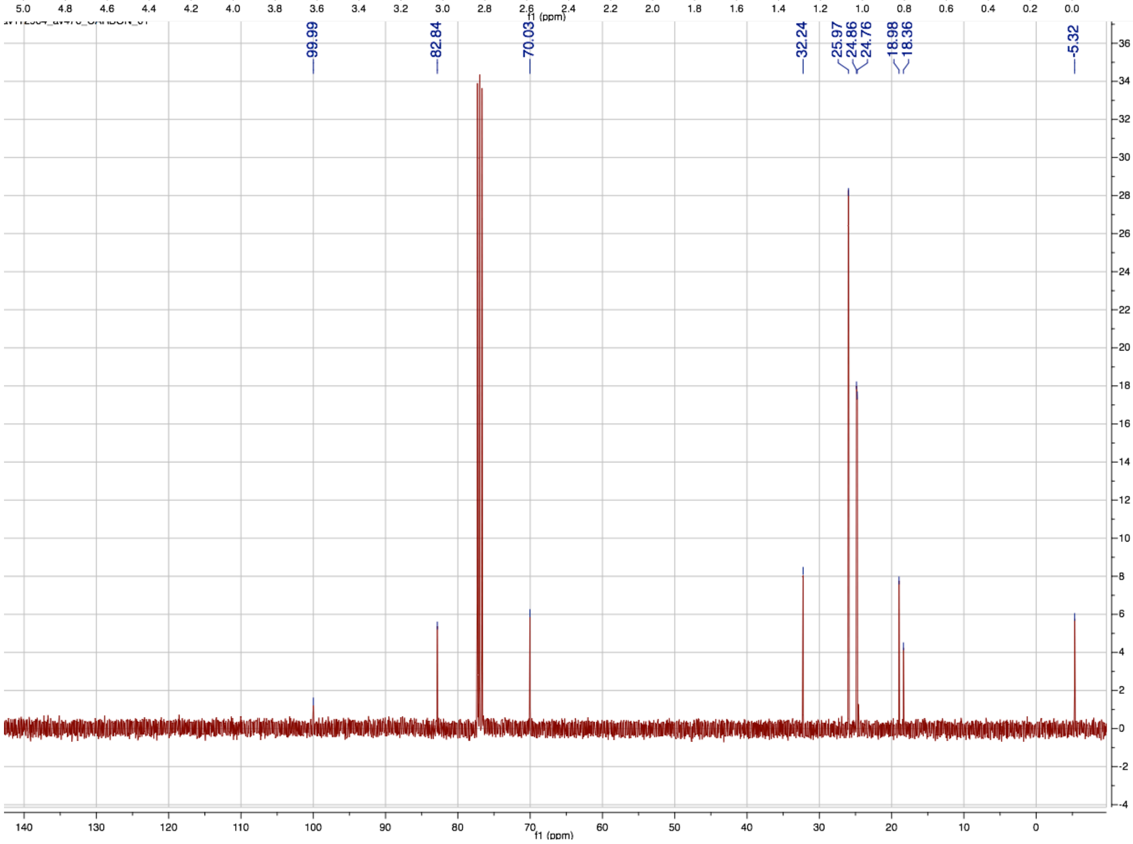

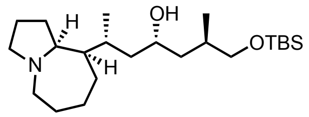

21

500 MHz, $CDCl_3$

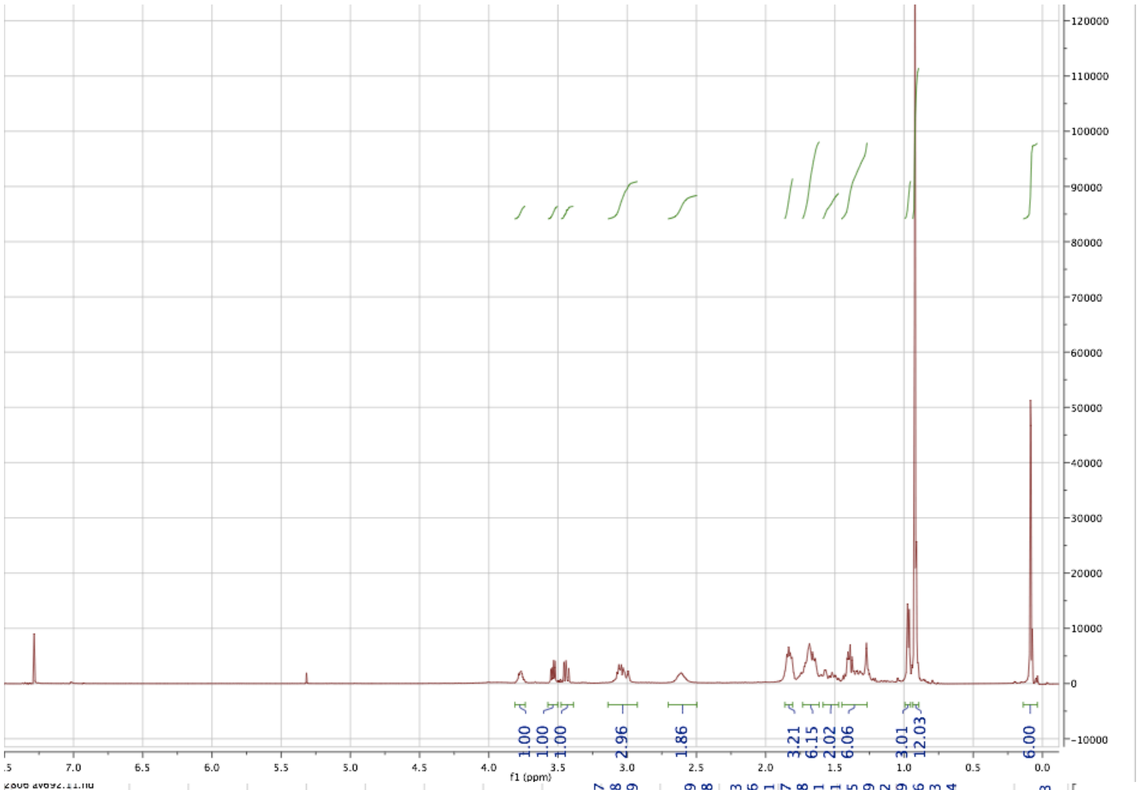

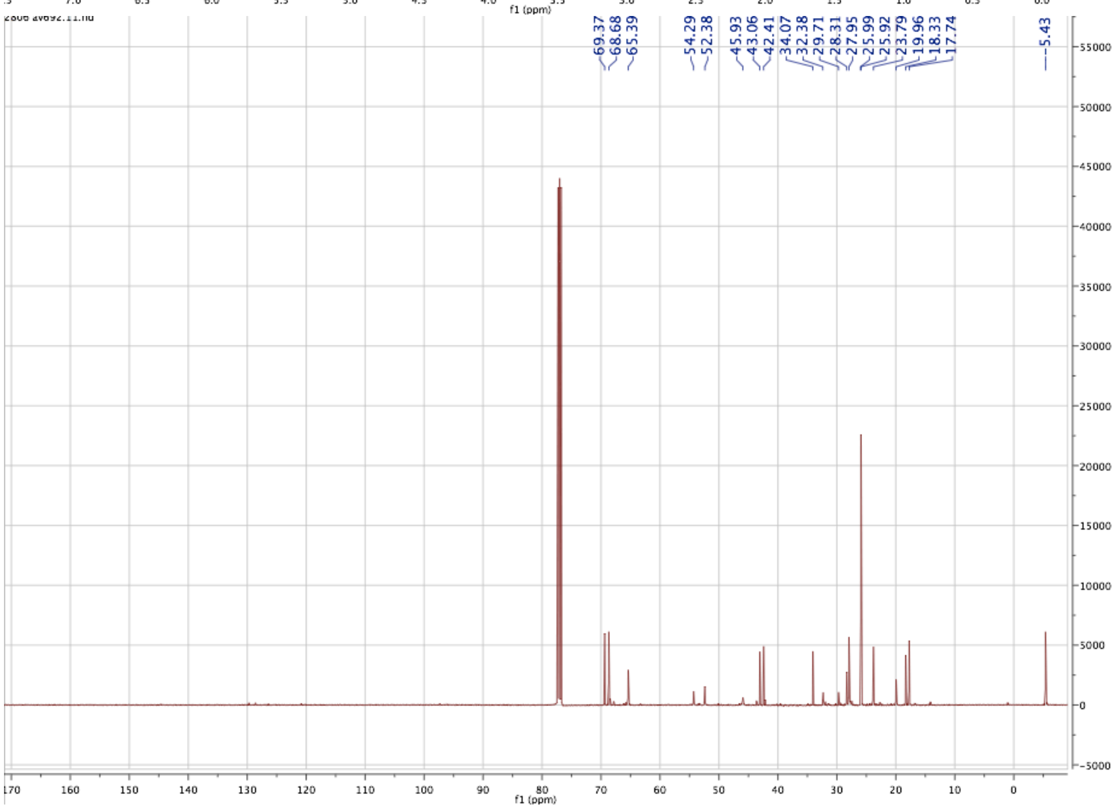

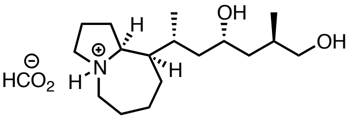

37

500 MHz, $CDCl_3$

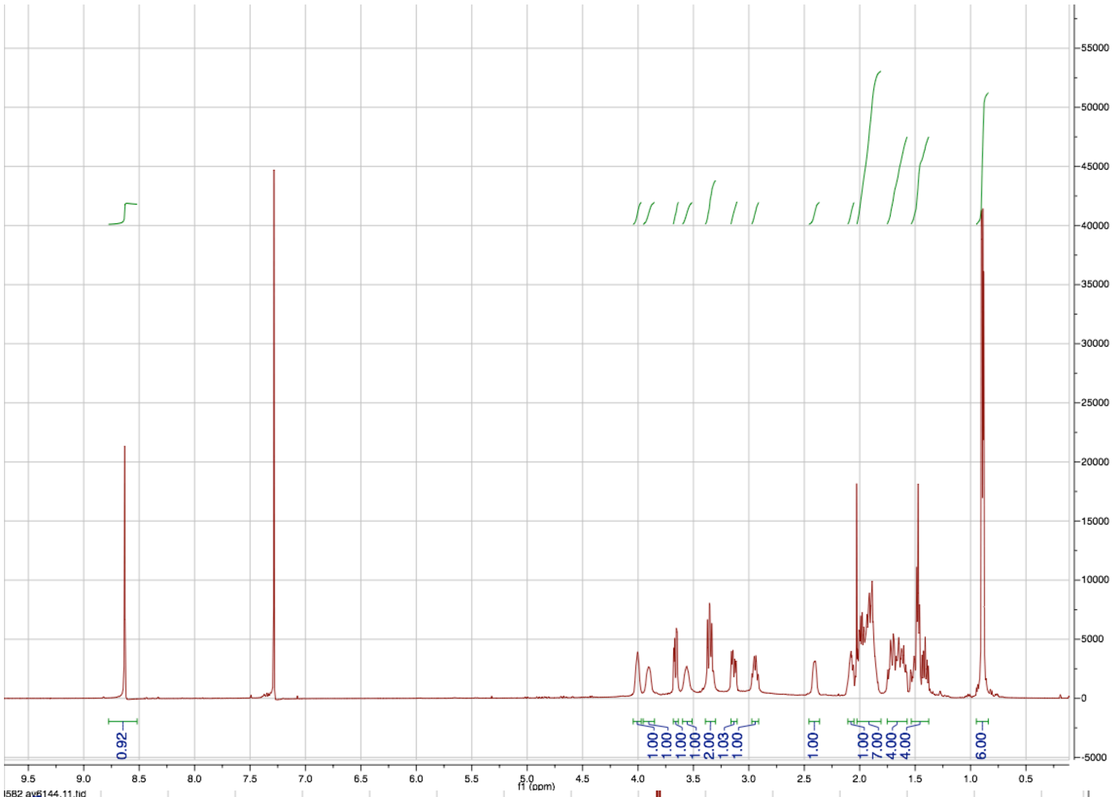

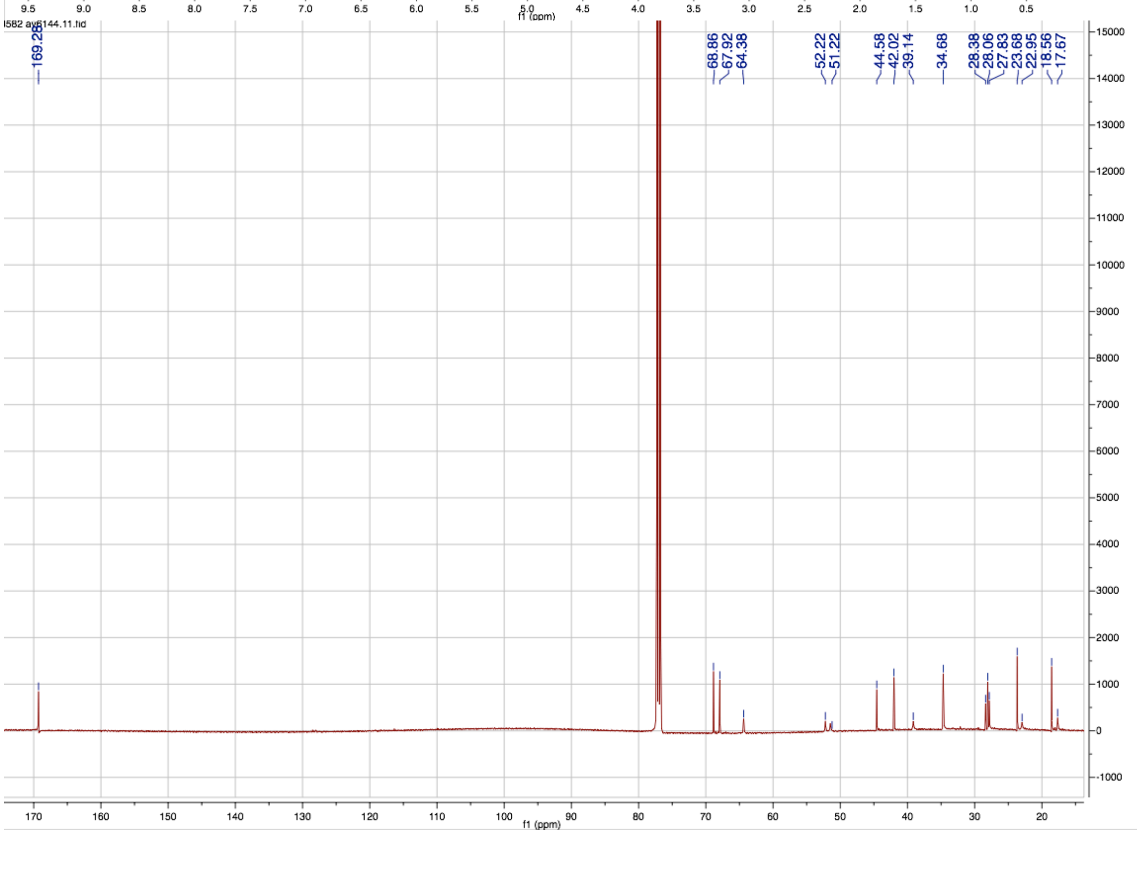

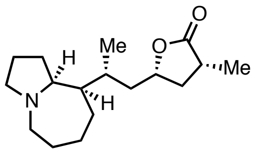

1

600 MHz, $CDCl_3$

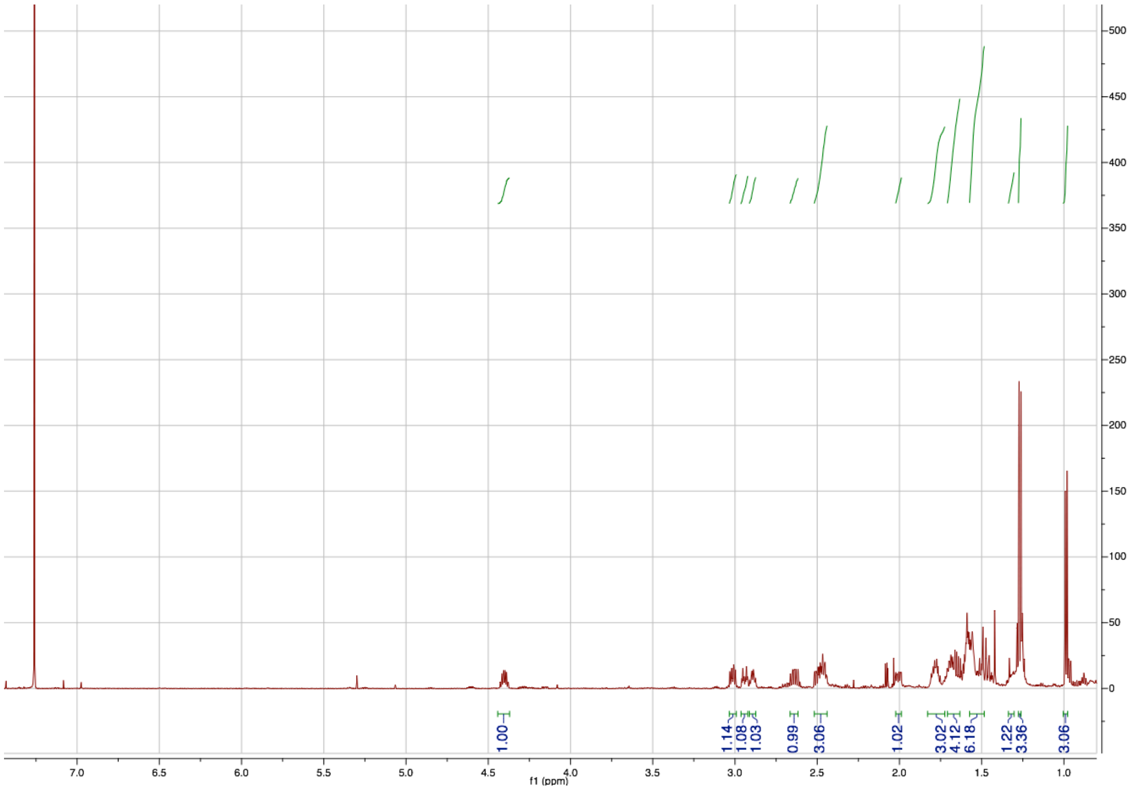

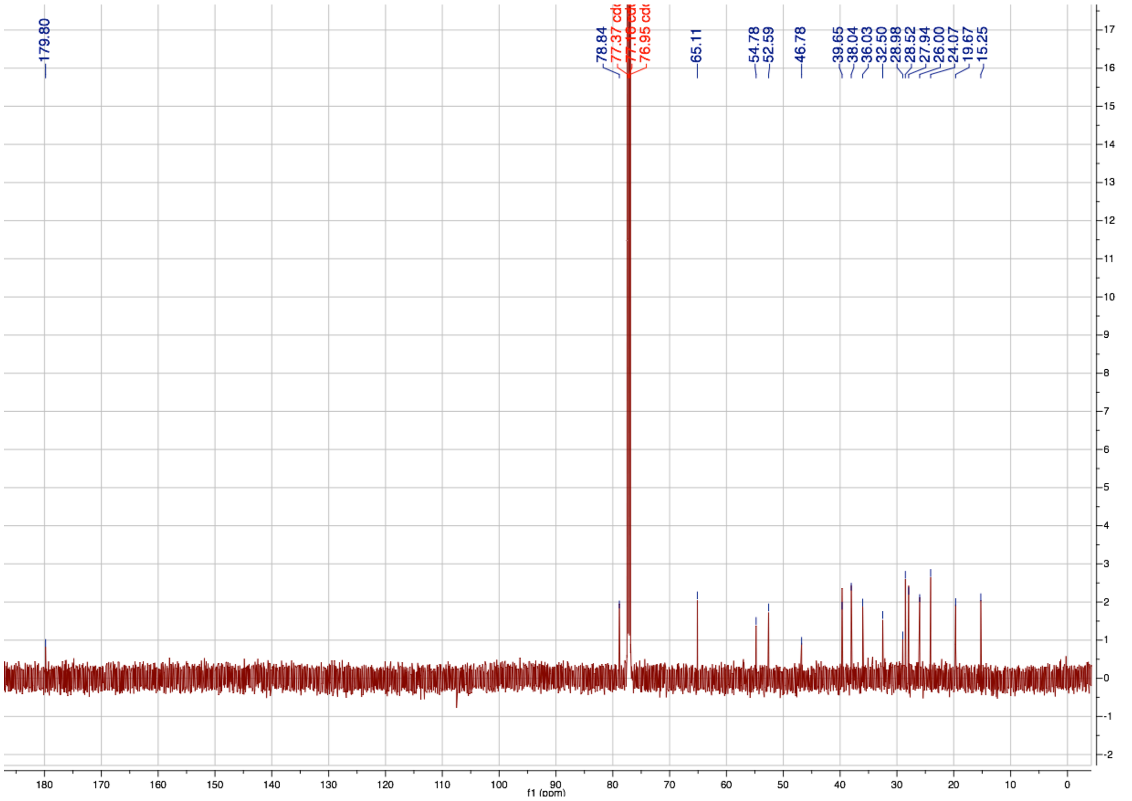

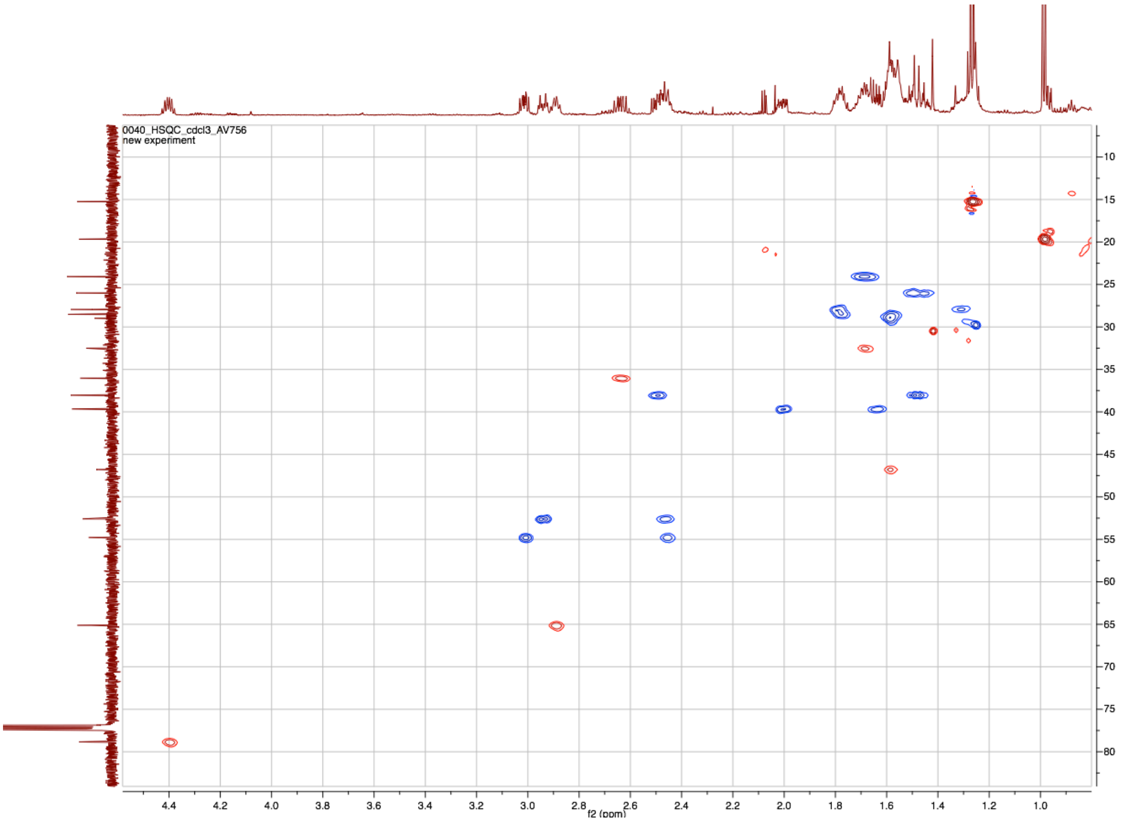
0040_HSQC_cdcl3_AV756
new experiment
f2 (ppm)

12. References

[1] N. A. Nikolic, P. Beak, *Org. Synth.* **1997**, *74*, 23-32.

[2] M. J. Dearden, C. R. Firkin, J.-P. R. Hermet, P. O'Brien, *J. Am. Chem. Soc.* **2002**, *124*, 11870-11871.

[3] A. F. Burchat, J. M. Chong, N. Nielsen, *J. Organomet. Chem.* **1997**, *542*, 281-283.

[4] R. Larouche-Gauthier, T. G. Elford, V. K. Aggarwal, *J. Am. Chem. Soc.* **2011**, *133*, 16794-16797.

[5] A. S. Batsanov, C. Grosjean, T. Schuetz, A. Whiting, *J. Org. Chem.* **2007**, *72*, 6276-6279.

[6] K. K. S. Reddy, B. V. Rao, S. S. Raju, *Tetrahedron: Asymmetry* **2011**, *22*, 662-668.

[7] M. Kurokawa, T. Shindo, M. Suzuki, N. Nakajima, K. Ishihara, T. Sugai, *Tetrahedron: Asymmetry* **2003**, *14*, 1323-1333.

[8] a) Y. Shao, L. F. Molnar, Y. Jung, J. Kussmann, C. Ochsenfeld, S. T. Brown, A. T. B. Gilbert, L. V. Slipchenko, S. V. Levchenko, D. P. O'Neill, R. A. DiStasio, Jr., R. C. Lochan, T. Wang, G. J. O. Beran, N. A. Besley, J. M. Herbert, C. Y. Lin, T. Van Voorhis, S. H. Chien, A. Sodt, R. P. Steele, V. A. Rassolov, P. E. Maslen, P. P. Korambath, R. D. Adamson, B. Austin, J. Baker, E. F. C. Byrd, H. Dachsel, R. J. Doerksen, A. Dreuw, B. D. Dunietz, A. D. Dutoi, T. R. Furlani, S. R. Gwaltney, A. Heyden, S. Hirata, C.-P. Hsu, G. Kedziora, R. Z. Khalliulin, P. Klunzinger, A. M. Lee, M. S. Lee, W. Liang, I. Lotan, N. Nair, B. Peters, E. I. Proynov, P. A. Pieniazek, Y. M. Rhee, J. Ritchie, E. Rosta, C. D. Sherrill, A. C. Simmonett, J. E. Subotnik, H. L. Woodcock, III, W. Zhang, A. T. Bell, A. K. Chakraborty, D. M. Chipman, F. J. Keil, A. Warshel, W. J. Hehre, H. F. Schaefer, III, J. Kong, A. I. Krylov, P. M. W. Gill, M. Head-Gordon, *Phys. Chem. Chem. Phys.* **2006**, *8*, 3172-3191; b) W. J. Hehre, *A guide to molecular mechanics and quantum chemical calculations*, Wavefunction, Inc., Irvine, CA, **2003**.

[9] M. J. Frisch, G. W. Trucks, H. B. Schlegel, G. E. Scuseria, M. A. Robb, J. R. Cheeseman, G. Scalmani, V. Barone, B. Mennucci, G. A. Petersson, H. Nakatsuji, M. Caricato, X. Li, H. P. Hratchian, A. F. Izmaylov, J. Bloino, G. Zheng, J. L. Sonnenberg, M. Hada, M. Ehara, K. Toyota, R. Fukuda, J. Hasegawa, M. Ishida, T. Nakajima, Y. Honda, O. Kitao, H. Nakai, T. Vreven, J. A. Montgomery, J. E. Peralta, F. Ogliaro, M. Bearpark, J. J. Heyd, E. Brothers, K. N. Kudin, V. N. Staroverov, R. Kobayashi, J. Normand, K. Raghavachari, A. Rendell, J. C. Burant, S. S. Iyengar, J. Tomasi, M. Cossi, N. Rega, J. M. Millam, M. Klene, J. E. Knox, J. B. Cross, V. Bakken, C. Adamo, J. Jaramillo, R. Gomperts, R. E. Stratmann, O. Yazyev, A. J. Austin, R. Cammi, C. Pomelli, J. W. Ochterski, R. L. Martin, K. Morokuma, V. G. Zakrzewski, G. A. Voth, P. Salvador, J. J. Dannenberg, S. Dapprich, A. D. Daniels, Farkas, J. B. Foresman, J. V. Ortiz, J. Cioslowski, D. J. Fox, Wallingford CT, **2009**.

[10] a) J. C. Slater, *Quantum Theory of Molecules and Solids, Vol. 4 (The Self-Consistent Field for Molecules and Solids)*, McGraw-Hill, **1974**; b) A. D. Becke, *Phys. Rev. A: Gen. Phys.* **1988**, *38*, 3098-3100; c) S. H. Vosko, L. Wilk, M. Nusair, *Can. J. Phys.* **1980**, *58*, 1200-1211; dC. Lee, W. Yang, R. G. Parr, *Phys. Rev. B: Condens. Matter* **1988**, *37*, 785-789.

[11] E. Glendening, A. Reed, J. Carpenter, F. Weinhold, University of Wisconsin-Madison, **1990**.

[12] R. Lagoutte, P. Sebesta, P. Jiros, B. Kalinova, A. Jirosova, J. Straka, K. Cerna, J. Sobotnik, J. Cvacka, U. Jahn, *Chem. - Eur. J.* **2013**, *19*, 8515-8524.
[13] C. Pautigny, S. Jeulin, T. Ayad, Z. Zhang, J.-P. Genêt, V. Ratovelomanana-Vidal, *Advanced Synthesis & Catalysis* **2008**, *350*, 2525-2532.
[14] K. Micoine, P. Persich, J. Llaveria, M.-H. Lam, A. Maderna, F. Loganzo, A. Fuerstner, *Chem. - Eur. J.* **2013**, *19*, 7370-7383.
[15] D. A. Hansen, C. M. Rath, E. B. Eisman, A. R. H. Narayan, J. D. Kittendorf, J. D. Mortison, Y. J. Yoon, D. H. Sherman, *J. Am. Chem. Soc.* **2013**, *135*, 11232-11238.
[16] H. Tomioka, K. Takai, K. Oshima, H. Nozaki, *Tetrahedron Lett.* **1981**, *22*, 1605-1608.
[17] P. Mungkornasawakul, S. Chaiyong, T. Sastraruji, A. Jatisatienr, C. Jatisatienr, S. G. Pyne, A. T. Ung, J. Korth, W. Lie, *Journal of Natural Products* **2009**, *72*, 848-851.
